# Integration of spatial and single-cell transcriptomic data elucidates mouse organogenesis

**DOI:** 10.1038/s41587-021-01006-2

**Published:** 2021-09-06

**Authors:** T. Lohoff, S. Ghazanfar, A. Missarova, N. Koulena, N. Pierson, J. A. Griffiths, E. S. Bardot, C.-H. L. Eng, R. C. V. Tyser, R. Argelaguet, C. Guibentif, S. Srinivas, J. Briscoe, B. D. Simons, A.-K. Hadjantonakis, B. Göttgens, W. Reik, J. Nichols, L. Cai, J. C. Marioni

**Affiliations:** 1 Wellcome-Medical Research Council Cambridge Stem Cell Institute, University of Cambridge, Cambridge, UK; 2 Department of Physiology, Development and Neuroscience, University of Cambridge, Cambridge, UK; 3 Epigenetics Programme, Babraham Institute, Cambridge, UK; 4 Cancer Research UK Cambridge Institute, University of Cambridge, Cambridge, UK; 5 European Molecular Biology Laboratory, European Bioinformatics Institute, Wellcome Genome Campus, Cambridge, UK; 6 Division of Biology and Biological Engineering, California Institute of Technology, Pasadena, CA USA; 7 Developmental Biology Program, Sloan Kettering Institute, Memorial Sloan Kettering Cancer Center, New York, NY USA; 8 Department of Physiology, Anatomy and Genetics, University of Oxford, Oxford, UK; 9 Department of Haematology, University of Cambridge, Cambridge, UK; 10 Sahlgrenska Center for Cancer Research, Department of Microbiology and Immunology, University of Gothenburg, Gothenburg, Sweden; 11 The Francis Crick Institute, London, UK; 12 The Wellcome/Cancer Research UK Gurdon Institute, University of Cambridge, Cambridge, UK; 13 Department of Applied Mathematics and Theoretical Physics, Centre for Mathematical Sciences, University of Cambridge, Cambridge, UK; 14 Centre for Trophoblast Research, University of Cambridge, Cambridge, UK; 15 Wellcome Sanger Institute, Wellcome Genome Campus, Cambridge, UK; 16 Present Address: Genomics Plc, Cambridge, UK

**Keywords:** Computational biology and bioinformatics, Embryogenesis

## Abstract

Molecular profiling of single cells has advanced our knowledge of the molecular basis of development. However, current approaches mostly rely on dissociating cells from tissues, thereby losing the crucial spatial context of regulatory processes. Here, we apply an image-based single-cell transcriptomics method, sequential fluorescence in situ hybridization (seqFISH), to detect mRNAs for 387 target genes in tissue sections of mouse embryos at the 8–12 somite stage. By integrating spatial context and multiplexed transcriptional measurements with two single-cell transcriptome atlases, we characterize cell types across the embryo and demonstrate that spatially resolved expression of genes not profiled by seqFISH can be imputed. We use this high-resolution spatial map to characterize fundamental steps in the patterning of the midbrain–hindbrain boundary (MHB) and the developing gut tube. We uncover axes of cell differentiation that are not apparent from single-cell RNA-sequencing (scRNA-seq) data, such as early dorsal–ventral separation of esophageal and tracheal progenitor populations in the gut tube. Our method provides an approach for studying cell fate decisions in complex tissues and development.

## Abstract (web summary)

Improved integration of spatial and single-cell transcriptomic data provides insights into mouse development.

## Main

Lineage priming, cell fate specification and tissue patterning during early mammalian development are complex processes involving signals from surrounding tissues, mechanical constraints, and transcriptional and epigenetic changes, which together prompt the adoption of unique cell fates^1–7^. All these factors play key roles in gastrulation, the process by which the three germ layers emerge and the body axis is established. Subsequently, the germ layer progenitors, formed during gastrulation, will give rise to all major organs in a process known as organogenesis.

Recently, scRNA-seq and other single-cell genomic approaches have been used to investigate how the molecular landscape of cells within the mouse embryo changes during early development. These methods have provided insights into how symmetry breaking of the epiblast population leads to commitment to different fates as the embryo passes through gastrulation and on to organogenesis^1–3,6–14^. By computationally ordering cells through their differentiation (‘pseudotime’), an understanding of the molecular changes that underpin cell-type development has been obtained, providing insight into the underlying regulatory mechanisms, including the role of the epigenome. Recently, technological advances have enabled scRNA-seq to be performed alongside CRISPR–Cas9 scarring, thus simultaneously documenting a cell’s molecular state and lineage. Such approaches have been applied to track zebrafish development^15–17^ and more recently mouse embryogenesis^9,18^. Together, these experimental strategies have enhanced our understanding of developmental lineage relationships and the associated molecular changes.

However, to date, single-cell genomics studies of early mammalian development have focused on profiling dissociated populations of cells, where spatial information is lost. Although regions of the embryo have been microdissected and profiled using small cell number RNA-sequencing protocols, these approaches neither scale to later stages of development nor do they provide single-cell resolution, which may be critical given the role of local environmental cues in conditioning cell fate and patterning^8,13,19^. By contrast, in situ hybridization, single-molecule RNA FISH (smFISH) and other related approaches allow gene expression levels to be measured within a defined spatial context. However, these approaches are typically limited to either quantifying expression patterns in broad domains^20,21^ or to studying a limited number of genes, thus precluding the generation of comprehensive cell resolution maps of expression across an entire embryo. Recent technological advances promise to overcome these limitations; approaches that exploit highly multiplexed RNA FISH^22–27^, that perform sequencing on intact tissues^28–30^, or that hybridize tissue sections to spatially barcoded microarrays^31,32^ promise to simultaneously profile the expression of hundreds or thousands of genes within single cells whose spatial location is preserved.

Here, using an existing scRNA-seq atlas covering stages of mouse development from gastrulation to early organogenesis^6^ (‘Gastrulation atlas’), we designed probes against a panel of 387 genes and spatially localized their expression in multiple embryo sections at the 8–12 somite stage (ss) using a version of the seqFISH method modified to allow highly effective cell segmentation. Assigning each cell in the seqFISH-profiled embryos a distinct cell-type identity revealed different patterns of colocalization of cells within and between cell types. Integrating scRNA-seq and seqFISH data enabled the genome-wide imputation of expression, thus generating a complete quantitative and spatially resolved map of gene expression at single-cell resolution across the entire embryo. To illustrate the power of this resource, we used these imputed data to perform a virtual dissection of the midbrain and hindbrain region of the embryo, uncovering spatially resolved patterns of expression associated with both the dorsal–ventral and rostral–caudal axes. Finally, by integrating a second independent scRNA-seq dataset that characterized cell types within the developing gut tube^2^, we resolved the position of two clusters of cells that were both previously assigned a lung precursor identity using the scRNA-seq data^2^. Our spatial data revealed that these two clusters were exclusively located on either the dorsal or ventral side of the gut tube, with corresponding transcriptional differences indicating that the dorsal cells give rise to the esophagus, while the ventral cells give rise to the lung and trachea.

## Results

### Single-cell spatial expression of mouse organogenesis

**Fig. 1:**
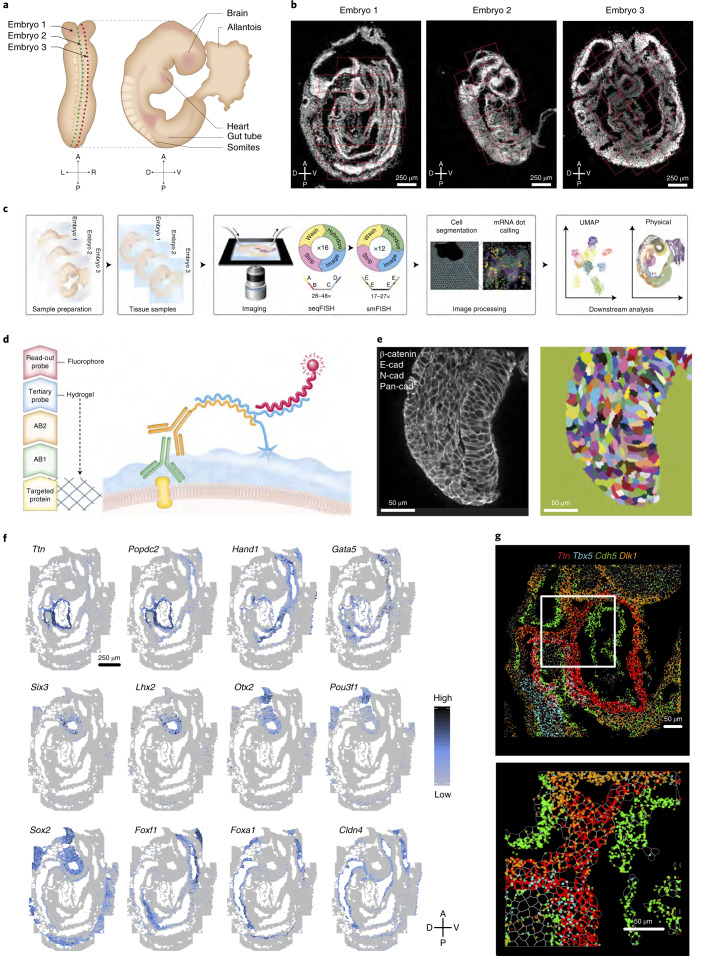
Single-cell spatial transcriptomics map of mouse organogenesis using seqFISH. **a**, Illustration of 8–12 ss mouse embryo. Dotted lines indicate the estimated position of the sagittal tissue section shown in **b**; D, dorsal; V, ventral; R, right; L, left; A, anterior; P, posterior. **b**, Tile scan of a 20-µm sagittal section of three independently sampled 8–12 ss embryos stained with nuclear dye DAPI (white). Red boxes indicate the selected field of view (FOV) imaged using seqFISH. **c**, Illustration of the experimental overview for spatial transcriptomics using seqFISH for 351 selected genes in 16 sequential rounds of hybridization and 12 non-barcoded sequential smFISH hybridization rounds for 36 genes. For each targeted gene, 17–48 unique probes were used to capture the mRNA; UMAP, uniform manifold approximation and projection. **d**, Cell segmentation strategy using a combination of E-cadherin (E-cad), N-cadherin (N-cad), pan-cadherin (Pan-cad) and β-catenin antibody (AB; green) staining detected by an oligo-conjugated anti-mouse IgG secondary antibody (orange) that gets recognized by a tertiary probe sequence. The acrydite group (blue star) of the tertiary probe (blue) gets crosslinked into a hydrogel scaffold and stays in place even after protein removal during tissue clearing. The cell segmentation labeling can be read by a fluorophore-conjugated readout probe (red); AB1, antibody 1; AB2, antibody 2. **e**, Cell segmentation staining of a 10-µm thick transverse section of an E8.5 mouse embryo using the strategy introduced in **d**. Cell segmentation signal was used to generate a cell segmentation mask using Ilastik (right). This was repeated independently for all *N* = 3 embryos with similar results. **f**, Representative visualization of normalized log expression counts of 12 selected genes measured by seqFISH to validate performance. This experiment was repeated independently for all *N* = 3 embryos with similar results. **g**, Highly resolved ‘digital in situ*’* of the cardiomyocyte marker titin (*Ttn*), *Tbx5*, *Cdh5* and *Dlk1*, colored in red, cyan, green and orange, respectively. Dots represent individually detected mRNA spots, and the box represents an area that was magnified for better visualization. This experiment was repeated independently for all *N* = 3 embryos with similar results.

**Extended Data Fig. 1:**
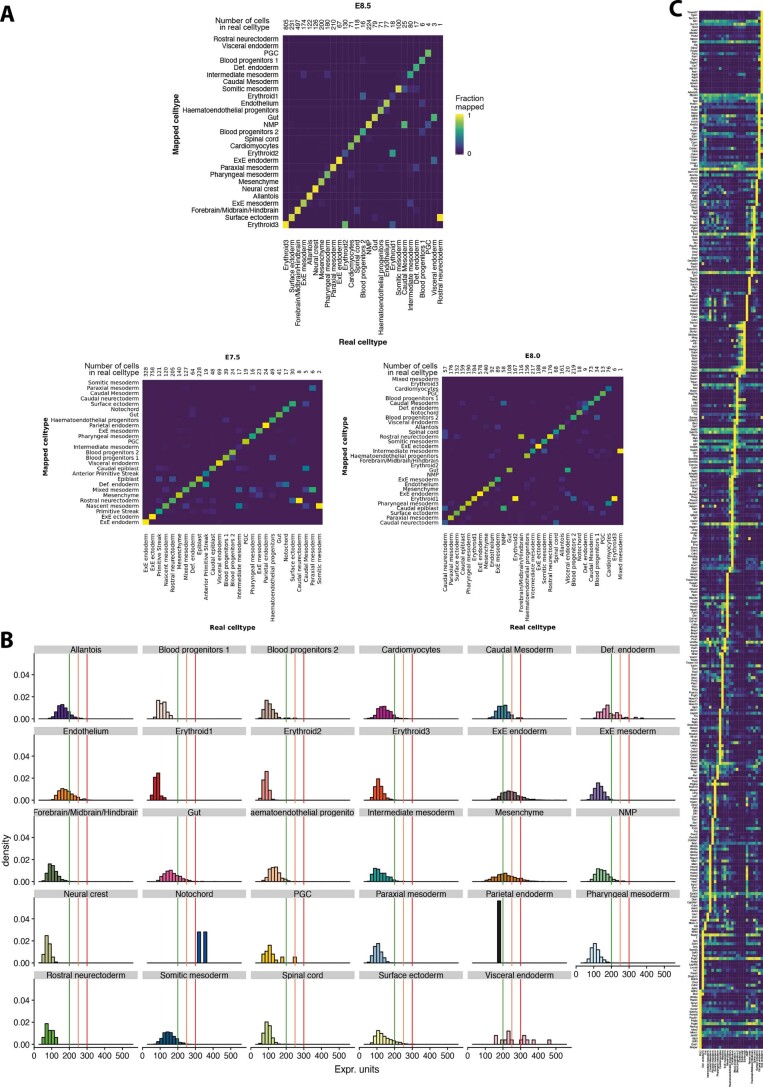
seqFISH probe library design. (**a**) Predicting Gastrulation atlas cell types using the seqFISH probe library for embryonic timepoints E7.5, E8.0, and E8.5. The x-axis is the true cell type of each cell, and the y-axis the mapped cell type. Shading indicates the fraction of cells of each true cell type mapped to each possible cell type. Numbers for each column correspond to the number of cells in each true cell type. (**b**) Histogram, showing the seqFISH library feasibility. Histograms of expression units of the seqFISH probe library genes for each cell type in the E8.5 Gastrulation atlas. Green, orange, and red lines correspond to 200, 250, and 300 normalized expression units respectively, reflecting the guided expression to avoid oversaturation. (**c**) Heatmap showing the mean expression of all selected seqFISH library genes (rows) for each cell type (columns) in the E8.5 Gastrulation atlas.

We performed seqFISH^10,11^ on sagittal sections from three mouse embryos at the 8–12 ss, corresponding to embryonic day (E)8.5–8.75 (Fig. 1a–c). The sections analyzed were chosen to correspond as closely as possible to the midline of the embryo, albeit some variation along the left–right axis could be observed due to embryo tilt (Fig. 1b). Notably, we observed in embryo 2 considerable tilt of the tail region, suggesting depletion of mesodermal and tail-specific populations. In each section, we probed the expression of 351 barcoded genes specifically chosen to distinguish distinct cell types at these developmental stages (Extended Data Fig. 1 and Supplementary Tables 1 and 2). To do this, we exploited a recently published single-cell molecular map of mouse gastrulation and early organogenesis^6^ and determined computationally a set of lowly expressed to moderately expressed genes that were best able to recover the cell-type identities (Methods and Extended Data Fig. 1). Lowly expressed to moderately expressed genes were selected because low overall expression of the library is needed to reduce the optical density of detected transcripts in a cell so that crowding does not prevent single mRNA spots from being resolved reliably.

**Extended Data Fig. 2:**
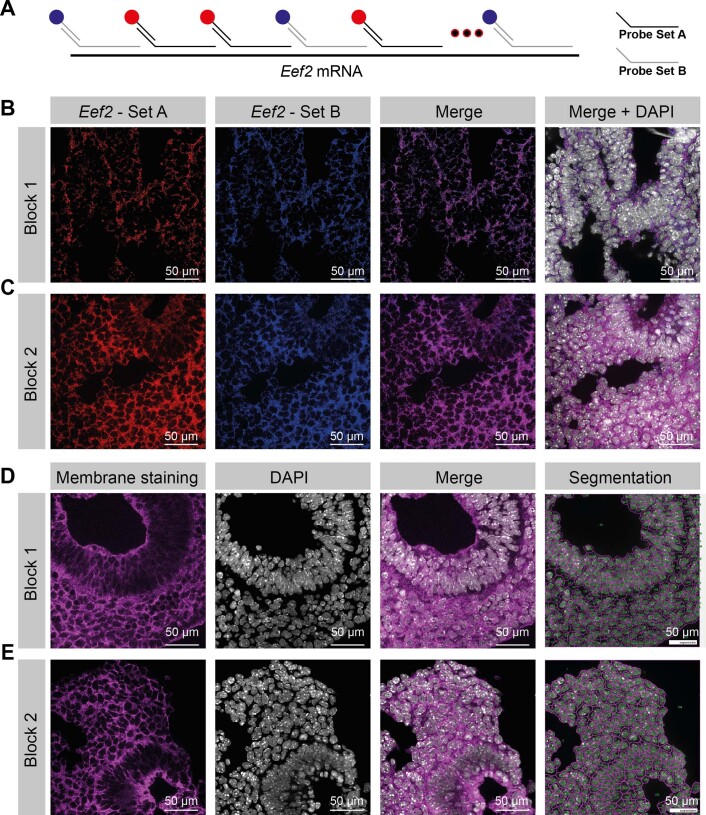
Validation of RNA quality and cell segmentation. Images are representative and were repeated independently for all N = 3 embryos with similar results. (**a**) Schematic overview of the hybridization of two interspersed *Eef2* probe sets to test for RNA integrity. (**b**) Image showing the expression of *Eef2* probe set A (Alexa Fluor 647 - red) and *Eef2* probe set B (Cy3B - blue) for experimental block 1. Color merge of these two images indicates a high degree of overlap between red and blue probes. Merge and DAPI (grey) show overlap of *Eef2* signal surrounding regions where cell nuclei are present. (**c**) Expression profile of *Eef2* probe set A and B, as described in (B) for experimental block 2. (**d**) Image of cell membrane labeling (purple) using a combination of E-cadherin, N-cadherin, Pan-cadherin and β-catenin primary antibody staining, following an optimized cell segmentation protocol (Methods) and nuclear staining using DAPI (grey) for the first tissue section, containing embryo 1 and 2. Signal membrane labeling was used for cell segmentation using Ilastik^35^. (**e**) Cell membrane labeling (purple) and cell segmentation, as described in (D) for experimental block 2.

To obtain a good signal-to-noise ratio for the mRNA spots, we performed tissue clearing to reduce the tissue background signal, as introduced before^25,33^. Briefly, the tissue sections were embedded into a hydrogel scaffold, RNA molecules were crosslinked into the hydrogel and lipid and protein were removed to achieve optimal tissue transparency for seqFISH (Methods). One consequence of depleting proteins is that delineating the cell membrane, and hence cell segmentation, becomes challenging. To address this, before tissue embedding, we performed immunodetection for selected surface antigens, pan-cadherin, N-cadherin, β-catenin and E-cadherin, which could in turn be recognized by a secondary antibody conjugated to a unique DNA sequence. We then hybridized a tertiary probe to the DNA sequence of the secondary antibody, which had a unique smFISH readout sequence and an acrydite group. The acrydite group becomes crosslinked into the hydrogel scaffold and remains in position, even after protein degradation^34^. The unique smFISH readout sequence can subsequently be hybridized with a readout probe conjugated to a fluorophore, allowing the cell membrane to be visualized (Fig. 1d) and enabling segmentation using the interactive learning and cell segmentation tool Ilastik^35^. To validate this strategy, we applied it to a 10-µm thick transverse section of an E8.5 mouse embryo, which confirmed labeling of the cell membrane (Fig. 1e and Extended Data Fig. 2). Before imaging samples for seqFISH, overall RNA integrity was examined by ensuring colocalization of two *Eef2* probe sets, each detected by a unique readout probe conjugated to a different fluorophore (Extended Data Fig. 2 and Supplementary Tables 1 and 3).

Following imaging, the resulting data were segmented as detailed above, and individual mRNA molecules were detected by decoding barcodes over the multiple rounds of imaging. To guarantee high sample quality, the first round of hybridization was repeated following all intervening hybridization rounds, allowing for consistency of mRNA signal intensity to be assessed (Supplementary Fig. 1). In total, following cell-level quality control, we identified 57,536 cells across three embryos with a combined total of 11,004,298 individual mRNA molecules detected. In the embryo tissue sections, each cell contained an average of 196 ± 19.3 (mean ± s.e.) mRNA transcripts from 93.2 ± 6.6 (mean ± s.e.) genes (Supplementary Fig. 2), corresponding to an average of 26.6% of all genes profiled. The set of genes expressed was not biased toward a specific germ layer, and an average of 21.0% ± 1.1% (mean ± s.e.) of genes most associated with a mesoderm identity in the E8.5 Gastrulation atlas was expressed per seqFISH cell, 25.9% ± 2.1% of genes were associated with the endoderm, 28.6% ± 1.3% of genes were identified as extraembryonic and 31.6% ± 3.3% (mean ± s.e.) of genes were associated with the ectoderm.

Next, to confirm the quality of our data, we examined the expression of 12 genes (Fig. 1f) with well-characterized expression patterns. As expected, the cardiomyocyte markers *Ttn*^36^ and *Popdc2* (ref. ^37^) showed the highest expression in the region of the developing heart tube, while *Hand1* (refs. ^38,39^) and *Gata5* (ref. ^40^) showed expression in the heart as well as the more posterior lateral plate mesoderm. Similarly, the expression of four known brain markers, *Six3* (ref. ^41^), *Lhx2* (ref. ^42^), *Otx2* (refs. ^43–45^) and *Pou3f1* (ref. ^46^) was strongest in the developing brain. Turning to genes that mark broader territories, the neural tube marker *Sox2* showed strong expression in the brain and along the dorsal side of the embryo^47,48^. Additionally, expression of the mesoderm marker *Foxf1* was localized to mesodermal cells outlining the developing gut tube, the lateral plate mesoderm and the extraembryonic mesoderm of the allantois^49^. Lastly, two gut endoderm markers *Foxa1* (ref. ^50^) and *Cldn4* (refs. ^51,52^) marked the developing gut tube along the anterior–posterior axis of the embryo. The tissue-specific expression profile of these genes was consistent with both the Gastrulation atlas^6^ (Supplementary Fig. 2) as well as the broad expression territories defined in the EMAGE database^20^. As a further confirmation of the quality of our data, we confirmed the positional expression profiles of the measured Hox gene family members, which followed the described ‘Hox code’ along the anterior–posterior axis^53,54^ (Supplementary Fig. 3). Finally, the high-resolution of seqFISH allows for visualization of mRNA molecules at subcellular resolution, enabling the generation of high-quality digital in situ images (Fig. 1g). Taken together, these analyses demonstrate that we can reliably record the expression profiles of hundreds of genes across an entire embryo cross-section at single-cell resolution.

### Cell-type identity and spatial transcriptional heterogeneity

**Fig. 2:**
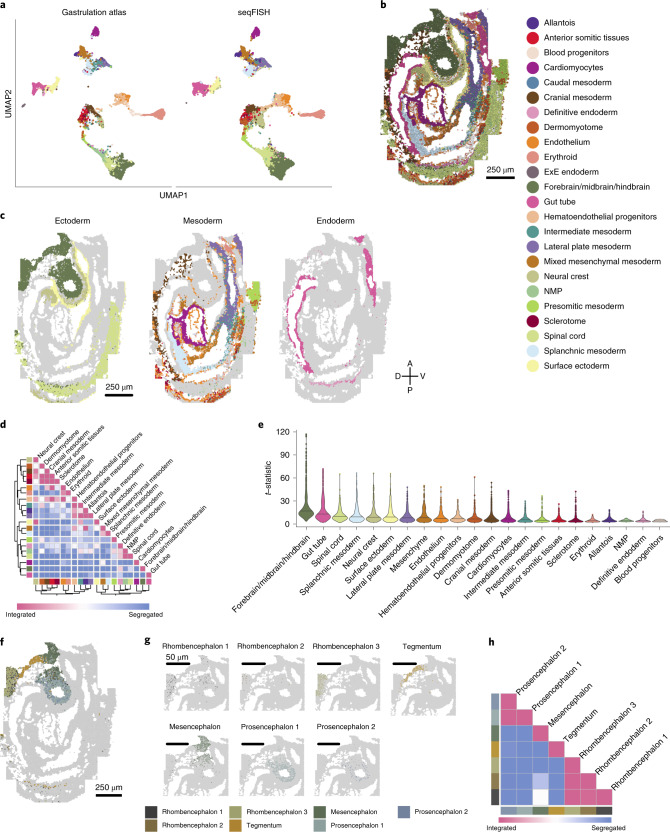
Cell-type annotation and neighborhood characterization. **a**, Projection of seqFISH spatial and Gastrulation atlas cells in joint reduced dimensional space to annotate seqFISH cells based on their nearest neighbors in the mouse Gastrulation atlas. **b**, Real position of annotated seqFISH cells in an embryo tissue section. Colors represent refined cell-type classification; ExE endoderm, extraembryonic endoderm; NMP, neuromesodermal progenitor. **c**, Cell-type maps separated by the three germ layers (ectoderm, mesoderm and endoderm). **d**, Cell–cell contact map displaying the relative enrichment toward integration and segregation of pairs of cell types in space. Cell types are clustered by their relative integration with others. **e**, Violin plots showing the *t*-statistic for each gene and cell type corresponding to a measure of the degree of residual transcriptional heterogeneity explained by space. **f**, Reclustering of forebrain/midbrain/hindbrain cell types into seven spatially distinct clusters. **g**, Zoom in of the brain region to visualize four major brain regions and seven subclusters identified in **f**. **h**, Cell–cell contact map of brain subclusters in space, ordered roughly anatomically from hindbrain to forebrain.

**Extended Data Fig. 3:**
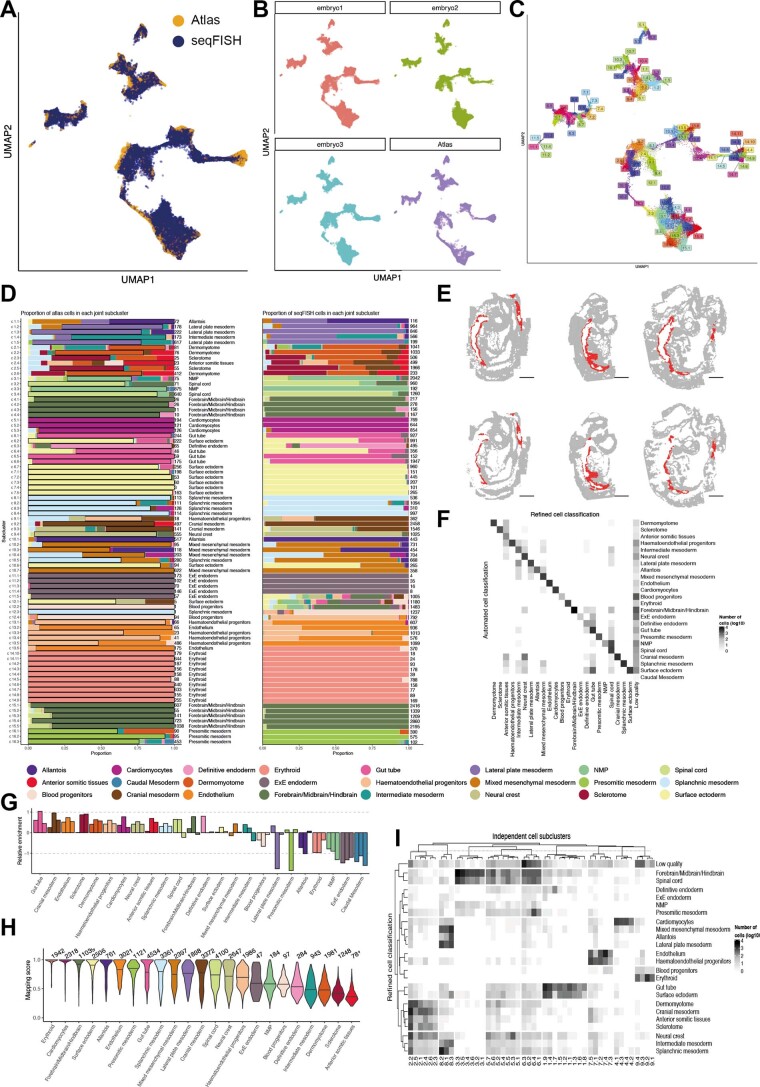
Optimizing cell type annotation. (**a**) Joint UMAP of Gastrulation atlas and seqFISH expression data, with cells colored by data modality. (**b**) Joint UMAP of Gastrulation atlas and seqFISH expression data, with panels corresponding to each embryo and the Gastrulation atlas dataset. (**c**) Joint UMAP of Gastrulation atlas and seqFISH expression data, colored by joint subclustering with labels corresponding to centroid in UMAP coordinates. (**d**) Barplots of the proportion of cell types from the Gastrulation atlas cells present in each subcluster (left), and automated cell type classification for seqFISH data (right). Numbers beside each bar correspond to the number of cells, and labels beside the left barplot correspond to the majority cell type of the Gastrulation atlas cells for each joint subcluster. (**e**) Spatial map of virtual dissection of cells to be classed as developing gut tube, for each embryo (columns) and z-slice (rows). Scale bar 250 µm. (**f**) Heatmap of contingency table of automated cell type label for seqFISH cells (rows) and refined cell type classification (columns). (**g**) Barplot of relative enrichment in abundance of seqFISH cells compared to Gastrulation atlas cells, each bar corresponds to embryo 1, 2, and 3, from left to right. (**h**) Violin plots of automated cell type mapping score for each seqFISH cell, with bar corresponding to median. Numbers above correspond to the number of cells classed into each cell type. (**i**) Heatmap of contingency table of cell type label for seqFISH cells (rows) and independent unsupervised cell subclusters (columns).

Thus far, we have focused on the expression of individual genes. However, the real power of the data derives from the ability to study coexpression of hundreds of genes within their spatial context. To develop this potential, as a first step, we assigned each cell within the seqFISH-profiled embryos a distinct cell-type identity using cell-type mapping. To make this assignment, we integrated each cell’s expression profile from seqFISH with the E8.5 cells from the Gastrulation atlas^6^ using batch-aware dimension reduction and mutual nearest neighbors (MNN) batch correction^55^ (Extended Data Fig. 3) before annotating seqFISH cells based on their nearest neighbors in the Gastrulation atlas (Fig. 2a and Extended Data Fig. 3). We further manually refined this automated cell-type classification using a cell type’s anatomical location and by performing joint clustering of both datasets and comparing their relative cell-type contribution and gene expression profiles (Extended Data Fig. 3 and Methods). The assigned cell-type identities were consistent with known anatomy as well as with the expression of distinct marker genes (Figs. 1f and 2b,c and Supplementary Figs. 4–6).

**Extended Data Fig. 4:**
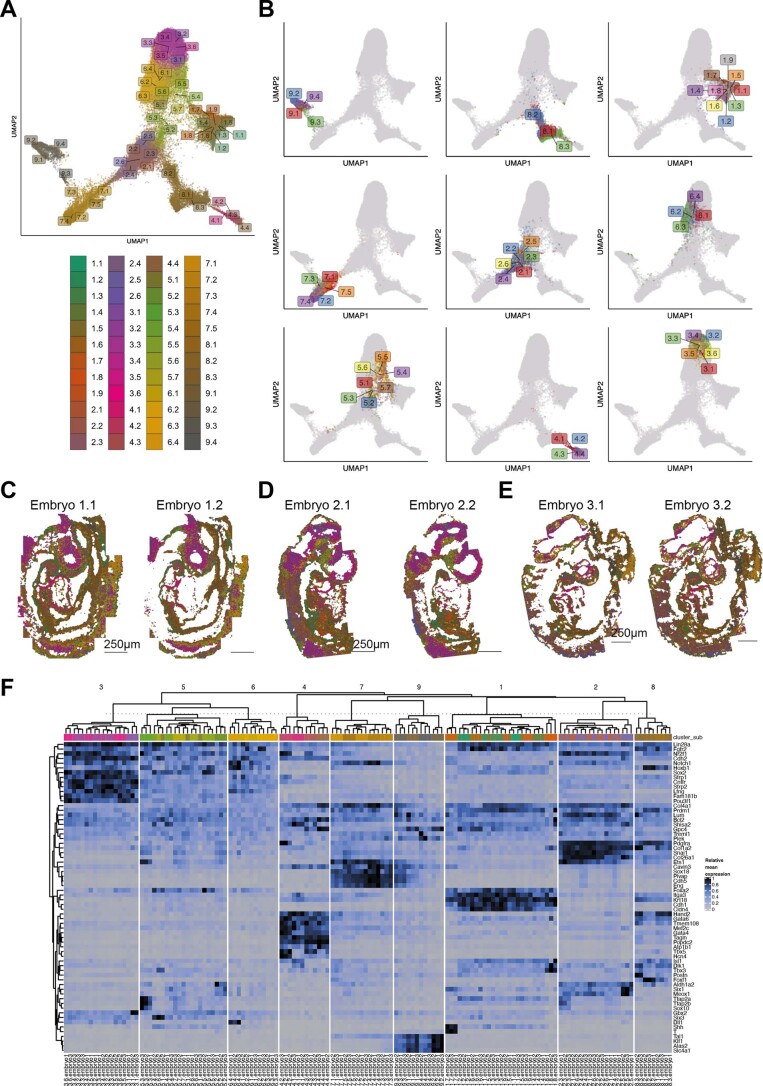
Unsupervised clustering of seqFISH data. (**a**) UMAP of seqFISH expression data, with cells colored by unsupervised subclusters, with labels corresponding to centroid in UMAP coordinates. (**b**) Multiple panels displaying UMAP of seqFISH expression data, with cells for each separate cluster colored by the associated subcluster, with labels corresponding to centroid in UMAP coordinates. (**c**) Spatial map of embryo 1 cells colored by unsupervised subclusters (colors matching panel A) for each z-slice. Scale bar 250 µm. (**d**) as in C with embryo 2. (**e**) as in C with embryo 3. (**f**) Heatmap of relative mean expression of seqFISH cells grouped by embryo and unsupervised subcluster (columns) for genes selected as appearing in the top three significant marker genes (rows) for any of the subclusters. Colors along the top correspond to unsupervised subclusters with legend matching panel A.

As an alternative, we performed direct clustering of the seqFISH data, which revealed similar groupings of cells (Extended Data Fig. 4), indicating that a small number of carefully chosen genes can provide enough information to accurately group cells. However, we note that assigning cell-type identity using only a small number of marker genes is likely to be less reliable than inferring identity through reference to the Gastrulation atlas. Indeed, upon performing a further simulation on randomly selected subsets of the seqFISH gene panel, we observed decreasing cell-type recovery accuracy, more so for the imaging data than for the Gastrulation atlas or even for independent wild-type (WT) chimera control scRNA-seq cells (Methods and Supplementary Fig. 7), suggesting that it may be prudent to select more cell-type marker genes than would be suggested by computational analysis of scRNA-seq data.

**Extended Data Fig. 5:**
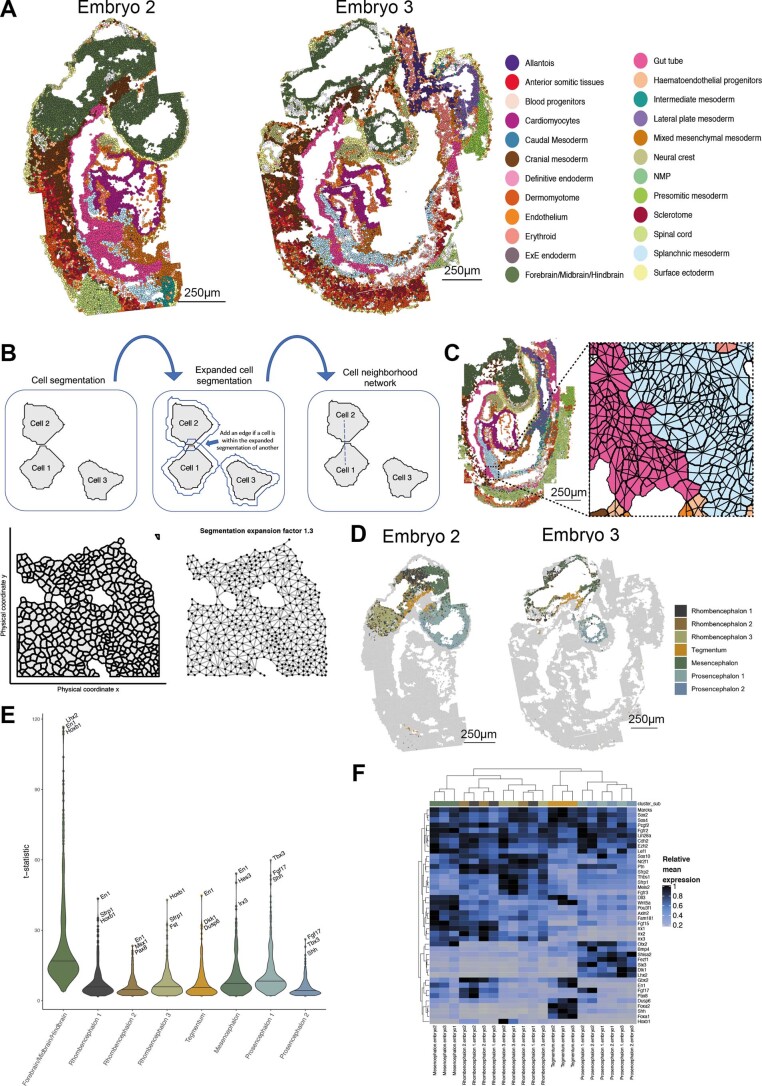
Cell annotation and constructing the cell-contact map. (**a**) Spatial map of embryos 2 and 3, colored by refined cell type. Scale bar 250 µm. (**b**) Schematic of construction of cell neighborhood network, where cell segmentation polygons are expanded and a network edge drawn if another cell is within the expanded polygon region. Below is the resulting network for a selected field of view (Position 0, Embryo 1). (**c**) Visualization of cell neighborhood network using spatial map of embryo 1 with zoom in to reveal cell neighborhood network edges among cells. Scale bar 250 µm. (**d**) Spatial maps of embryos 2 and 3, with cells colored by brain subtypes, and other cells in grey. Scale bar 250 µm. (**e**) Violin plot showing t-statistic corresponding to spatial heterogeneity test for each gene within brain subtype. The top three genes are labeled for each violin, and the bar corresponds to the median. (**f**) Heatmap of relative mean expression of each embryo and brain subcluster for significant (one-sided two-sample t-test FDR-adjusted P-value < 0.05, absolute LFC > 0.2) marker genes.

Next, to study when boundaries between emerging tissue compartments are established in the developing embryo, we statistically quantified whether cells assigned to the same type were spatially coherent within the embryo and determined the extent to which pairs of cell types were colocated (Fig. 2d,e and Methods). We used a permutation strategy to evaluate the relative enrichment or depletion of direct cell–cell contact events between each cell type resulting in a cell–cell contact map (Fig. 2d and Extended Data Fig. 5). Certain cell types, such as cardiomyocytes and the gut tube, were spatially and morphologically distinct, while others, like the endothelium, were interspersed and spread across the entire embryo space.

**Extended Data Fig. 6:**
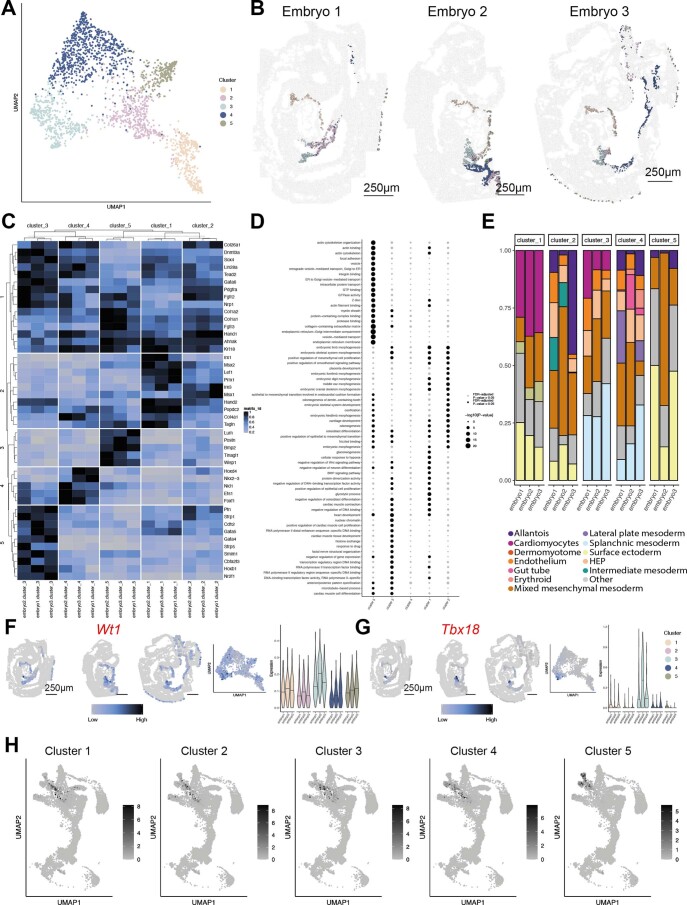
Characterization of mixed mesenchymal mesoderm cluster. (**a**) UMAP embedding of mixed mesenchymal mesoderm seqFISH cells, colored by unsupervised clusters. (**b**) Spatial plots with cells colored by mixed mesenchymal mesoderm unsupervised clusters. (**c**) Heatmap of mean expression of each embryo and mixed mesenchymal mesoderm cluster for significant (FDR-adjusted P-value < 0.05, absolute LFC > 0.2) marker genes. (**d**) Dotplot of significantly enriched gene ontology terms for each mixed mesenchymal mesoderm cluster (Fisher’s Exact Test, FDR-adjusted P-value < 0.05). (**e**) Proportional bar plot showing the corresponding cell types for spatial neighbors of each embryo and mixed mesenchymal mesoderm cluster, with cell types with a small percentage grouped into Other cell types. Abbreviation used: HEP = hematoendothelial progenitors. (**f**) Spatial plots of inferred *Wt1* expression among mixed mesenchymal mesoderm clusters, UMAP embedding of cells colored by Wt1 expression, and violin plot of Wt1 expression for each embryo and mixed mesenchymal mesoderm cluster. (**g**) As for (**f**) for inferred expression of *Tbx18*. (**h**) Scatterplot of UMAP embedding of E8.5 Gastrulation atlas cells, colored by proportion of selection within nearest neighbor set for each mixed mesenchymal mesoderm cluster.

More generally, while most cell types are characterized using prior knowledge of expression markers and lineage inference, other populations, such as the mixed mesenchymal mesoderm, represent a cell state rather than a defined cell type. Mesenchyme represents a state in which cells express markers characteristic of migratory cells loosely dispersed within an extracellular matrix^56^. This strong overriding transcriptional signature of mesenchyme, irrespective of location, makes it challenging to distinguish which cell types this mixed mesenchymal mesoderm population represents using classical scRNA-seq data. By contrast, our integrated spatial expression map allowed us to resolve five transcriptionally distinct subpopulations (clusters 1–5) that were spatially defined (Extended Data Fig. 6 and Methods).

Based on its anatomical position overlaying the developing heart, we infer that cluster 1 reflects cells with a cardiac mesoderm and pericardium identity. Clusters 2 and 3 are located in the septum transversum, in the region of the forming hepatic plate and proepicardium. At this developmental stage, bone morphogenetic protein (BMP) signaling from the developing heart and fibroblast growth factor (FGF) signaling from the septum transversum mesenchyme are critical for the induction of hepatic fate specification in the foregut^57,58^. Consistent with this, we observed enrichment for BMP signaling in cluster 1 (Extended Data Fig. 6). Additionally, in cluster 3, we observed the coexpression of proepicardial markers *Tbx18* and *Wt1* (refs. ^59,60^) whose deletion results in heart^61^ and liver^62^ defects (Extended Data Fig. 6). Our ability to spatially map cluster 3 revealed its position caudal to the forming heart, corresponding with the known location of the proepicardium. Together, their location and expression profiles indicate that the cells from clusters 2 and 3 will contribute to the hepatic mesenchyme (important for hepatoblast specification) and the proepicardium, respectively. Lastly, clusters 4 and 5 are located toward the body wall, suggesting a somatic mesoderm identity that will contribute to the dermis^63^.

To characterize additional spatially driven transcriptional heterogeneity, we used a linear model to identify genes that show a strong spatial expression pattern within each cell type (Fig. 2e, Supplementary Table 4 and Methods). This indicated that residual transcriptional heterogeneity in the forebrain/midbrain/hindbrain cluster can be explained by localized patterns of expression, most likely resulting from the presence of regionally specific developing brain subtypes (Supplementary Table 5). To investigate this, we performed a focused reclustering of forebrain/midbrain/hindbrain cells, recovering four major brain subregions and seven subclusters (Fig. 2f,g). Cross-referencing spatial location and underlying gene expression signatures allowed us to identify subclusters associated with the prosencephalon, mesencephalon, rhombencephalon and the tegmentum (Fig. 2g,h and Extended Data Fig. 5).

### A 10,000-plex spatial map of inferred gene expression

By design, our seqFISH library allowed us to probe the expression of specific genes associated with cell-type identity. Additionally, we directly measured the expression of a number of genes associated with key signaling cascades, for example, Notch^64^ and Wnt^65^. Nevertheless, a full, unbiased view of the interplay between a cell’s spatial location and its molecular profile and how this influences development would benefit from measuring expression of the entire transcriptome, which is not straightforward with existing highly multiplexed RNA FISH protocols.

**Fig. 3:**
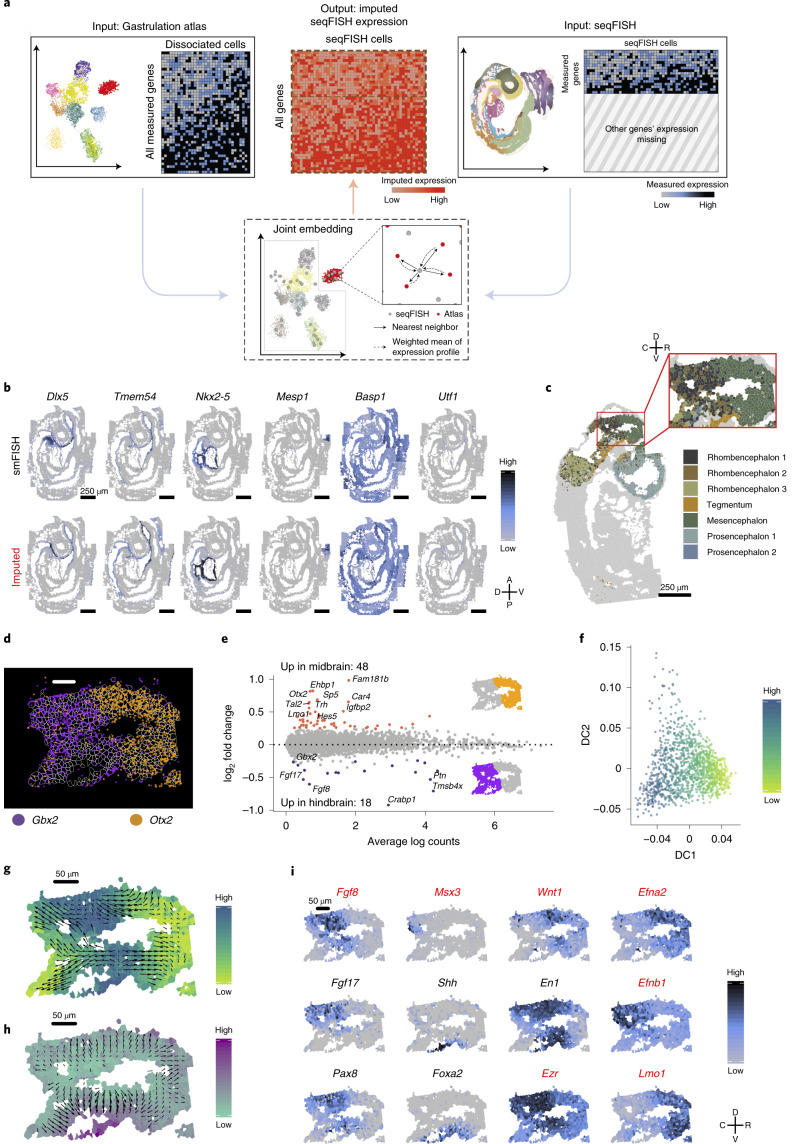
Creating and using a 10,000-plex spatial map. **a**, Schematic representation of the imputation strategy. **b**, Independent validation of imputation performance by comparing normalized gene expression profiles of selected genes measured by smFISH with the corresponding imputed gene expression profiles. **c**, Visualization of brain subclusters in embryo 2 and virtual dissection of the MHB, highlighted by the red rectangle and inset zoom; C, caudal; R, rostral; D, dorsal; V, ventral. **d**, ‘Digital in situ’ showing detected mRNA molecules of a mesencephalon and prosencephalon marker *Otx2* (orange dots) and a rhombencephalon marker *Gbx2* (purple dots) to identify the MHB; scale bar, 50 µm. **e**, MA (log ratio and mean average) plot showing differential gene expression analysis using a two-sample *t*-test between the virtually dissected hindbrain region (orange; 48 genes significantly upregulated; absolute LFC > 0.2, FDR-adjusted *P* value of <0.05) and virtually dissected midbrain region (purple; 18 genes significantly upregulated; absolute LFC > 0.2, FDR-adjusted *P* value of <0.05) using the imputed transcriptome. **f**, Diffusion pseudotime analysis of the virtually dissected region to understand the dynamics of gene expression at the MHB. The scatter plot of diffusion-based embedding of virtually dissected cells displays diffusion components (DC) 1 and 2. Cell colors correspond to inferred diffusion pseudotime. **g**, Spatial graph showing virtually dissected cells colored by inferred diffusion pseudotime dominated by DC1. Arrow sizes correspond to the magnitude of change of the pseudotime value within the region in the direction from large to small pseudotime values. The highest pseudotime values are observed along the MHB region, smoothly diffusing outward to the midbrain and hindbrain regions. **h**, Spatial graph showing virtually dissected cells colored by DC2. Arrow sizes correspond to the magnitude of change of the DC2 value within the region. The most extreme DC2 values are observed perpendicular to the MHB region, smoothly diffusing outward to the floor plate and roof plate regions. **i**, Visualization of normalized log expression counts of important regulators of midbrain/hindbrain formation. Gene names shown in red font indicate imputed expression, while gene names shown in black font indicate measured expression.

**Extended Data Fig. 7:**
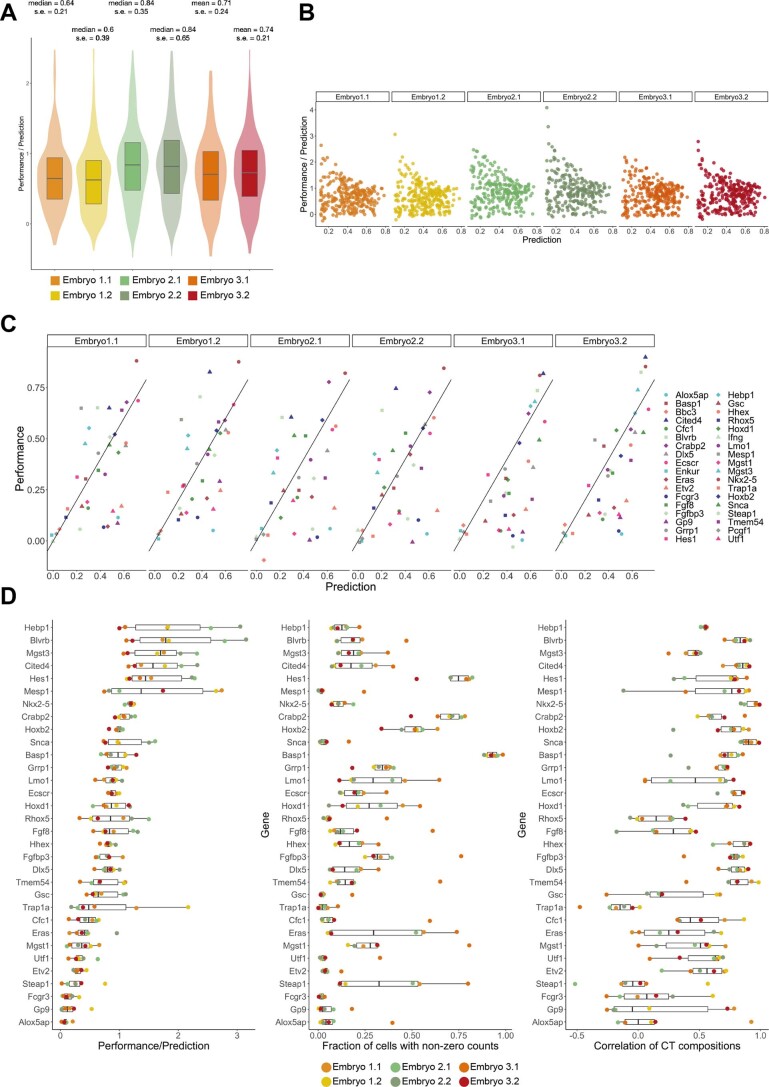
Imputation strategy. (**a**) Normalized performance as a validation of imputation. Violin plots show distributions (across measured genes) of normalized performance for each embryo and z-slice. Median and standard error appear above each violin. (**b**) Scatterplots of prediction scores (x-axis) and normalized performance scores (y-axis). Genes with prediction score lower than 0.1 show stochastic deviations in normalized performance and were filtered. (**c**) Scatterplots of performance and prediction scores for genes probed by smFISH, with each panel corresponding to one embryo and z-slice, and points corresponding to genes. Genes exhibiting strong field of view effect (FOV: 39, 40, 44) were discarded from quantification of performance and prediction scores. (**d**) Assessment of quality of imputation for smFISH genes. Genes are ordered according to the median Performance/Prediction ratio across all embryos and z-slices. Left panel: Boxplots representing Performance/prediction (x-axis) for genes profiled in smFISH across all embryos and z-slices. Middle panel: Boxplots representing fraction of cells with non-zero smFISH counts for the corresponding genes. Right panel: Boxplots representing correlation (across cell types) between fraction of cells (out of all cells for the corresponding cell type) with non-zero smFISH counts for the corresponding genes and fraction of cells with non-zero logcounts in the Gastrulation Atlas. Individual data points are overlaid on each boxplot. N = 6 technical samples across 3 biologically independent embryos. Boxes display 25^th^, 50^th^, and 75^th^ percentiles, and whiskers extend to closest observation within outlier range, defined as not more than 1.5 times the interquartile range.

To overcome these limitations, we built upon the MNN mapping approach (Fig. 2 and Extended Data Fig. 3) and inferred the full transcriptome of each seqFISH cell by considering the weighted expression profile of the cells to which it is most transcriptionally similar to in the Gastrulation atlas (Fig. 3a, Extended Data Fig. 7 and Methods). To test the integrity of this strategy, for each gene probed in our seqFISH experiment (excluding *Xist*, as it is sex specific), we used the remaining 349 measured genes to map all cells to the Gastrulation atlas and imputed the expression of the withheld gene. To evaluate performance, we calculated for each gene and across all cells the Pearson correlation (‘performance score’) between the imputed expression counts and the measured seqFISH expression levels. To estimate an upper bound on the performance score (that is, the maximum correlation we might expect to observe), we exploited the four independent batches of E8.5 cells that were processed in the scRNA-seq Gastrulation atlas. We treated one of the four batches as the query set and used the leave-one-out approach described above to impute the expression of the 350 genes of interest by mapping cells onto a reference composed of the remaining three batches before computing the Pearson correlation between the imputed and true expression counts (‘prediction score’; Methods). Computing the ratio of the performance (seqFISH–scRNA-seq) and prediction (scRNA-seq–scRNA-seq) scores yields a normalized performance score. Across genes, we observed a median normalized performance score of 0.73 (lower quartile, 0.32; upper quartile, 1.09) (Extended Data Fig. 7), suggesting that our ability to infer gene expression is comparable to what might be expected when combining independent scRNA-seq datasets. While we observed a high level of consistency among the independently captured genes, we identified a subset of genes that did not perform as well (Methods). These nine genes were either lowly or rarely expressed in the independent smFISH data or were variably expressed between replicates (Extended Data Fig. 7 and Supplementary Table 6). Consequently, care must be taken in interpreting imputed expression patterns for such genes.

To further validate our imputation strategy, we used non-barcoded sequential smFISH to measure the expression of 36 additional genes in the embryo sections probed by seqFISH and contrasted the true expression profile with the imputed values (Fig. 3b). This independent validation (these smFISH genes were not used in the MNN mapping) confirmed that imputation reliably recovered gene expression profiles (Fig. 3b and Supplementary Figs. 8–12). For example, we observed a strong overlap between measured and imputed expression for *Dlx5* (ref. ^66^), an essential and spatially restricted regulator of craniofacial structures, in the anterior surface ectoderm and first branchial arch. Additionally, we noted that *Tmem54* was inferred to be specifically expressed in the anterior surface ectoderm and along the gut tube, *Nkx2-5* (refs. ^67,68^) was inferred to be expressed in the developing heart, and *Mesp1* was inferred to be expressed in the posterior presomitic mesoderm^69,70^. Finally, the ubiquitous expression profile of *Basp1* and the absence of expression of the germ line marker *Utf1* (ref. ^71^) was also recapitulated in the imputed expression maps.

### Reconstruction of MHB formation

To illustrate the utility of the imputed data, we focused on a well-described developmental process that takes place at this embryonic stage, the formation of the MHB, also known as the isthmus organizer. The MHB acts as a signaling hub that is essential for patterning of the adjacent midbrain and hindbrain regions by inducing two distinct transcriptional programs via defined signaling cascades (reviewed in^72–74^). Thus, the MHB presents an important dividing point in the developing brain, functioning both as a signaling center and as a physical barrier of the developing brain ventricles^75^. We observed expression of the mesencephalon and prosencephalon marker *Otx2* (refs. ^43,76^) and the rhombencephalon marker *Gbx2* (refs. ^76,77^) in the brain region of all three embryos, albeit the sagittal section for embryo 2 appeared to capture this region most comprehensively (Supplementary Fig. 13). Focusing on this region of embryo 2, we used the expression of *Gbx2* and *Otx2* to identify the precise boundary between the two subclusters (Fig. 3c,d). Subsequently, we virtually dissected the *Otx2*-positive midbrain region and the *Gbx2*-positive hindbrain region (Supplementary Fig. 13) and performed a differential expression analysis (using the imputed expression profiles) to identify additional genes that distinguish the two regions (Fig. 3e). This identified 66 genes (false discovery rate (FDR)-adjusted *P* value of <0.05; absolute log fold change (LFC) > 0.2) with spatially distinct expression profiles between the two regions (Supplementary Table 7).

To further understand the spatial distribution of gene expression at the MHB, we investigated whether further local differences in spatial expression patterns were present. Using a diffusion-based transcriptional embedding^78^, we observed smoothness of the estimated diffusion components in physical space, with an extreme corresponding to the MHB itself (Fig. 3f,g and Methods). Using a spatial vector field to capture local magnitude and direction of changes in DC1 in space, we observed an outward radiation of signaling gradients from the MHB region, corresponding to the rostral–caudal axis (Fig. 3g), with strong enrichment for *Lmo1* (ref. ^79^) in the midbrain and *Pax8* (ref. ^80^) in the hindbrain (Fig. 3i). Additionally, we observed that DC2 corresponds to an emerging dorsal–ventral axis (Fig. 3h), demonstrating that the coordinate space of the brain is established at this stage of development.

**Extended Data Fig. 8:**
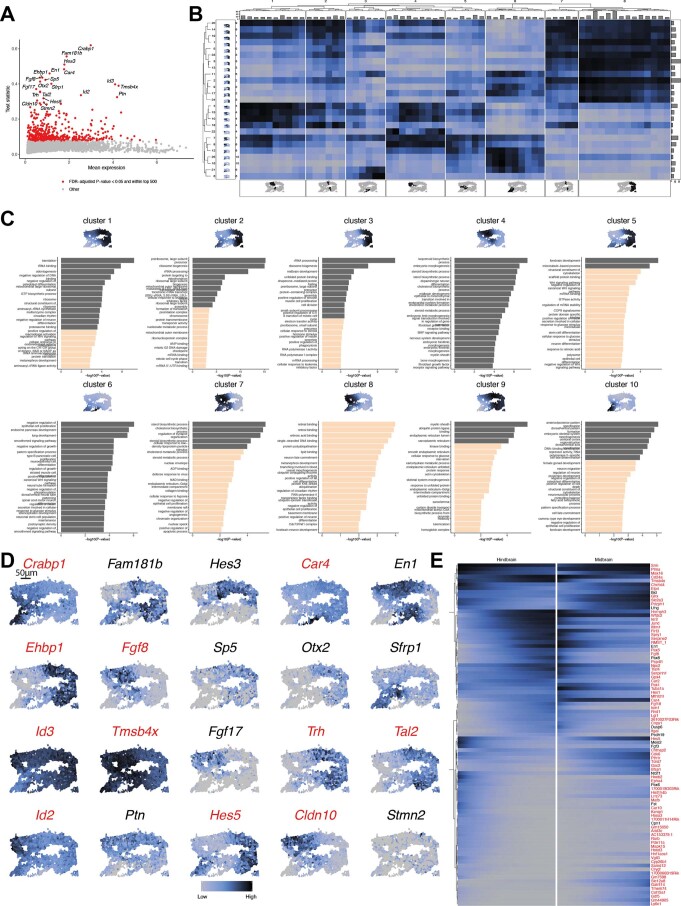
Statistical interrogation of the Midbrain-Hindbrain Region. (**a**) Scatterplot of all imputed genes, showing mean expression (x-axis) and scHOT weighted mean test statistic (y-axis). Significant (scHOT permutation test, FDR-adjusted P-value < 0.05) and top 500-ranked genes are colored red, and the top 20 genes are labeled. (**b**) Heatmap of expression of clustered MHB genes and cells, split along columns by clustered cell regions, and along rows by mean expression profiles. Top barplots display the number of cells within each group, right barplots display the number of genes withing each group, bottom spatial graphs display cells belonging to each split cluster, and left spatial graphs show the mean spatial expression for genes that characterize each split cluster. (**c**) Spatial graph of the MHB with cells colored by mean expression of the genes belonging to each cluster, and barplots displaying the top 20 enriched gene ontology terms with bar length corresponding to -log10(unadjusted P-value), dark grey bars correspond to FDR-adjusted P-value < 0.05. Fisher’s Exact Test was used for gene ontology testing. (**d**) Spatial graphs of the MHB for the top 20 ranked scHOT weighted mean genes, with red titles corresponding to inferred gene expression. (**e**) Smoothed heatmap of cells (columns), ordered along DPT split by anatomical midbrain and hindbrain regions, for genes strongly correlated with DPT (rows). Cells are ordered from low to high DPT from left to right for the hindbrain region, and ordered from high to low DPT from left to right for the midbrain region. Gene names in red correspond to inferred gene expression.

To identify genes contributing toward the emergence of this coordinate space, we performed unbiased detection of spatially variable genes (Methods^81^, Extended Data Fig. 8 and Supplementary Table 8), uncovering distinct spatial expression patterns, especially along the dorsal–ventral axis within the hindbrain. Among spatially variable genes, several are known regulators of cell fate commitment, including *Fgf8*, *Fgf17*, *Wnt1* and *En1*, all of which displayed their highest level of expression at the MHB (Fig. 3i). *Fgf8* is a known MHB organizer whose posterior expression relative to the boundary is necessary for repressing the expression of *Otx2* in the rhombencephalon^82^. Consistent with this, we inferred that the imputed expression of *Fgf8* was negatively correlated with *Otx2*. By contrast, *Wnt1*, whose imputed expression is restricted rostral of the MHB, is known to upregulate *Otx2* expression in the midbrain^83,84^. *En1* (engrailed 1) expression was observed across the MHB with no rostral or caudal bias^85–87^ (Fig. 3i). In *Wnt1*^–/–^ embryos, the expression of *En1* is absent, consistent with the importance of WNT-1 signaling for *En1* expression^88,89^. This is supported by the observation that the deletion of *En1* results in a midbrain–hindbrain deletion, with a phenotype that closely resembles the *Wnt1*^*–/–*^-mutant mice^85^. We also observed spatially distinct expression of *Foxa2* and *Shh* in the floor plate, another important midbrain organizer (Fig. 3i), consistent with the observation that both genes are critical for specification of the floor plate^90^. Additionally, we noted a cluster of cells, characterized by the highly restricted inferred expression of *Msx3*, in the dorsal developing neural tube^91^. Finally, we observed that *Ezr* (ezrin), *Efna2* (ephrin A2) and *Efnb1* (ephrin B1) were among the genes with the most spatially variable patterns of expression. The ephrin signaling pathway is a known regulator of cell sorting and plays an important role in the formation of a sharp MHB that compartmentalizes the brain^92^. Consistent with this, *Efna2* and *Efnb1* are inferred to occupy distinct territories of gene expression on each side of the MHB. Taken together, this analysis demonstrates how the imputed data can be used to reliably recapitulate and enhance our understanding of important developmental processes, such as MHB formation.

### Spatial patterning of cells within the gut tube

**Fig. 4:**
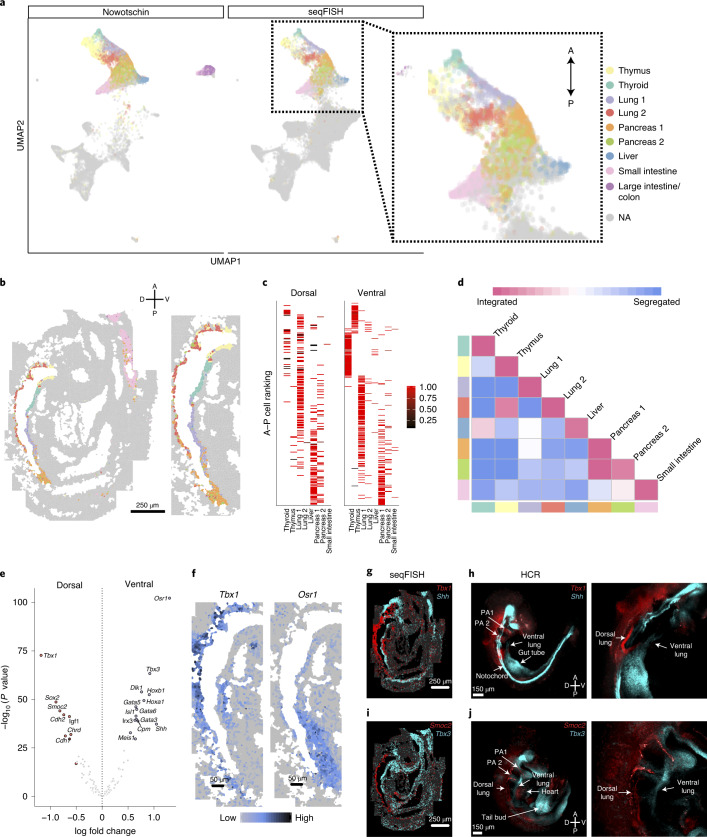
Spatial characterization of gut tube organogenesis. **a**, Joint embedding of seqFISH data and Nowotschin et al. cells corresponding to the developing gut tube^2^ with seqFISH cells annotated by their predicted gut tube subtype. Colors represent gut tube subtypes. The zoomed-in region shows anterior–posterior patterning of the gut endoderm cluster in the UMAP space, indicated by an arrow; NA, not annotated cell. **b**, Position of gut tube cell types in the embryo tissue section. Colors represent cell-type classification. A zoom-in image into the region of the gut tube is shown on the right for better visualization. **c**, Anterior–posterior (A–P) ranking of cells corresponding to each gut tube subtype split into dorsal and ventral regions. The bar color corresponds to the mapping score associated with classification into the subtype. **d**, Cell–cell contact map that displays the relative enrichment toward integration and segregation of pairs of gut tube subtypes in space, ordered along the inferred A–P ordering in Nowotschin et al.^2^. **e**, Volcano plot showing a comparison of gene expression between the (ventral) lung 1 and (dorsal) lung 2 subtypes using seqFISH data. Significantly differentially expressed genes (two-sample *t*-test with an absolute LFC > 0.5 and an FDR-adjusted *P* value of <0.05) are highlighted, and corresponding gene names are indicated. **f**, Visualization of *Tbx1* expression (enriched in the dorsal lung 2 cluster) and *Osr1* expression (enriched in the ventral lung 1 cluster). **g**, ‘Digital in situ’ showing detected mRNA molecules for *Tbx1* (red) and *Shh* (cyan) across the entire embryo tissue section. **h**, Multiplexed mRNA imaging of whole-mount E8.75 mouse embryo using HCR of *Tbx1* (red) and *Shh* (cyan). The zoom in shows region-specific expression in the developing lung region; PA, pharyngeal arch. **i**, ‘Digital in situ’ showing detected mRNA molecules for *Smoc2* (red) and *Tbx3* (cyan) across the entire embryo tissue section. **j**, Multiplexed mRNA imaging of whole-mount E8.75 mouse embryo using HCR of *Smoc2* (red) and *Tbx3* (cyan). The zoom in shows region-specific expression in the developing lung region. Images are representative and were repeated independently on *N* = 2 embryos with similar results.

Finally, we examined the emergence of organ precursor cells along the anterior–posterior axis in the developing gut tube. Recently, Nowotschin et al. inferred the pseudo-spatial ordering of E8.75 (13 ss) gut tube cells along the anterior–posterior axis^2^. However, despite validation of the anterior–posterior patterning using targeted in situ hybridization, the ability to finely determine the boundary between cell types and to precisely demarcate the locations of cell types along complex tissues like the gut tube is challenging when using single-gene in situ stainings. To explore whether our data could shed light on this problem, we performed a joint mapping of the seqFISH data with cells from dissected E8.75 (13 ss) gut tubes that were profiled using scRNA-seq^2^ (Fig. 4a and Supplementary Fig. 14). Incorporating this additional scRNA-seq dataset allowed us to refine the cellular annotations for the developing gut tube and nearby relevant cell types; in particular, it allowed us to associate cells with the organs that they would likely contribute to in the adult animal, including thyroid, thymus, lung, liver, pancreas, small intestine and large intestine/colon. Notably, the seqFISH-profiled embryos, in comparison to the Nowotschin dataset, lack cells associated with the large intestine, likely due to the area of the large intestine not being represented in the tissue sections profiled using seqFISH (Supplementary Fig. 14).

**Extended Data Fig. 9:**
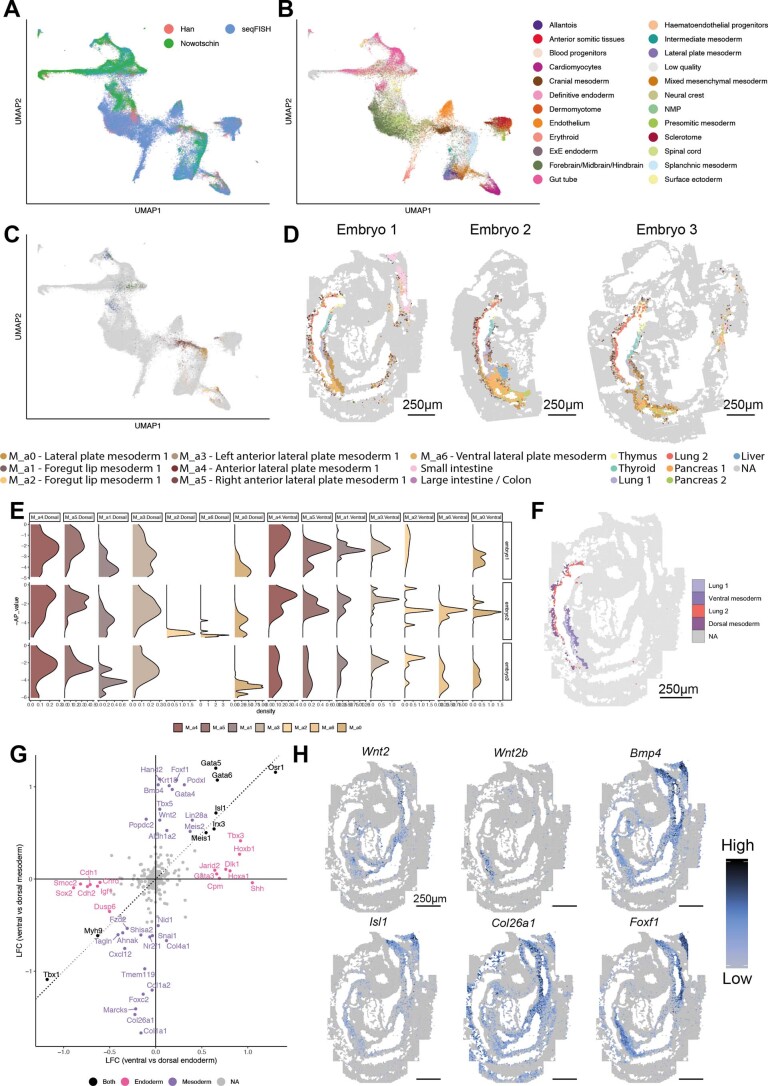
Surrounding mesoderm of the developing gut tube. (**a**) Joint UMAP of Nowotschin *et al*., Han *et al*. and seqFISH expression data, with cells colored by dataset. (**b**) as in (A) with cells colored by corresponding Gastrulation atlas cell type (automatically inferred for cells not coming from the seqFISH dataset). (**c**) as in (A) with cells colored by mesodermal and endodermal subtype for the Han *et al*. dataset, and all other cells colored in grey. (**d**) Spatial graphs of gut tube and surrounding mesodermal cells, colored by inferred gut tube subtype and mesodermal subtypes respectively. (**e**) Density graphs of seqFISH mesodermal cells ordered along physical anterior to posterior axis, split by embryo (rows), and mesoderm cluster and position along dorsal-ventral axis (columns). (**f**) Spatial graph of cells corresponding to gut tube subtypes Lung 1 and Lung 2, as well as surrounding mesodermal cells. (**g**) Scatterplot of log-fold changes corresponding to tests for differential expression between ventral (Lung 1) and dorsal (Lung 2) endodermal cells (x-axis), and ventral and dorsal mesodermal cells (y-axis) for all seqFISH genes. Significant (two-sample t-test, FDR-adjusted P-value < 0.05 and absolute LFC > 0.2) genes are labeled, and colored according to the comparison in which they are selected. (**h**) Spatial graphs of expression of selected genes among those differentially expressed between dorsal and ventral subgroups.

As expected, plotting the physical position of the subclusters showed distinct patterning along the anterior–posterior axis (Fig. 4b). This patterning was mirrored by the presence of spatially distinct populations of cells within the surrounding splanchnic mesoderm (Methods and Extended Data Fig. 9), consistent with recent reports^3^ and supporting the observation that signaling interactions between the gut endoderm and the surrounding mesoderm play key roles in determining cell-type identity^92^.

**Extended Data Fig. 10:**
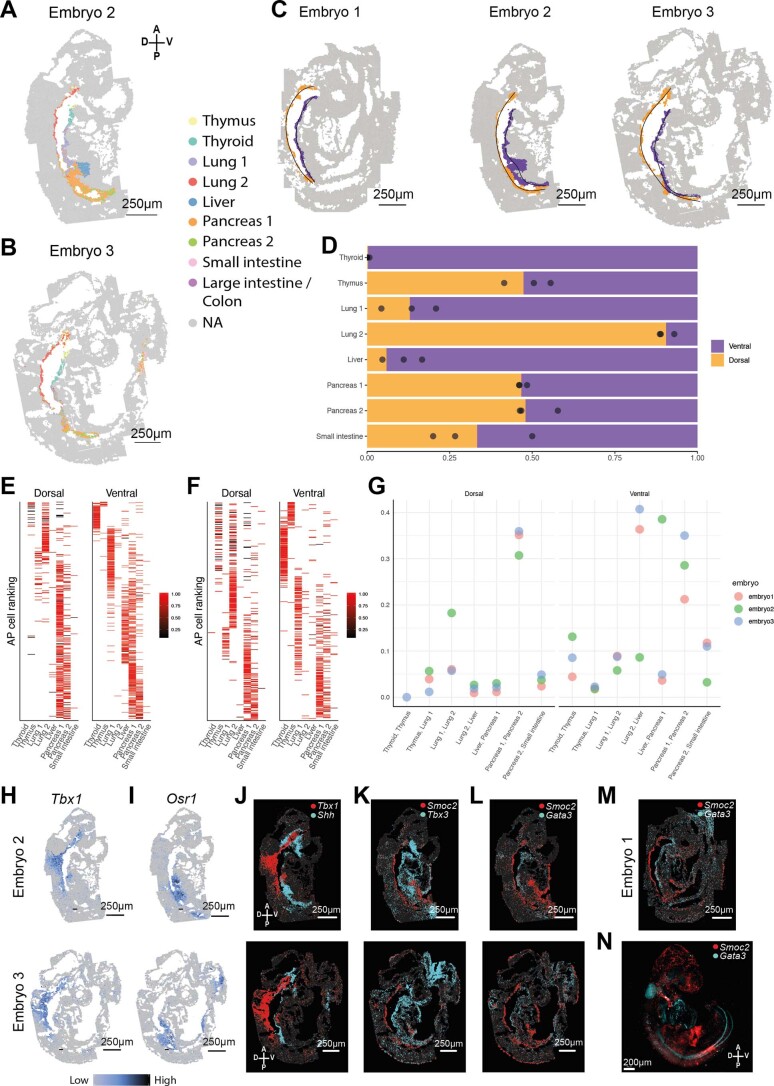
Comparison between dorsal and ventral side of developing gut tube. (**a**) Spatial map of cells corresponding to the developing gut tube for embryo 2. Scale bar 250 µm. (**b**) as in A, for embryo 3. (**c**) Spatial map of anatomical foregut cells for embryos 1, 2, and 3, virtually dissected to correspond to the dorsal (orange) and ventral (purple) regions of the developing gut tube. Black lines correspond to the fitted principal curve model for each embryo and developing gut tube region, where cells are ordered from anterior to posterior using these models. Scale bars 250 µm. (**d**) Barplot showing relative proportion of cells in ventral or dorsal anatomical region of the developing hindgut, split by classification of developing gut tube subtype. Black points correspond to relative proportions for each individual embryo. (**e**) Anterior-posterior ranking of embryo 2 cells, corresponding to each gut tube subtype, split into dorsal and ventral regions. Bar color corresponds to the mapping score associated with classification into the subtype. (**f**) as in E for embryo 3. (**g**) Scatterplot of anterior-posterior logistic regression prediction error rate (y-axis) for each contiguous pair of developing gut tube subtypes (x-axis), split into dorsal and ventral anatomical regions, for each embryo. A higher prediction error rate corresponds to a higher level of relative mixing of subtypes along the anterior-posterior axis, while a lower prediction error rate corresponds to more distinct and separate arrangement of subtypes along the anterior-posterior axis. (**h**) Spatial expression of *Tbx1* only in the developing gut tube for embryos 2 (top) and 3 (bottom). Scale bar 250 µm. (**i**) as in H for gene *Osr1*. (**j**) ‘*Digital in situ’* showing detected mRNA molecules for *Tbx1* (red) and *Shh* (cyan) for embryos 2 (top) and 3 (bottom). Scale bar 250 µm. (**k**) as in J for genes *Smoc2* (red) and *Tbx3* (cyan). (**l**) as in J for genes *Smoc2* (red) and *Gata3* (cyan). (**m**) ‘Digital *in situ*’ showing detected mRNA molecules for *Smoc2* (red) and *Gata3* (cyan) for embryo 1. Scale bar 250 µm. (**n**) Multiplexed mRNA imaging of whole-mount E8.75 mouse embryo using hybridization chain reaction (HCR) of *Smoc2* (red) and *Gata3* (cyan). Image is representative and were repeated independently on N = 2 embryos with similar results.

More unexpectedly, topological cell–cell contact analysis of the gut tube subclusters revealed a spatial separation of two lung subtypes (lung 1 and lung 2) defined by Nowotschin et al. (Fig. 4c,d). Specifically, cells assigned a lung 1 identity were located exclusively on the ventral side of the gut tube, while lung 2 cells were located on the dorsal side (Fig. 4b and Extended Data Fig. 10). It has previously been observed at E9.5 that esophagus progenitors are located on the dorsal side of the gut tube, while lung and trachea progenitors are located on the ventral side^93–96^. Given this, we hypothesized that the dorsal–ventral segregated lung 1 and lung 2 populations observed in our data at the 8–12 ss correspond to lung/trachea and esophagus progenitors, respectively.

To investigate whether this was the case, we explored the set of genes that were differentially expressed between the lung 1 and lung 2 populations. As expected, we noted differences in genes associated with dorsal–ventral patterning (Fig. 4e and Supplementary Table 9), including differential expression of *Chrd*, a known dorsal–ventral regulator^97^, and *Osr1*, which is necessary for lung specification and whose loss results in notably fewer respiratory progenitors at E9.5 and reduced lung size^98^ (Fig. 4f). Additionally, the T-box gene *Tbx1*, which is known to be expressed in the embryonic mesoderm and later in the pharyngeal region and otic vesicle^99^, was more strongly expressed on the dorsal side of the gut tube^99,100^. It has been demonstrated that mutants that show esophageal atresia/trachea–esophageal atresia display abnormal expression of *Tbx1* (ref. ^101^) and *Tbx2* (ref. ^100^). To independently validate these asymmetric dorsal–ventral expression patterns, we used whole-mount hybridization chain reaction (HCR) combined with three-dimensional (3D) imaging to study the coexpression of *Tbx1* (dorsal) and *Shh* (ventral) as well as *Smoc2* (dorsal) and *Tbx3* (ventral) (Fig. 4g–j and Extended Data Fig. 10). This confirmed the observations from our seqFISH data, with clear dorsal–ventral localization of these genes observed in the foregut region of the gut tube corresponding to the lung 1 and lung 2 populations.

Taken together, the spatially resolved expression pattern of genes involved in esophagus, lung and trachea development and the anatomical position of the lung 1 and lung 2 populations indeed indicate that the dorsal lung 2 population corresponds to esophageal progenitors, while the ventral lung 1 population represents lung and trachea progenitors. Although little is known about the transcriptional identity of the early dorsal and ventral endodermal population that ultimately gives rise to the trachea and esophagus, Kuwahara et al. recently used scRNA-seq at E10.5 and E11.5 to better define the transcriptional identity of the developing esophagus, trachea and lung^93^. Several of the identified markers already show dorsal–ventral asymmetries in our data, including the lung and trachea markers *Isl1, Isx2* and *Isx3* and the esophagus marker *Sox2* (refs. ^93,94,102^). More broadly, previous studies have shown that the commitment of progenitor cells to either the lung/trachea or the esophagus is coordinated by the interplay of several transcription factors and signaling pathways that also regulate the dorsal–ventral specification of the gut tube^93^. Specifically, it was shown in E9.5 embryos that the expression of *Wnt2*/*Wnt2b*, *Bmp4* and *Nkx2-1* is enriched in the ventral foregut and respiratory mesenchyme, while the BMP signaling inhibitor genes *Nog* and *Sox2* are enriched in the dorsal foregut^3,95,103–105^. Consistent with this, we observe strong expression of *Wnt2*/*Wnt2b* and *Bmp4* in the splanchnic mesoderm surrounding the ventral lung 1 population, indicating an early role of WNT and BMP signaling in lung and trachea instruction (Extended Data Fig. 9). Taken together our data suggest that cells committed to the lung and trachea (lung 1) or the esophagus (lung 2) are physically separated at the 8–12 ss, approximately 12–24 h earlier than previously reported.

## Discussion

We have combined cutting-edge experimental approaches with advanced computational analyses to generate a comprehensive map of how gene expression varies in space across sagittal sections of an entire mouse embryo at the 8–12 ss of development. Previous studies using scRNA-seq have computationally reconstructed developmental trajectories based on gene expression, but, in the absence of cell-specific spatial information, it has been impossible to define how cell states are correlated with the position of cells within the embryo or to understand how the local signaling environment to which they are exposed might impact their molecular signature and their ultimate fate. Conversely, although pioneering studies have mapped the expression of individual developmental genes at single-cell resolution, the ability to stitch together multiple independent in situ maps into a complete, single-cell resolution map has not been possible due to inevitable fine-scale variations in local cellular organization between embryos.

By combining our high-resolution seqFISH map with scRNA-seq, we have delineated the precise location of distinct cell types within a single reference scaffold. To illustrate the potential of this resource, we have shown how it can provide insight into the formation of the MHB and, in particular, the etiology of cell types along the nascent gut tube. In the latter case, we have added an additional axis of resolution to previous studies by uncovering dorsal–ventral patterning associated with the commitment of cells toward either the esophagus or the lung and trachea. To enable this analysis, we developed computational tools for probe design and for integrating and imputing data, and we developed strategies for downstream analysis, including modeling spatial heterogeneity and performing virtual dissections. This provides a robust experimental and computational framework for future studies both in the mouse and in other biological systems.

In the future, generation of comprehensive cell resolution spatial maps at additional stages of mouse development will allow for spatiotemporal analysis and provide insight into the complex processes associated with cell fate specification during gastrulation and organogenesis. Three-dimensional whole-mount maps would further resolve the processes associated with embryo patterning, in particular processes that are associated with the left–right axis. Moreover, the recent development of image-based cell lineage-tracing methods, such as Zombie^106^ or intMEMOIR^107^, allow a cell’s lineage to be recorded while preserving spatial information. These methods are compatible with seqFISH and therefore afford the possibility to record spatial gene expression profiles and cell history from the same cell in intact tissue. Combining these lineage-tracing methods with spatial transcriptomics will improve our ability to decipher the mechanisms underpinning cell fate choice and tissue patterning.

## Methods

### Library design

We selected genes whose expression patterns discriminated cells from different labeled cell types described in the scRNA-seq data of Pijuan-Sala et al.^6^. To do this, we used the scran function findMarkers^108^, with the option ‘pval.type = ’any’‘, testing against an absolute fold change of 0.5. This was performed separately at each developmental stage of the Gastrulation atlas (E6.5–E8.5, in 0.25-d steps), and only cell types with more than 10 cells at any given stage were included in the stage analysis. Genes were excluded if the upper quartile of the normalized count across cells in any individual cell type was greater than 20. This was performed to prevent the inclusion of highly expressed genes that may compromise imaging. The ‘top’ five genes per cell type were saved from each stage, and the union of these genes was taken across stages. Top genes were defined by the findMarkers ‘Top’ column, which identifies a minimal number of genes required to separate any cell type from any other. The gene panel was evaluated on a per gene basis to exclude any genes that were too short or repetitive to produce reliable FISH probes. Additionally, for each cell type, the panel of genes was manually curated to ensure that the total normalized RNA count across cells for each cell type was less than 300 (Extended Data Fig. 1). For each cell, we calculated the estimated number of detectable transcripts by exponentiating the size factor-standardized log counts for each cell and gene in the Gastrulation atlas dataset. This implicitly assumes a similar sensitivity/detection rate of transcripts between scRNA-seq and seqFISH technology. Based on guidance from previous seqFISH studies, we considered a total of 200 detected transcripts as an ideal maximum for any given cell to avoid the risk of optical crowding. Finally, after determining a suitable set of cell-type marker genes, we manually added genes of interest (especially transcription factors) to the panel and iteratively performed the ‘fluorescent load’ testing and gene removal as described in the previous two sentences. In total, we selected 387 genes, of which 351 genes were detected using seqFISH and 36 were detected using non-barcoded sequential smFISH imaging.

### Primary probe design

Gene-specific primary probes were designed for the selected 351 seqFISH and 36 smFISH genes, as previously introduced by Eng et al.^109^ (Supplementary Table 1). To design 30-nucleotide primary probe sequences for the 351 selected seqFISH and 36 smFISH genes, we extracted 30-nucleotide sequences of each of the selected genes using the coding region of each gene. The mask genome and annotation from the University of Santa Cruz (UCSC) were used to look up the gene sequences. All probe sequences were selected to have a GC content in the range from 45 to 65% and to not have five or more consecutive bases. Genes with more than 48 primary probes were used as a secondary filter to remove off targets. Any gene that did not achieve a minimum of 28 probes for seqFISH and 17 probes for smFISH was dropped. To validate the specificity of the generated primary probes and to minimize off targets, we performed a BLAST search against the mouse transcriptome, and all BLAST hits other than the target gene with a 15-nucleotide match were considered off targets. To avoid off-target hits between the primary probes, a second round of optimization was performed. We constructed a local BLAST database from the primary probe sequences, and probes that were predicted to hit more than seven times by all of the combined primary probes in the BLAST database were iteratively dropped from the probe set until no more than seven off-target hits existed for each primary probe sequence.

### Readout probe design

Readout probes of 15-nucleotide length were designed as previously introduced by Shah et al.^26^. In brief, the probe sequences were randomly generated with combinations of A, T, G or C nucleotides, with a GC content in the range of 40–60%. To validate the specificity of the generated readout sequences, we performed a BLAST search against the mouse transcriptome. To minimize cross-hybridization of the readout probes, all probes with 10 contiguously matching sequences between the readout probes were removed. The reverse complements of these readout probe sequences were included in the primary probe, as described below (Supplementary Table 1).

### Primary probe library construction

The primary probe library, consisting of 15,989 probes for 387 genes (17–48 per gene/average of 41.32 per gene), was ordered as an oligoarray pool from Twist Bioscience. Each probe for barcoded mRNA seqFISH was assembled out of 30-nucleotide mRNA complementary sequence for in situ hybridization, four 15-nucleotide gene-specific readout sequences separated by a 2-nucleotide spacer and two flanking primer sequences to allow for PCR amplification of the probe library (primary barcoded mRNA seqFISH probes, 5′-(primer 1)-(readout 1)-(readout 2)-(probe)-(readout 3)-(readout 4)-(primer 2)-3′). Each of the probes for non-barcoded sequential smFISH were assembled in the same way, with the exception that the sequence for the four readout sequences was the same for all four readout sequences (primary non-barcoded sequential smFISH probes, 5′-(primer 1)-(readout 1)-(readout 1)-(probe)-(readout 1)-(readout 1)-(primer 2)-3′). We used validated primer and 84 readout sequences previously used in seqFISH+^25^. Next, the probe library was amplified as previously described^24,25,109–111^. In brief, limited-cycle PCR was used to generate in vitro transcription template using primer 1 and primer 2. Next, the PCR product was purified using a QIAquick PCR Purification kit (Qiagen, 28104) following the manufacturer’s instructions. Subsequently, the purified PCR product was used for in vitro transcription (NEB, E2040S) followed by reverse transcription (Thermo Fisher, EP7051) with the forward primer containing a uracil nucleotide^112^. Next, the forward primer sequence was removed by cleaving off the uracil nucleotide. The probes were subjected to a 1:30 dilution of uracil-specific excision reagent enzyme (NEB, N5505S) for about 24 h at 37 °C. The single-stranded DNA was alkaline hydrolyzed with 1 M NaOH at 65 °C for 15 min, followed by neutralization with 1 M acetic acid to remove the remaining RNA templates. Next, the probe library was purified by ethanol precipitation to remove residual nucleotides and by phenol–chloroform extraction to remove the proteins. Finally, the amplified primary probe library was dried by speedvac and resuspended at a concentration of 40 nM per probe in primary probe hybridization buffer composed of 40% formamide (Sigma, F9027), 2× SSC and 10% (wt/vol) dextran sulfate (Sigma, D8906). The probes were stored at −20 °C.

### Readout probe synthesis

Fifteen-nucleotide readout probes were ordered from Integrated DNA Technologies (IDT), conjugated to Alexa Fluor 488, Cy3B and Alexa Fluor 647 as indicated in Supplementary Tables 2 and 3. All readout probes were stored at −20 °C.

### Encoding strategy

In this experiment we used a 12-pseudocolor encoding scheme, as described previously^26,109^. In brief, 12 pseudocolors were equally separated across three fluorescent channels (Alexa Fluor 488, Cy3B and Alexa Fluor 647). The 12-pseudocolor imaging was repeated four times, resulting in 12^4^ (20,736) unique barcodes. Additionally, an extra round of pseudocolor imaging was performed to obtain error-correctable barcodes, as previously introduced^24^. In this experiment, 351 genes were encoded across all channels (Supplementary Table 2).

### Coverslip functionalization

Coverslips were functionalized as previously described^25^. In brief, coverslips (Thermo Scientific, 3421) were washed in nuclease-free water, followed by an immersion in 100% ethanol (Koptec). Subsequently, coverslips were air dried and cleaned using a plasma cleaner on the high setting (PDC-001, Harrick Plasma) for 5 min. Then, the coverslips were immersed in 1% bind-silane solution (GE, 17-1330-13) made in pH 3.5 10% (vol/vol) acidic ethanol solution for 1 h at room temperature. Next, coverslips were rinsed three times in 100% ethanol and heat dried in an oven at >90 °C for 30 min. Then, the coverslips were treated with 100 µg ml^–1^ of poly-d-lysine (Sigma, P6407) in water for a minimum of 1 h at room temperature. Afterwards, coverslips were washed three times in nuclease-free water and air dried. Functionalized coverslips can be stored for up to 1 week at 4 °C.

### Mice

Experiments, with the exception of the HCR experiment (see below), were performed in accordance with EU guidelines for the care and use of laboratory animals, and under the authority of appropriate UK governmental legislation. Eight- to 12-week-old WT C57BL/6J mice (Charles Rivers) were used, with the exception of the HCR experiment. For the HCR experiment, WT CD-1 mice (Charles Rivers) were used. Natural mating was set up between males and 4- to 6-week-old virgin females, with 12:00 of the day of vaginal plug considered to be E0.5. Mice were maintained in accordance with guidelines from Memorial Sloan Kettering Cancer Center (MSKCC) Institutional Animal Care and Use Committee (IACUC) under protocol number 03-12-017 (principal investigator A.-K.H.). All mice used in this project were housed under a 12-h light/12-h dark cycle, with constant access to food and water. No sex selection of the used embryos was performed.

### Tissue preparation

Embryos were dissected from the uteri, washed in M2 medium (Sigma Aldrich, 7167) and immediately placed in 4% paraformaldehyde (PFA) (Thermo Scientific, 28908) in 1× PBS (Invitrogen, AM9624) for 30 min at room temperature. The embryos were then washed and immersed in 30% RNase-free sucrose (Sigma Aldrich, 84097) in 1× PBS at 4 °C until the embryo sank to the bottom of the tube. Afterwards, each embryo was positioned in a sagittal orientation in a tissue base mold (Sakura, 4162) in optimal cutting temperature (OCT) compound solution (Sakura, 4583), frozen in dry ice isopropanol (VWR, 20842) and stored at −80 °C. Tissue sections (20 µm) were cut using a cryotome, collected on the functionalized coverslips and stored at −80 °C.

### seqFISH using tissue sections

Tissue sections were postfixed with 4% PFA in 1× PBS for 15 min at room temperature to stabilize the DNA, RNA and overall sample structure. The fixed samples were permeabilized with 70% ethanol for 1 h at room temperature. Then, the tissue slices were cleared with 8% SDS in 1× PBS for 20 min at room temperature. The cleared sample was washed with 70% ethanol and then air dried. Samples were blocked for a minimum of 2 h in blocking solution (1× PBS supplemented with 0.25% Triton X-100, 10 mg ml^–1^ bovine serum albumin (BSA; Thermo Fisher, AM2616) and 0.5 mg ml^–1^ salmon sperm DNA (Thermo Fisher, AM9680)) at room temperature in a humidified chamber. Anti-pan-cadherin (Abcam, ab22744), anti-N-cadherin (13A9; Cell Signaling Technology, 14215), anti-β-catenin (15B8; Abcam, ab6301) and anti-E-cadherin (clone 36; BD Biosciences, 610181) were diluted 1:200 in blocking solution and incubated for 2 h at room temperature. Samples were washed three times in 1× PBS supplemented with 0.1% Triton X-100 (PBS-T) before incubating with anti-mouse IgG secondary antibody conjugated to CCTTACACCAACCCT oligo diluted 1:500 in blocking solution for at least 2 h at room temperature. Next, the samples were washed three times in 1× PBS-T. The samples were postfixed with 4% PFA in 1× PBS for 15 min followed by three 10-min washes in 2× SSC (Thermo Fisher, 15557036). The samples were dried and hybridized for 24–36 h with the probe library (~2.5 nM per probe), 1 nM *Eef2* probe set A and B (Supplementary Table 1) and 1 µM locked nucleic acid (LNA) oligo-d(T)30 (Qiagen) in primary probe hybridization buffer composed of 40% formamide (Sigma, F9027), 2× SSC and 10% (wt/vol) dextran sulfate (Sigma, D8906) in a humid chamber at 37 °C. The hybridization samples were washed with 40% formamide wash buffer (40% formamide, 0.1% Triton X-100 in 2× SSC) for 30 min at 37 °C, followed by three rinses with 2× SSC. Then, the samples were hybridized for at least 2 h with 200 nM tertiary probe (/5Acryd/AG GGT TGG TGT AAG GTT TAC CTG GCG TTG CGA CGA CTA A) in EC buffer made of 10% ethylene carbonate (Sigma, E26258), 10% dextran sulfate (Sigma, D4911) and 4× SSC. The samples were washed for 5 min in a 10% formamide washing buffer (10% formamide, 0.1% Triton X-100 in 2× SSC), followed by two 5-min washes in 2× SSC. The samples were treated with 0.1 mg ml^–1^ Acryoloyl-X succinimidyl ester (Thermo Fisher, A20770) in 1× PBS for 30 min at room temperature. Then, the samples were rinsed three times with 2× SSC and postfixed with 4% PFA in 1× PBS for 15 min, followed by three washes in 2× SSC. Next, the samples were incubated with 4% acrylamide/bis (1:19 crosslinking) hydrogel solution in 2× SSC for 30 min. The hydrogel solution was aspirated, and the sample was covered with 20 µl of degassed 4% hydrogel solution containing 0.05% ammonium persulfate (APS) (Sigma, A3078) and 0.05% *N*,*N*,*N*′,*N*′-tetramethylenediamine (TEMED) (Sigma, T7024) in 2× SSC. The sample was sandwiched by a GelSlick functionalized slide (Lonza, 50640). The samples were transferred to a home-made nitrogen gas chamber and incubated for 30 min at room temperature before transferring to 37 °C for at least 3 h. After polymerization, the slides were gently separated from the coverslip, and the hydrogel-embedded tissue was rinsed with 2× SSC three times. Then, the samples were cleared for 3 h at 37 °C using digestion buffer, as previously described^33^. The digestion buffer consisted of 1:100 proteinase K (NEB, P8107S), 50 mM pH 8 Tris-HCl (Invitrogen, AM9856), 1 mM EDTA (Invitrogen, 15575020), 0.5% Triton X-100, 1% SDS and 500 mM NaCl (Sigma, S5150). After digestion, the tissue slices were rinsed with 2× SSC multiple times and then subjected to 0.1 mg ml^–1^ label-X modification for 45 min at 37 °C (ref. ^33^). For further stabilization, the sample was re-embedded in a 4% hydrogel solution, as described above, with a shortened gelation time of 2.5 h. Excess gel was removed with a razor, and the sample was covered with an in-house-made flow cell. The sample was immediately imaged.

### seqFISH imaging

Two tissue sections from two experimental blocks, containing three embryos, were imaged as previously described^25,26^. In brief, the flow cell was connected to an automated fluidics system. First, the sample was stained with 10 µg ml^–1^ DAPI (Sigma, D8417) in 4× SSC, and the FOVs were selected. All rounds of imaging were performed in antibleaching buffer made of 50 mM Tris-HCl pH 8.0 (Thermo Fisher, 15568025), 300 mM NaCl (Sigma, S5150), 2× SSC (Thermo Fisher, 15557036), 3 mM Trolox (Sigma, 238813), 0.8% d-glucose (Sigma, G7528), 1:100 diluted catalase (Sigma, C3155) and 0.5 mg ml^–1^ glucose oxidase (Sigma, G2133). The RNA integrity of the sample was validated by colocalization of the dots of two interspersed *Eef2* probes, each read out by secondary readout probes with distinct fluorophores (Extended Data Fig. 2 and Supplementary Table 3). Sixteen hybridization rounds were imaged for the decoding of the barcoded mRNA seqFISH probes followed by a repeat of the first hybridization. Then, 12 serial hybridization rounds were imaged for 36 non-barcoded sequential smFISH probes, followed by 1 hybridization to visualize the cell segmentation staining using Cy3B conjugated to /5AmMC6/TTAGTCGTCGCAACG. The hybridization buffer for each of the hybridization rounds, excluding the last, contained three unique readout probes, each consisting of a unique 15-nucleotide probe sequence conjugated to either Alexa Fluor 647 (50 nM), Cy3B (50 nM) or Alexa Fluor 488 (50 nM) in EC buffer, as described above (Supplementary Tables 2 and 3). The hybridization buffer for the cell segmentation staining contained one unique 15-nucleotide probe sequence conjugated to Alexa Fluor 647. The hybridization buffer mixes for the 30 rounds of hybridization were stored in a deep-bottom 96-well plate and were added to the hybridization chamber by an automated sampler system, as described previously^25^. The tissue section was incubated in the hybridization solution for 25 min at room temperature in the dark. Next, the sample was washed with 300 µl of 10% formamide wash buffer to remove excess and non-specific readout probes. The sample was rinsed with 4× SSC and subsequently stained with 10 µg ml^–1^ DAPI in 4× SSC for 1.5 min. Then, the flow chamber was filled with antibleaching buffer, and all selected FOVs of the sample were imaged. The microscope used was a Leica DMi8 stand equipped with a Yokogawa CSU-W1 spinning disk confocal scanner, an Andor Zyla 4.2 Plus sCMOS camera, a Leica ×63, 1.40-NA oil objective, a motorized stage (ASI MS2000), lasers from CNI and filter sets from Semrock. For each FOV, snapshots were acquired with 4-µm *z* steps for six *z* slices. After imaging, the readout probes were stripped off using 55% wash buffer (55% formamide, 0.1% Triton X-100 in 2× SSC) by incubating the sample for 4 min, followed by a 4× SSC rinse. Serial hybridization and imaging were repeated for 29 rounds. Integration of the automated fluidics delivery system and imaging was controlled by a custom script written in µManager^113^.

### Image processing

To remove the effects of chromatic aberration, 0.1-mm TetraSpeck bead (Thermo Scientific, T7279) images were first used to create geometric transforms to align all fluorescence channels. Tissue background and autofluorescence were then removed by dividing the initial background with the fluorescence images. To correct for the non-uniform background, a flat field correction was applied by dividing the normalized background illumination with each of the fluorescence images while preserving the intensity profile of the fluorescent points. The background signal was then subtracted using the ImageJ rolling ball background subtraction algorithm with a radius of 3 pixels and filtered with a despeckle algorithm to remove any hot pixels.

### Image registration

Each round of imaging contained the 405 channel, which included the DAPI stain of the cell. For each FOV (for example tile), all of the DAPI images from every round of hybridization were aligned to the first image using a two-dimensional (2D) phase correlation algorithm.

### Cell segmentation

For semiautomatic cell segmentation, the membrane stains β-catenin, E-cadherin, N-cadherin and pan-cadherin were aligned to the first hybridization round using DAPI and subsequently trained with Ilastik^35^, an interactive supervised machine learning toolkit, to output probability maps, which were used in the Multicut^114^ tool to produce 2D-labeled cells for each *z* slice. For image analysis, potential mRNA transcript signals were located by finding the local maxima in the processed image above a predetermined pixel threshold, manually calculated for one FOV and adjusted for the remainder according to the number of expected potential spots per cell. The transcript spots were assigned to the corresponding labeled cells according to location, thereby generating a gene–cell count table.

### Barcode calling

Once all potential points in all channels of all hybridizations were obtained, dots were matched to potential barcode partners in all other channels of all other hybridizations using a 2.45-pixel search radius to find symmetric nearest neighbors. Point combinations that yielded only a single barcode were immediately matched to the on-target barcode set. For points that matched to multiple barcodes, first the point sets were filtered by calculating the residual spatial distance of each potential barcode point set, and only the point sets giving the minimum residuals were used to match to a barcode. If multiple barcodes were still possible, the point was matched to its closest on-target barcode with a hamming distance of 1. If multiple on-target barcodes were still possible, then the point was dropped from the analysis as an ambiguous barcode. This procedure was repeated using each hybridization as a seed for barcode finding, and only barcodes that were called similarly in at least three of four rounds were validated as genes. For more details regarding the seqFISH method, please refer to Shah et al.^24^.

### smFISH processing

For the 36 genes that were probed using smFISH, 12 sequential rounds of imaging across three fluorescent channels (corresponding to Alexa Fluor 647, Cy3B and Alexa Fluor 488, respectively) were used (Supplementary Table 3). Assignment of an optimal light intensity threshold to separate background noise from hybridized mRNA molecules poses an additional challenge for these data because, unlike the seqFISH probed transcripts, each gene’s expression is measured only over a single round of hybridization.

To address this problem, we manually assigned a threshold for three randomly selected FOVs in the first experimental block (corresponding to embryos 1 and 2) and three FOVs in the second experimental block (embryo 3) for all fluorescent channels and all hybridization rounds. The choice of threshold was motivated by considering the minimum value at which we acquire nearly complete loss of dots in cell-free areas, which we expect should only contain background signal. We then assessed the relationship between the channel and hybridization round and the manually selected thresholds, observing that intensity thresholds are highly channel specific but do not vary as a function of hybridization round (Supplementary Fig. 15). Accordingly, for each channel, hybridization round and experimental block, we assigned the intensity threshold as the average across all manually selected thresholds.

We then visually assessed the spatial distribution of selected spots for each gene, embryo and *z* slice. While most of the estimated intensity thresholds resulted in spatially coherent expression patterns across all embryos, we noticed a strong channel, FOV-specific effect for some genes. Specifically, in the first experimental block, genes probed with Alexa Fluor 647 exhibited substantial background signal in FOVs 39, 40 and 44. Given that the effect is highly specific to this channel, it is likely an artifact of the imaging experiment. For these genes and FOVs, manual examination of a wide range of appropriate intensity thresholds failed to identify a threshold at which the background noise was eliminated (Supplementary Fig. 15). Consequently, we discarded these fields when evaluating the performance of our imputation strategy (see below).

### Whole-mount HCR on E8.75 mouse embryos

HCR fluorescent in situs where carried out as described in^115,116^, with the modification of using 60 pmol of each hairpin per 0.5 ml of amplification buffer. Hairpins were left for 12–14 h at room temperature for saturation of amplification to achieve the highest levels of signal to noise^117^. Split initiator probes (V3.0) were designed by Molecular Instruments.

### HCR imaging

All images were obtained on a Zeiss 880 laser-scanning confocal microscope with a ×10 objective and 6.74-µm *z*-step sizes. Tile-scanned *z* stacks were stitched in Zen software and rendered in 3D in Imaris (v9.6, Bitplane).

### Downstream computational analysis

#### Quality control and filtering

To lower the chance of counting cells multiple times in contiguous *z* slices, we selected two *z* slices (denoted 1 and 2 hereafter) for further analysis corresponding to two parallel tissue layers 12 µm apart. We then removed segmented regions most likely to correspond to empty space rather than cell-containing regions by testing whether a putative cell’s square root-transformed segmented area was larger than expected (*Z* test; FDR threshold of 0.01). Of the remaining segmented regions, we considered segments containing at least 10 detected mRNA molecules corresponding to at least five unique genes as true cells.

#### Cell neighborhood network construction

To construct a cell neighborhood network, for each cell within a given embryo and *z* slice, we extracted the polygon representation of the cell’s segmentation corresponding to a set of vertex coordinates. We then calculated an expanded segmentation by constructing a new polygon where each expanded vertex was lengthened along the line containing the original vertex and the center of the polygon. We performed a multiplicative expansion of 1.3 for each vertex. To construct the cell neighborhood network, we then identified the other cells in which segmentation vertices were found to be within the expanded polygon. Cell neighborhood networks were considered separately for each embryo and *z* slice combination.

#### Gene expression quantification per cell

We calculated normalized expression log counts for each cell using scran’s logNormCounts function^108^, with size factors corresponding to the total number of mRNAs (excluding the sex-specific gene *Xist*) identified for each cell. Size factors were scaled to unity, and a pseudocount of 1 was added before the log counts were extracted. For the majority of downstream analyses, such as differential gene expression, we specifically included biological and technical variables (that is, *z* slice and FOV) as covariates. However, for the task of harmoniously visualizing gene expression in spatial coordinates, we extracted ‘batch-corrected expression’ values for each gene. This was done by first performing batch correction using the MNN method, implemented with fastMNN in the scran package^108^, with batch variables corresponding to *z* slice and FOV. For interpretable visualization, for each gene, we extracted the reconstructed expression values following batch correction and rescaled these to correspond to the distribution of expression values before batch correction.

#### Clustering gene expression

To identify unsupervised clusters, we first performed multibatch-aware principal component analysis (PCA) on the normalized log counts using the multiBatchPCA function in scran^108^, with *z* slice and FOV as batch variables using all genes except *Xist* as input to extract 50 PCs. We then performed batch correction using the MNN approach, resulting in a corrected reduced dimension embedding of cells. To identify clusters, we estimated a shared nearest neighbor network, followed by Louvain network clustering. To further extract unsupervised subclusters, for each set of cells belonging to a given cluster, we performed highly variable gene selection to select genes with a non-zero estimated biological variance, excluding the sex-specific gene *Xist*. Using these selected genes, we performed batch-aware PCA to extract 50 PCs, followed by batch correction, shared nearest neighbor network construction and Louvain clustering similar to what was performed for all cells.

#### Joint analysis with Gastrulation atlas

We downloaded the E8.5 Pijuan-Sala et al.^6^ 10x Genomics scRNA-seq dataset from the Bioconductor package MouseGastrulationData and performed batch-aware normalization using the multiBatchNorm function in the scran package^108^ before extracting cells that correspond to a known cell type with at least 25 cells. Cell types associated with the somitic and paraxial mesoderm were further refined using labels assigned by Guibentif et al.^118^ (personal communication); blood subtypes (erythroid 1, erythroid 2 and erythroid 3 and blood progenitors 1 and 2) were collapsed to the two major groups, ExE mesoderm was renamed to lateral plate mesoderm and pharyngeal mesoderm was renamed to splanchnic mesoderm. Subsequently, only genes probed by both the scRNA-seq and seqFISH assays were kept for this analysis. We then jointly embedded the normalized log counts of each of the two datasets by performing batch-aware PCA with 50 components (excluding the sex-specific gene *Xist*) via the multiBatchPCA function in scran, with batch variables corresponding to sequencing runs in the Gastrulation atlas and FOV and *z* slice for the seqFISH data. We corrected for platform- and batch-specific effects using the MNN method (fastMNN^55^), ensuring that merge ordering is such that Gastrulation atlas batches are merged first (ordered by decreasing number of cells). This joint embedding of the Gastrulation atlas and seqFISH dataset was further reduced in dimension using UMAP, implemented by calculate UMAP in scran^108^, to allow the data to be visualized in two dimensions.

#### Cell type identification

To assign a cell-type label to each seqFISH cell, we considered the Gastrulation atlas cells that it was closest to in the batch-corrected space. We considered the *k*-nearest cells, with the distance from the seqFISH cell to its Gastrulation atlas neighbors being computed as the Euclidean distance among all of the batch-corrected PC coordinates. We set the number of nearest neighbors, *k*, to 25. Ties were broken by favoring the cell type of those closest in distance to the query cell. We calculated a ‘mapping score’ for each query cell as the proportion of the majority cell type present among the 25 closest cells.

To further refine the predicted cell types, we performed joint clustering of the Gastrulation atlas and seqFISH cells by building a shared nearest neighbor network on the joint PCs followed by Louvain network clustering. Additionally, we further subclustered the output by building a shared nearest neighbor network on the cells corresponding to each cluster followed by Louvain network clustering. We then inspected the relative contribution of cells to each subcluster as well as the expression of marker genes to identify subclusters that potentially required manual reannotation, either due to small differences in composition in the reference atlas or in the gene expression profile (Extended Data Fig. 3). We also identified a set of subclusters that were likely associated with low-quality cells, defined by lower total mRNA counts. Furthermore, we performed virtual dissection on regions corresponding anatomically to the developing gut tube and for these cells reclassified those that were ‘Surface ectoderm’ as ‘Gut tube’.

#### Simulation selecting fewer genes for data integration

For the specific task of recovering cell-type identity, we investigated whether fewer genes would be sufficient. To do this, we randomly selected subsets of genes from the 351 gene set, corresponding to approximately 10, 20, 30, …, 90% of the genes, repeated five times for each subset. Because there is a lack of ground truth of the cell-type labels for the seqFISH data, we assessed the cell-type classification accuracy relative to the full probe set, that is, we made the assumption that the classified cell type using the 351 genes is the true label, thus measuring the degree of loss of accuracy from this labeling. While ground truth labels are available for the Gastrulation atlas dataset, for consistency we calculated the relative accuracy following resubstitution classification for these cells by also treating the classified cell type using the 351 genes as the true label.

Any difference in cell-type recovery accuracy between the seqFISH and Gastrulation atlas data could be attributed to the experimental strategy (scRNA-seq versus seqFISH) or to the fact that the Gastrulation atlas data was initially mined for these informative genes, and, as a result, the resubstitution classification accuracy may be inflated for these cells. Thus, we extracted the host WT cells of the E8.5 WT/WT control chimera from Pijuan-Sala et al.^6^, which served as an independent validation set, representing a dataset that was not mined for informative genes but also corresponds to the same experimental strategy as the Gastrulation atlas (scRNA-seq).

We performed joint integration of these three datasets using the randomly selected gene subsets and calculated the relative cell-type classification accuracy compared to the full gene set for each dataset.

#### Subclustering of mixed mesenchymal mesoderm cells

To analyze the mixed mesenchymal mesoderm population, we performed highly variable gene selection for these cells only using the ‘modelGeneVar’ function in scran^108^ and performed PCA (excluding the sex-specific gene *Xist*) on the normalized log counts followed by batch correction using MNN, with embryo and *z* slice as batch variables. We then further reduced these corrected PCs into two dimensions using UMAP for visualization purposes. To identify mixed mesenchymal mesoderm subclusters, we estimated a shared nearest neighbor network, followed by Louvain network clustering. We then performed differential expression analysis on the seqFISH genes and on the imputed gene expression values (described further below) using the ‘findMarkers’ function in scran^108^ and Gene Ontology enrichment analysis as described below. To further identify the spatial context for the mixed mesenchymal mesoderm, for each cluster, we extracted the cells that appear as direct contact neighbors with any cell belonging to the cluster and recorded their corresponding cell type. To assess the relative association of each mixed mesenchymal mesoderm subcluster to the Gastrulation atlas^6^, we calculated a weighted score per Gastrulation atlas cell and mixed mesenchymal mesoderm subcluster, corresponding to the average ranking of the Gastrulation atlas cell among the top 25 nearest neighbors for each mixed mesenchymal mesoderm subcluster cell.

#### Spatial heterogeneity testing per cell type

We identified genes with a spatially heterogeneous pattern of expression using a linear model with observations corresponding to each cell for a given cell type and with outcome corresponding to the gene of interest’s expression value. For each gene, we fit a linear model including the embryo and *z* slice information as covariates as well as an additional covariate corresponding to the weighted mean of all other cells’ gene expression values. The weight was computed as the inverse of the cell–cell distance in the cell–cell neighborhood network.

More formally, let *x*_*ij*_ be the expression of gene *i* for cell *j*. Calculate $x_{ij}^ \ast$ as the weighted average of other *K* cells’ expression weighted by distance in the neighborhood network

$$x_{ij}^ \ast = \mathop {\sum }\limits_{k \in K} \frac{{x_{ik}}}{{D_{jk}}}$$

where

$$D_{jk} = d\left( {v_j,v_k} \right)$$

is the path length in the neighborhood network between vertices corresponding to cells *j* and *k*. We then fit the linear model for each gene

$$x_i = \beta _0 + \beta _1x_i^ \ast + \beta _2e + \beta _3z + \beta _4e \times z + \epsilon.$$

Here, *e* and *z* correspond to the embryo and *z* slice identity of the cells, respectively, and *ε* represents random normally distributed noise. The *t*-statistic corresponding to *β*_1_ is reported here as a measure of spatial heterogeneity for the given gene, a large value corresponding to a strong spatial expression pattern of the gene in space, especially among its neighbors.

#### Subclustering of developing brain cells

To further subcluster the developing brain cells, we extracted the Gastrulation atlas cells corresponding to E8.5 that were classified as forebrain/midbrain/hindbrain. For these cells, we identified genes to further cluster by using the scran function modelGeneVar^108^ to identify highly variable genes with non-zero biological variability, excluding the sex-specific gene *Xist*. For these genes, we extracted the cosine-standardized log counts, which were standardized against the entire transcriptome. We then performed batch correction using the MNN method on batch-aware PC coordinates, where batches corresponded to the sequencing samples. Using this batch-corrected embedding, we estimated a shared nearest neighborhood network and performed Louvain network clustering. To relate these brain subcluster labels to the seqFISH data, we extracted the nearest neighbor information (as described in Cell type identification) for seqFISH cells corresponding to forebrain/midbrain/hindbrain and classified their brain subcluster label using *k*-nearest neighbors with *k* = 25 and closest cells breaking ties. We then named these subclusters based on marker gene expression, including a class that may be technically driven (NA class).

#### Cell–cell contact map inference

We constructed cell–cell contact maps for multiple cell annotation labelings, including mapped cell types, subclusters within each cell type and mapped gut tube subtypes. To do this, for each embryo and *z* slice combination, we extracted the cell neighborhood network and cell-level annotation. We then generated cell–cell contact maps by first calculating the number of edges for which a particular pair of annotated groups was observed. We then randomly reassigned (500 times) the annotation by sampling without replacement and calculated the number of edges for all pairs of annotated groups. To construct the cell–cell contact map, we reported the proportion of times the randomly reassigned number of edges was larger than or equal to the observed number of edges. Small values correspond to the pair of annotation groups being more segregated, and large values correspond to them being more integrated in physical space than a random allocation. To combine these cell–cell contact maps for each embryo and *z* slice combination, we further calculated the element-wise mean for each pair of cell labels. We visualized this in a heat map, ordering the annotation groups using hierarchical clustering with Euclidean distance and complete linkage. In the case of the gut tube subtypes, we ordered these classes by the anterior–posterior ordering given by Nowotschin et al.^2^. In the brain subtypes, we ordered these classes by their approximate anatomical location, from the forebrain to the hindbrain region.

#### Gene Ontology enrichment analysis

To functionally annotate sets of gene clusters, we performed gene set enrichment analysis using mouse Gene Ontology terms with between 10 and 500 genes appearing in each dataset and at least 1 gene appearing from the testing scaffold^119^ using Fisher’s exact test to test for overrepresentation of genes and using all scHOT-tested genes as the gene universe. An FDR-adjusted *P* < 0.05 was considered to be statistically significant.

#### Imputation

Below we outline the different elements of our strategy for imputing the spatially resolved expression of genes not profiled using seqFISH.

##### Intermediate mapping

First, for each gene in the seqFISH library (excluding the sex-specific gene *Xist*), we performed an intermediate mapping to align each seqFISH cell with the most similar set of cells in the scRNA-seq dataset. To perform the mapping we excluded the gene of interest and used the remaining 349 genes (351 seqFISH genes – *Xist* – gene of interest), resulting in 350 intermediate mappings overall. The leave-one-gene-out mapping approach was used to assess whether the intermediate mapping strategy outlined below could be used to estimate the expression counts of the omitted gene.

Similar to the integration strategy described earlier for assigning cell-type labels, for each embryo and *z* slice, we concatenated the cosine-normalized seqFISH counts with the cosine-normalized expression values from the Gastrulation atlas scRNA-seq data^6^. We performed dimensionality reduction using ‘multibatchPCA’ (using 50 PCs) and performed batch correction using the ‘reducedMNN’ function implemented in scran^108^. Next, for each cell in the seqFISH dataset that was assigned a cell-type identity in the earlier integration, we used the ‘queryKNN’ function in BiocNeighbors to identify its 25 nearest neighbors in the scRNA-seq data. Finally, for each seqFISH cell, the expression count of the gene of interest is estimated as the average expression of the corresponding gene across the cell’s 25 nearest neighbors.

##### Performance evaluation

For each mapped gene, its performance score is calculated as the Pearson correlation (across cells) between its imputed values and its measured seqFISH expression level. To estimate an upper bound on the performance score (that is, the maximum correlation we might expect to observe), we took advantage of the four independent batches of E8.5 cells that were processed in the scRNA-seq Gastrulation atlas. In particular, we treated one of the four batches as the query set and used the leave-one-out approach described above to impute the expression of genes of interest by mapping cells onto a reference composed of the remaining three batches. Additionally, to mimic the seqFISH imputation, we considered a subset of the Gastrulation atlas data consisting of only those genes that were probed in the seqFISH experiment. Moreover, due to the experimental procedure, some cell types present in the Gastrulation atlas (for example, extraembryonic cell types) were not probed in the seqFISH experiment. Accordingly, we considered only the subset of scRNA-seq profiled cells that were among the nearest neighbors of a seqFISH-mapped cells so that this subset most faithfully resembled the seqFISH data.

Subsequently, for each mapped gene, we computed its prediction score as the weighted Pearson correlation between its imputed expression level and its true expression level. The weights were proportional to the number of times each Gastrulation atlas cell was present among the neighbors of a seqFISH cell across all profiled genes.

Finally, for each gene probed in the seqFISH experiment, we define its normalized imputation performance score as the ratio of its performance score over its prediction score.

##### Final imputation

To perform imputation for all genes, we aggregated across the 350 intermediate mappings generated from each gene probed using seqFISH. Specifically, for each seqFISH cell, we considered the set of all Gastrulation atlas cells that were associated with it in any intermediate mapping. Subsequently, for every cell, we calculated each gene’s imputed expression level as the weighted average of the gene’s expression across the associated set of Gastrulation atlas cells, where weights were proportional to the number of times each Gastrulation atlas cell was present. Thus, the imputed expression profiles for all genes, including those in the overlapping gene set, are on the same scale as the scRNA-seq log count data.

#### MHB detection and virtual dissection

To identify the MHB, we visually assessed the expression of the well-described mesencephalon and prosencephalon marker *Otx2* and the rhombencephalon marker *Gbx2* (Supplementary Fig. 13). We manually selected the physical region where both genes are expressed and defined this as the FOV (black rectangle, Supplementary Fig. 13). Subsequently, within the selected region, we performed a virtual dissection by manually choosing the boundary that best discriminates the expression of *Otx2* and *Gbx2* (Supplementary Fig. 13), and, based on the boundary, we assigned cells either a midbrain or hindbrain identity.

#### Downstream analysis of the MHB region

Differential expression analysis was performed between midbrain- and hindbrain-assigned cells using the scran function ‘findMarkers’ (with an LFC threshold of 0.2 and an FDR-adjusted *P* value threshold of 0.05; Supplementary Table 7).

To perform diffusion analysis of the MHB region, we performed batch correction of the FOVs and *z* slice using the MNN approach, with log counts of all genes excluding the sex-specific gene *Xist* as input. We then used the diffusion pseudotime (DPT) method implemented in the R package destiny^78^ to build a diffusion map with 20 DCs using the cell with maximum value in DC1 as the root cell for DPT estimation. To visualize the DCs in space, we added an estimated vector field to the segmented spatial graphs, with arrow sizes corresponding to the magnitude of change among nearby cells and directions corresponding to the direction with the largest change in the diffusion component. We then identified imputed genes strongly correlated with DPT (absolute Spearman correlation of >0.5) among either midbrain or hindbrain region cells. For smooth expression estimation along the DPT, we split cells into either midbrain or hindbrain regions and extracted fitted values from local regression (loess) for each gene with DPT ranking as the explanatory variable. To further identify genes associated with spatial variation in expression, we performed scHOT^81^ analysis using weighted mean as the underlying higher-order function, with a weighting span of 0.1 on spatial coordinates and using the imputed gene expression values. We then identified the 500 top-ranked significantly spatially variable genes (ensuring also that the FDR-adjusted *P* value was <0.05), clustered their smoothed expression using hierarchical clustering (Supplementary Table 8) and selected the number of clusters using dynamicTreeCut^120^. To visualize spatial expression profiles of clusters, we calculated the mean inferred gene expression value for the genes associated with each cluster.

#### Joint analysis with the Nowotschin et al. dataset

We downloaded the Nowotschin et al. 10x Genomics scRNA-seq counts and associated annotations from the corresponding Shiny web application (https://endoderm-explorer.com/)^2^. We then subset down to E8.75 cells, considering each 10x Genomics sequencing library as a batch variable. We performed highly variable gene (HVG) selection using ‘modelGeneVar’ from the scran package^108^ using the library sample as the blocking variable. We then selected the intersection of these HVGs and the genes appearing in the seqFISH dataset for further analysis. We concatenated the normalized log counts for the Nowotschin et al. and seqFISH datasets and performed dimensionality reduction to 50 PCs using ‘multiBatchNorm’ as implemented in scran^108^. We then performed batch correction using the MNN approach, where the merge order was fixed to first integrate batches from the Nowotschin et al. dataset (ordered by decreasing cell number). We then identified the 10 nearest neighbors of the seqFISH cells to the Nowotschin et al. cells in the corrected reduced dimensional space. Using these nearest neighbors, we classified seqFISH gut tube cells to a cell type defined by Nowotschin et al. A ‘mapping score’ was computed for each cell as the proportion of the nearest neighbors in the Nowotschin et al. data corresponding to the selected class. We performed differential gene expression analysis between the lung 1 and lung 2 groups using ‘findMarkers’ in scran^108^ and also performed differential gene expression analysis between the associated mesodermal cells at most three steps away from the lung 1 or lung 2 cells in the cell–cell neighborhood network.

#### Anterior–posterior axis cell ranking

To calculate the relative position of developing gut tube cells along the anterior–posterior axis, for each embryo, we performed a virtual dissection to visually identify the dorsal and ventral regions of the gut tube. Then, for each embryo and each dorsal or ventral tissue region, we fit a single principal curve model using the R package princurve^121^, with explanatory variables corresponding to the physical coordinates. We then extracted anterior–posterior cell rankings by taking the rank of the fitted arc length from the beginning of the curve, ensuring that the curve always began at the anterior-most position.

#### Joint analysis with Nowotschin et al. and Han et al. datasets

To further understand the relationship between the endodermal and mesodermal layers in the gut tube, we performed a joint analysis between the Nowotschin et al. data (described above) as well as the E8.5 splanchnic mesoderm cells from Han et al.^3^. For the Han et al. data, we performed HVG selection using ‘modelGeneVar’ from the scran package^108^ using the library sample as the blocking variable and then selected the genes that appeared in either the HVG list for Nowotschin et al. or Han et al. and genes that were also present in the seqFISH gene library. We then concatenated the normalized log counts of all three datasets and performed integration (dimensionality reduction, batch correction, further dimensionality reduction for visualization) and cell classification as described above. Thus, for each seqFISH cell, we obtained a classified cell class according to the labels provided by Han et al., including mesodermal subtypes in the splanchnic mesoderm. To further investigate the surrounding mesodermal cells of the gut tube, we used the cell–cell neighborhood network to identify mesodermal cells at most three steps away from a gut tube cell and, for each of these cells, we identified their position as either dorsal or ventral to the gut tube and calculated the mean position along the anterior–posterior axis.

### Reporting Summary

Further information on research design is available in the Nature Research Reporting Summary linked to this article.

## Online content

Any methods, additional references, Nature Research reporting summaries, source data, extended data, supplementary information, acknowledgements, peer review information; details of author contributions and competing interests; and statements of data and code availability are available at 10.1038/s41587-021-01006-2.

## Supplementary information

Supplementary Information Supplementary Figs. 1–15 and legend for Supplementary Tables 1–9.

Reporting Summary

Supplementary Tables Supplementary Tables 1–9.

## Data Availability

The spatial transcriptomic map can be explored interactively at https://marionilab.cruk.cam.ac.uk/SpatialMouseAtlas/, and raw image data are available on request. Processed gene expression data with segmentation information and associated metadata are also available to download and explore online at https://marionilab.cruk.cam.ac.uk/SpatialMouseAtlas/. Processed gene expression data are also available within the R/Bioconductor data package MouseGastrulationData (version 3.13, 10.18129/B9.bioc.MouseGastrulationData).

## Code availability

Scripts for downstream analysis are available at https://github.com/MarioniLab/SpatialMouseAtlas2020.

## References

[1] Argelaguet R (2019). Multi-omics profiling of mouse gastrulation at single-cell resolution. Nature.

[2] Nowotschin S (2019). The emergent landscape of the mouse gut endoderm at single-cell resolution. Nature.

[3] Han L (2020). Single cell transcriptomics identifies a signaling network coordinating endoderm and mesoderm diversification during foregut organogenesis. Nat. Commun..

[4] Arnold SJ, Robertson EJ (2009). Making a commitment: cell lineage allocation and axis patterning in the early mouse embryo. Nat. Rev. Mol. Cell Biol..

[5] Tam PPL, Behringer RR (1997). Mouse gastrulation: the formation of a mammalian body plan. Mech. Dev..

[6] Pijuan-Sala B (2019). A single-cell molecular map of mouse gastrulation and early organogenesis. Nature.

[7] Grosswendt S (2020). Epigenetic regulator function through mouse gastrulation. Nature.

[8] Peng G (2019). Molecular architecture of lineage allocation and tissue organization in early mouse embryo. Nature.

[9] Chan MM (2019). Molecular recording of mammalian embryogenesis. Nature.

[10] Cao J (2019). The single-cell transcriptional landscape of mammalian organogenesis. Nature.

[11] Scialdone A (2016). Resolving early mesoderm diversification through single-cell expression profiling. Nature.

[12] Ibarra-Soria X (2018). Defining murine organogenesis at single-cell resolution reveals a role for the leukotriene pathway in regulating blood progenitor formation. Nat. Cell Biol..

[13] Peng G (2016). Spatial transcriptome for the molecular annotation of lineage fates and cell identity in mid-gastrula mouse embryo. Dev. Cell.

[14] Mohammed H (2017). Single-cell landscape of transcriptional heterogeneity and cell fate decisions during mouse early gastrulation. Cell Rep..

[15] Raj B (2018). Simultaneous single-cell profiling of lineages and cell types in the vertebrate brain. Nat. Biotechnol..

[16] Alemany A, Florescu M, Baron CS, Peterson-Maduro J, Van Oudenaarden A (2018). Whole-organism clone tracing using single-cell sequencing. Nature.

[17] Spanjaard B (2018). Simultaneous lineage tracing and cell-type identification using CRISPR–Cas9-induced genetic scars. Nat. Biotechnol..

[18] Kalhor R (2018). Developmental barcoding of whole mouse via homing CRISPR. Science.

[19] van den Brink SC (2020). Single-cell and spatial transcriptomics reveal somitogenesis in gastruloids. Nature.

[20] Richardson L (2014). EMAGE mouse embryo spatial gene expression database: 2014 update. Nucleic Acids Res..

[21] Diez-Roux G (2011). A high-resolution anatomical atlas of the transcriptome in the mouse embryo. PLoS Biol..

[22] Moffitt JR (2016). High-throughput single-cell gene-expression profiling with multiplexed error-robust fluorescence in situ hybridization. Proc. Natl Acad. Sci. USA.

[23] Chen KH, Boettiger AN, Moffitt JR, Wang S, Zhuang X (2015). Spatially resolved, highly multiplexed RNA profiling in single cells. Science.

[24] Shah S, Lubeck E, Zhou W, Cai L (2016). In situ transcription profiling of single cells reveals spatial organization of cells in the mouse hippocampus. Neuron.

[25] Eng CHL (2019). Transcriptome-scale super-resolved imaging in tissues by RNA seqFISH. Nature.

[26] Shah S (2018). Dynamics and spatial genomics of the nascent transcriptome by intron seqFISH. Cell.

[27] Xia C, Fan J, Emanuel G, Hao J, Zhuang X (2019). Spatial transcriptome profiling by MERFISH reveals subcellular RNA compartmentalization and cell cycle-dependent gene expression. Proc. Natl Acad. Sci. USA.

[28] Wang X (2018). Three-dimensional intact-tissue sequencing of single-cell transcriptional states. Science.

[29] Chen X, Sun YC, Church GM, Lee JH, Zador AM (2018). Efficient in situ barcode sequencing using padlock probe-based BaristaSeq. Nucleic Acids Res..

[30] Ke R (2013). In situ sequencing for RNA analysis in preserved tissue and cells. Nat. Methods.

[31] Rodriques SG (2019). Slide-seq: a scalable technology for measuring genome-wide expression at high spatial resolution. Science.

[32] Ståhl PL (2016). Visualization and analysis of gene expression in tissue sections by spatial transcriptomics. Science.

[33] Chen F (2016). Nanoscale imaging of RNA with expansion microscopy. Nat. Methods.

[34] Tillberg PW (2016). Protein-retention expansion microscopy of cells and tissues labeled using standard fluorescent proteins and antibodies. Nat. Biotechnol..

[35] Berg S (2019). ilastik: interactive machine learning for (bio)image analysis. Nat. Methods.

[36] Weinert S, Bergmann N, Luo X, Erdmann B, Gotthardt M (2006). M line-deficient titin causes cardiac lethality through impaired maturation of the sarcomere. J. Cell Biol..

[37] Breher SS (2004). Popeye domain containing gene 2 (*Popdc2*) is a myocyte-specific differentiation marker during chick heart development. Dev. Dyn..

[38] McFadden DG (2005). The Hand1 and Hand2 transcription factors regulate expansion of the embryonic cardiac ventricles in a gene dosage-dependent manner. Development.

[39] Barnes RM, Firulli BA, Conway SJ, Vincentz JW, Firulli AB (2010). Analysis of the Hand1 cell lineage reveals novel contributions to cardiovascular, neural crest, extra-embryonic, and lateral mesoderm derivatives. Dev. Dyn..

[40] Reiter JF (1999). Gata5 is required for the development of the heart and endoderm in zebrafish. Genes Dev..

[41] Lagutin OV (2003). Six3 repression of Wnt signaling in the anterior neuroectoderm is essential for vertebrate forebrain development. Genes Dev..

[42] Porter FD (1997). *Lhx2*, a LIM homeobox gene, is required for eye, forebrain, and definitive erythrocyte development. Development.

[43] Simeone A, Acampora D, Gulisano M, Stornaiuolo A, Boncinelli E (1992). Nested expression domains of four homeobox genes in developing rostral brain. Nature.

[44] Matsuo I, Kuratani S, Kimura C, Takeda N, Aizawa S (1995). Mouse Otx2 functions in the formation and patterning of rostral head. Genes Dev..

[45] Ang SL (1996). A targeted mouse *Otx2* mutation leads to severe defects in gastrulation and formation of axial mesoderm and to deletion of rostral brain. Development.

[46] Suzuki N, Rohdewohld H, Neuman T, Gruss P, Schöler HR (1990). Oct-6: a POU transcription factor expressed in embryonal stem cells and in the developing brain. EMBO J..

[47] Zappone MV (2000). Sox2 regulatory sequences direct expression of a β-geo transgene to telencephalic neural stem cells and precursors of the mouse embryo, revealing regionalization of gene expression in CNS stem cells. Development.

[48] Wood HB, Episkopou V (1999). Comparative expression of the mouse *Sox1*, *Sox2* and *Sox3* genes from pre-gastrulation to early somite stages. Mech. Dev..

[49] Mahlapuu M, Ormestad M, Enerbäck S, Carlsson P (2001). The forkhead transcription factor Foxf1 is required for differentiation of extra-embryonic and lateral plate mesoderm. Development.

[50] Sasaki H, Hogan BLM (1993). Differential expression of multiple fork head related genes during gastrulation and axial pattern formation in the mouse embryo. Development.

[51] Tamplin OJ (2008). Microarray analysis of *Foxa2* mutant mouse embryos reveals novel gene expression and inductive roles for the gastrula organizer and its derivatives. BMC Genomics.

[52] Collins MM, Baumholtz AI, Ryan AK (2013). Claudin family members exhibit unique temporal and spatial expression boundaries in the chick embryo. Tissue Barriers.

[53] Deschamps J, van Nes J (2005). Developmental regulation of the Hox genes during axial morphogenesis in the mouse. Development.

[54] Kesseland M, Gruss P (1991). Homeotic transformations of murine vertebrae and concomitant alteration of *Hox* codes induced by retinoic acid. Cell.

[55] Haghverdi L, Lun ATL, Morgan MD, Marioni JC (2018). Batch effects in single-cell RNA-sequencing data are corrected by matching mutual nearest neighbors. Nat. Biotechnol..

[56] Hay ED (2005). The mesenchymal cell, its role in the embryo, and the remarkable signaling mechanisms that create it. Dev. Dyn..

[57] Rossi JM, Dunn NR, Hogan BLM, Zaret KS (2001). Distinct mesodermal signals, including BMPs from the septum, transversum mesenchyme, are required in combination for hepatogenesis from the endoderm. Genes Dev..

[58] Jung J, Zheng M, Goldfarb M, Zaret KS (1999). Initiation of mammalian liver development from endoderm by fibroblast growth factors. Science.

[59] Wu SP, Dong XR, Regan JN, Su C, Majesky MW (2013). *Tbx18* regulates development of the epicardium and coronary vessels. Dev. Biol..

[60] Cai CL (2008). A myocardial lineage derives from *Tbx18* epicardial cells. Nature.

[61] Guadix JA (2011). Wt1 controls retinoic acid signalling in embryonic epicardium through transcriptional activation of *Raldh2s*. Development.

[62] Ijpenberg A (2007). Wt1 and retinoic acid signaling are essential for stellate cell development and liver morphogenesis. Dev. Biol..

[63] Prummel KD, Nieuwenhuize S, Mosimann C (2020). The lateral plate mesoderm. Development.

[64] Andersson ER, Sandberg R, Lendahl U (2011). Notch signaling: simplicity in design, versatility in function. Development.

[65] Maretto S (2003). Mapping Wnt/β-catenin signaling during mouse development and in colorectal tumors. Proc. Natl Acad. Sci. USA.

[66] Depew MJ (1999). Dlx5 regulates regional development of the branchial arches and sensory capsules. Development.

[67] Biben C, Harvey RP (1997). Homeodomain factor Nkx2-5 controls left/right asymmetric expression of bHLH gene eHAND during murine heart development. Genes Dev..

[68] Lien CL (1999). Control of early cardiac-specific transcription of *Nkx2-5* by a GATA-dependent enhancer. Development.

[69] Oginuma M, Hirata T, Saga Y (2008). Identification of presomitic mesoderm (PSM)-specific *Mesp1* enhancer and generation of a PSM-specific Mesp1/Mesp2-null mouse using BAC-based rescue technology. Mech. Dev..

[70] Haraguchi S (2001). Transcriptional regulation of *Mesp1* and *Mesp2* genes: differential usage of enhancers during development. Mech. Dev..

[71] Okuda A (1998). UTF1, a novel transcriptional coactivator expressed in pluripotent embryonic stem cells and extra-embryonic cells. EMBO J..

[72] Wurst W, Bally-Cuif L, Bally-Cuif L (2001). Neural plate patterning: upstream and downstream of the isthmic organizer. Nat. Rev. Neurosci..

[73] Raible F, Brand M (2004). *Divide et Impera*—the midbrain–hindbrain boundary and its organizer. Trends Neurosci..

[74] Dworkin S, Jane SM (2013). Novel mechanisms that pattern and shape the midbrain–hindbrain boundary. Cell. Mol. Life Sci..

[75] Lowery LA, De Rienzo G, Gutzman JH, Sive H (2009). Characterization and classification of zebrafish brain morphology mutants. Anat. Rec..

[76] Millet S (1999). A role for *Gbx2* in repression of *Otx2* and positioning the mid/hindbrain organizer. Nature.

[77] Bouillet P, Chazaud C, Oulad‐Abdelghani M, Dollé P, Chambon P (1995). Sequence and expression pattern of the *Stra7* (*Gbx‐2*) homeobox‐containing gene induced by retinoic acid in P19 embryonal carcinoma cells. Dev. Dyn..

[78] Haghverdi L, Büttner M, Wolf FA, Buettner F, Theis FJ (2016). Diffusion pseudotime robustly reconstructs lineage branching. Nat. Methods.

[79] La Manno G (2016). Molecular diversity of midbrain development in mouse, human, and stem cells. Cell.

[80] Blake JA, Ziman MR (2014). Pax genes: regulators of lineage specification and progenitor cell maintenance. Development.

[81] Ghazanfar S (2020). Investigating higher-order interactions in single-cell data with scHOT. Nat. Methods.

[82] Sunmonu NA, Li K, Guo Q, Li JYH (2011). *Gbx2* and *Fgf8* are sequentially required for formation of the midbrain–hindbrain compartment boundary. Development.

[83] Chung S (2009). Wnt1–lmx1a forms a novel autoregulatory loop and controls midbrain dopaminergic differentiation synergistically with the SHH–FoxA2 pathway. Cell Stem Cell.

[84] Hidalgo-Sánchez M, Millet S, Simeone A, Alvarado-Mallart RM (1999). Comparative analysis of *Otx2*, *Gbx2*, *Pax2*, *Fgf8* and *Wnt1* gene expressions during the formation of the chick midbrain/hindbrain domain. Mech. Dev..

[85] Wurst W, Auerbach AB, Joyner AL (1994). Multiple developmental defects in Engrailed-1 mutant mice: an early mid-hindbrain deletion and patterning defects in forelimbs and sternum. Development.

[86] Wittmann DM (2009). Spatial analysis of expression patterns predicts genetic interactions at the mid-hindbrain boundary. PLoS Comput. Biol..

[87] Davis CA, Noble-Topham SE, Rossant J, Joyner AL (1988). Expression of the homeo box-containing gene *En-2* delineates a specific region of the developing mouse brain. Genes Dev..

[88] McMahon AP, Joyner AL, Bradley A, McMahon JA (1992). The midbrain–hindbrain phenotype of Wnt-1–Wnt-1^–^ mice results from stepwise deletion of engrailed-expressing cells by 9.5 days postcoitum. Cell.

[89] McMahon AP, Bradley A (1990). The *Wnt*-1 (*int*-1) proto-oncogene is required for development of a large region of the mouse brain. Cell.

[90] Bayly RD, Brown CY, Agarwala S (2012). A novel role for FOXA2 and SHH in organizing midbrain signaling centers. Dev. Biol..

[91] Shimeld SM, McKay IJ, Sharpe PT (1996). The murine homeobox gene *Msx-3* shows highly restricted expression in the developing neural tube. Mech. Dev..

[92] Kesavan G, Machate A, Hans S, Brand M (2020). Cell-fate plasticity, adhesion and cell sorting complementarily establish a sharp midbrain–hindbrain boundary. Development.

[93] Kuwahara A (2020). Delineating the early transcriptional specification of the mammalian trachea and esophagus. eLife.

[94] Kim E (2019). Isl1 regulation of Nkx2.1 in the early foregut epithelium is required for trachea–esophageal separation and lung lobation. Dev. Cell.

[95] Que J (2007). Multiple dose-dependent roles for Sox2 in the patterning and differentiation of anterior foregut endoderm. Development.

[96] Que J (2015). The initial establishment and epithelial morphogenesis of the esophagus: a new model of tracheal–esophageal separation and transition of simple columnar into stratified squamous epithelium in the developing esophagus. Wiley Interdiscip. Rev. Dev. Biol..

[97] Piccolo S, Sasai Y, Lu B, De Robertis EM (1996). Dorsoventral patterning in *Xenopus*: inhibition of ventral signals by direct binding of chordin to BMP-4. Cell.

[98] Han L (2017). Osr1 functions downstream of Hedgehog pathway to regulate foregut development. Dev. Biol..

[99] Chapman DL (1996). Expression of the T-box family genes, *Tbx1*–*Tbx5*, during early mouse development. Dev. Dyn..

[100] McLaughlin D, Murphy P, Puri P (2014). Altered *Tbx1* gene expression is associated with abnormal oesophageal development in the adriamycin mouse model of oesophageal atresia/tracheo-oesophageal fistula. Pediatr. Surg. Int..

[101] Gopalakrishnan S, Comai G, Sambasivan R, Francou A, Kelly RG (2015). A cranial mesoderm origin for esophagus striated muscles. Dev. Cell.

[102] Nasr T (2019). Endosome-mediated epithelial remodeling downstream of Hedgehog–Gli is required for tracheoesophageal separation. Dev. Cell.

[103] Domyan ET (2011). Signaling through BMP receptors promotes respiratory identity in the foregut via repression of *Sox2*. Development.

[104] Goss AM (2009). Wnt2/2b and β-catenin signaling are necessary and sufficient to specify lung progenitors in the foregut. Dev. Cell.

[105] Que J, Choi M, Ziel JW, Klingensmith J, Hogan BLM (2006). Morphogenesis of the trachea and esophagus: current players and new roles for noggin and Bmps. Differentiation.

[106] Askary A (2020). In situ readout of DNA barcodes and single base edits facilitated by in vitro transcription. Nat. Biotechnol..

[107] Chow, K.-H. K. et al. Imaging cell lineage with a synthetic digital recording system. *Science* **372**, eabb3099 (2021). 10.1126/science.abb3099 33833095

[108] Lun ATL, McCarthy DJ, Marioni JC (2016). A step-by-step workflow for low-level analysis of single-cell RNA-seq data with Bioconductor. F1000Res..

[109] Eng CHL, Shah S, Thomassie J, Cai L (2017). Profiling the transcriptome with RNA SPOTs. Nat. Methods.

[110] Beliveau BJ (2012). Versatile design and synthesis platform for visualizing genomes with Oligopaint FISH probes. Proc. Natl Acad. Sci. USA.

[111] Takei Y, Shah S, Harvey S, Qi LS, Cai L (2017). Multiplexed dynamic imaging of genomic loci by combined CRISPR imaging and DNA sequential FISH. Biophys. J..

[112] Wang G, Moffitt JR, Zhuang X (2018). Multiplexed imaging of high-density libraries of RNAs with MERFISH and expansion microscopy. Sci. Rep..

[113] Edelstein A, Amodaj N, Hoover K, Vale R, Stuurman N (2010). Computer control of microscopes using µmanager. Curr. Protoc. Mol. Biol..

[114] Beier T (2017). Multicut brings automated neurite segmentation closer to human performance. Nat. Methods.

[115] Choi HMT (2018). Third-generation in situ hybridization chain reaction: multiplexed, quantitative, sensitive, versatile, robust. Development.

[116] Anderson, M., Magidson, V., Kageyama, R. & Lewandoski, M. *Fgf4* maintains *Hes7* levels critical for normal somite segmentation clock function. *eLife* **9**, e55608 (2020). 10.7554/eLife.55608 PMC7717904 33210601

[117] Trivedi V, Choi HMT, Fraser SE, Pierce NA (2018). Multidimensional quantitative analysis of mRNA expression within intact vertebrate embryos. Development.

[118] Guibentif C (2021). Diverse routes toward early somites in the mouse embryo. Dev. Cell.

[119] Ashburner M (2000). Gene Ontology: tool for the unification of biology. Nat. Genet..

[120] Langfelder P, Zhang B, Horvath S (2008). Defining clusters from a hierarchical cluster tree: the dynamic tree cut package for R. Bioinformatics.

[121] Hastie T, Stuetzle W (1989). Principal curves. J. Am. Stat. Assoc..

