# Supplementary material for: Integration of spatial and single-cell transcriptomic data elucidates mouse organogenesis
Source: Nat Biotechnol. 2021 Sep 6;40(1):74–85. doi: 10.1038/s41587-021-01006-2 (PMC8763645; doi:10.1038/s41587-021-01006-2)
Supplement: Supplementary file 1 — Supplementary Figs. 1–15 and legend for Supplementary Tables 1–9. [file 41587_2021_1006_MOESM1_ESM.pdf]

nature portfolio

https://doi.org/10.1038/s41587-021-01006-2

Supplementary information

Integration of spatial and single-cell transcriptomic data elucidates mouse organogenesis

In the format provided by the authors and unedited

Supplementary Tables Legends

Supplementary Table 1: Primary probes. Each entry contains a primary probe sequence or a primary probe sequence combined with readout probe sequences.
(A) List of primary probes for seqFISH library.
(B) List of primary probes for non-barcoded sequential smFISH genes.
(C) Eef2 probeset A and B.

Supplementary Table 2: Readout probes and decoding strategy for seqFISH probe library.
(A) List of readout probe sequences and the corresponding fluorophore conjugated to the probes used in the seqFISH experiment.
(B) A list assigning a unique combination of four pseudocolors.
(C) The corresponding readout probe sequence to each gene of the seqFISH library is included; this allows decoding of the barcodes over the multiple imaging rounds.

Supplementary Table 3: Readout probes for non-barcoded sequential smFISH genes List of readout probe sequences and the corresponding fluorophore used for each gene measured by non-barcoded sequential smFISH.

Supplementary Table 4: List of spatial heterogeneity test results for each of the assigned cell types. Columns correspond to gene name, proportion of variability explained by neighboring genes' expression, P-value, t-statistics, and FDR-adjusted P-value. Spatial heterogeneity tests result from one-sided t-tests extracted from a linear model set-up.

Supplementary Table 5: List of spatial heterogeneity test results for each of the Forebrain/Midbrain/Hindbrain subclusters Columns correspond to gene name, proportion of variability explained by neighboring genes' expression, P-value, t-statistics, and FDR-adjusted P-value. Spatial heterogeneity tests result from one-sided t-tests extracted from a linear model set-up.

Supplementary Table 6: Imputation confidence scores per gene. Table showing the median concordance for each gene across all embryos and z-slices between observed and imputed gene expression values. Genes are ranked such that those with the highest values corresponding to genes that are very well predicted, and those with the lowest values are poorly predicted.

Supplementary Table 7: List of significantly differentially expressed genes between virtually dissected midbrain and hindbrain regions of embryo 2. Columns correspond to gene, FDR-adjusted P-value, log-fold change for midbrain, mean expression and direction of significance. Differential expression testing was performed using two-sample t-tests.

Supplementary Table 8: Top 500 spatially variable genes in the virtually dissected midbrain/hindbrain region of embryo 2. Columns correspond to gene, mean gene expression across all cells, scHOT weighted mean test statistic, FDR-adjusted P-value, significance ranking, gene cluster cutting hierarchical clustering tree for 25 clusters, and for 10 clusters.

Supplementary Table 9: List of differentially expressed genes between Lung1 and Lung2 subcluster. Columns correspond to gene name, P-value, FDR-adjusted P-value, log-fold change for Lung 1 / Lung 2, mean expression of cells in Lung 1 group, and in Lung 2 group. Differential expression testing was performed using two-sample t-tests.

Supplementary Figures

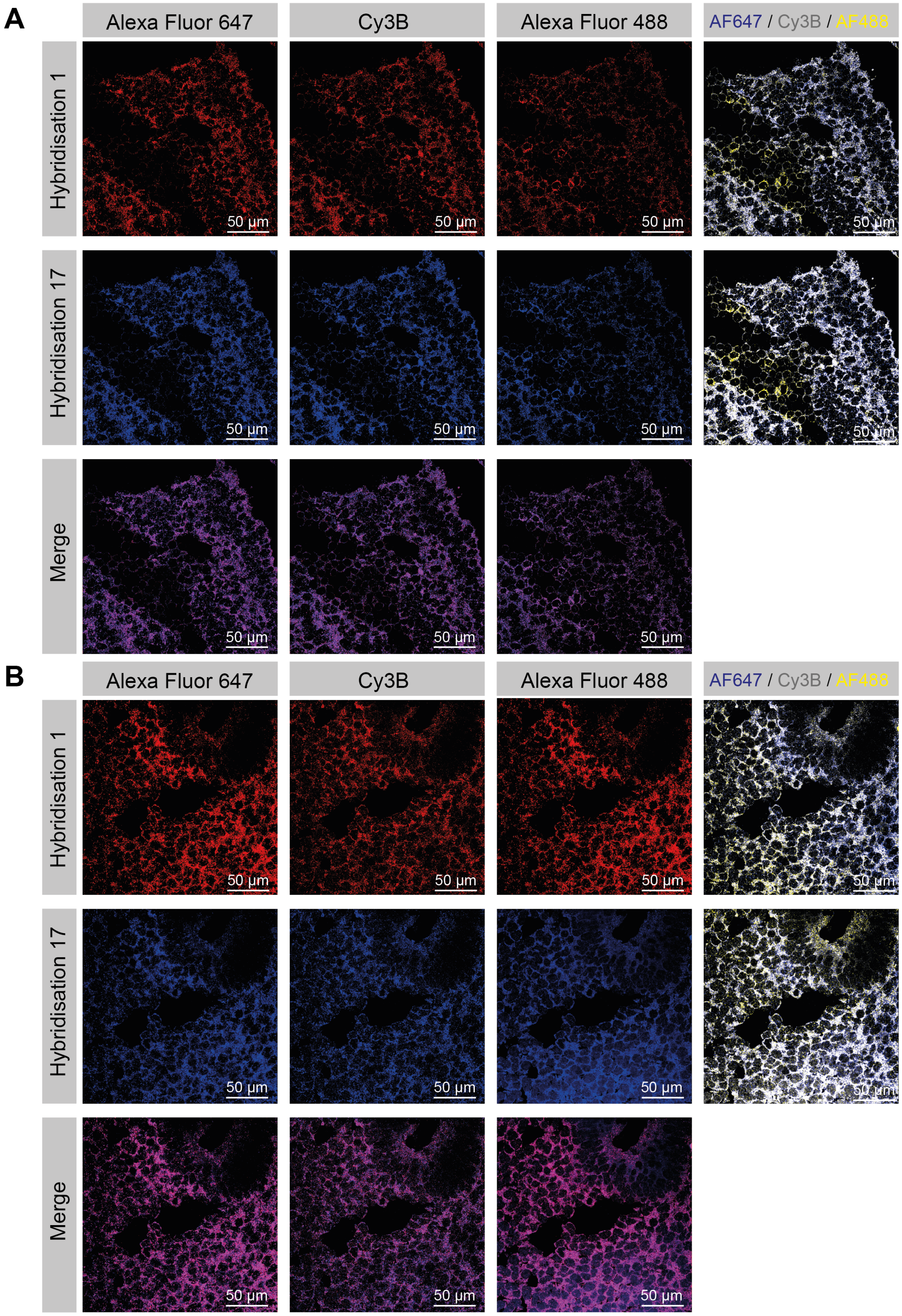

Supplementary Figure 1: Comparison of the hybridization round 1 and 17, an additional repeat of hybridization round 1, for quality control. Images are representative and were repeated independently for all N = 3 embryos with similar results(A) Visualization of the experimental block 1, containing embryos 1 and 2, and the experimental block 2 (B), containing embryo 3. mRNA spots for the Alexa fluor 647, Cy3B and Alexa fluor 488 channels are shown separately for hybridization round 1 (red) and 17 (blue). Strong overlap (purple) of the mRNA spots between hybridization rounds 1 and 17 suggests high RNA quality after the seqFISH imaging. A composite of all three channels is visualized for both hybridization rounds separately.

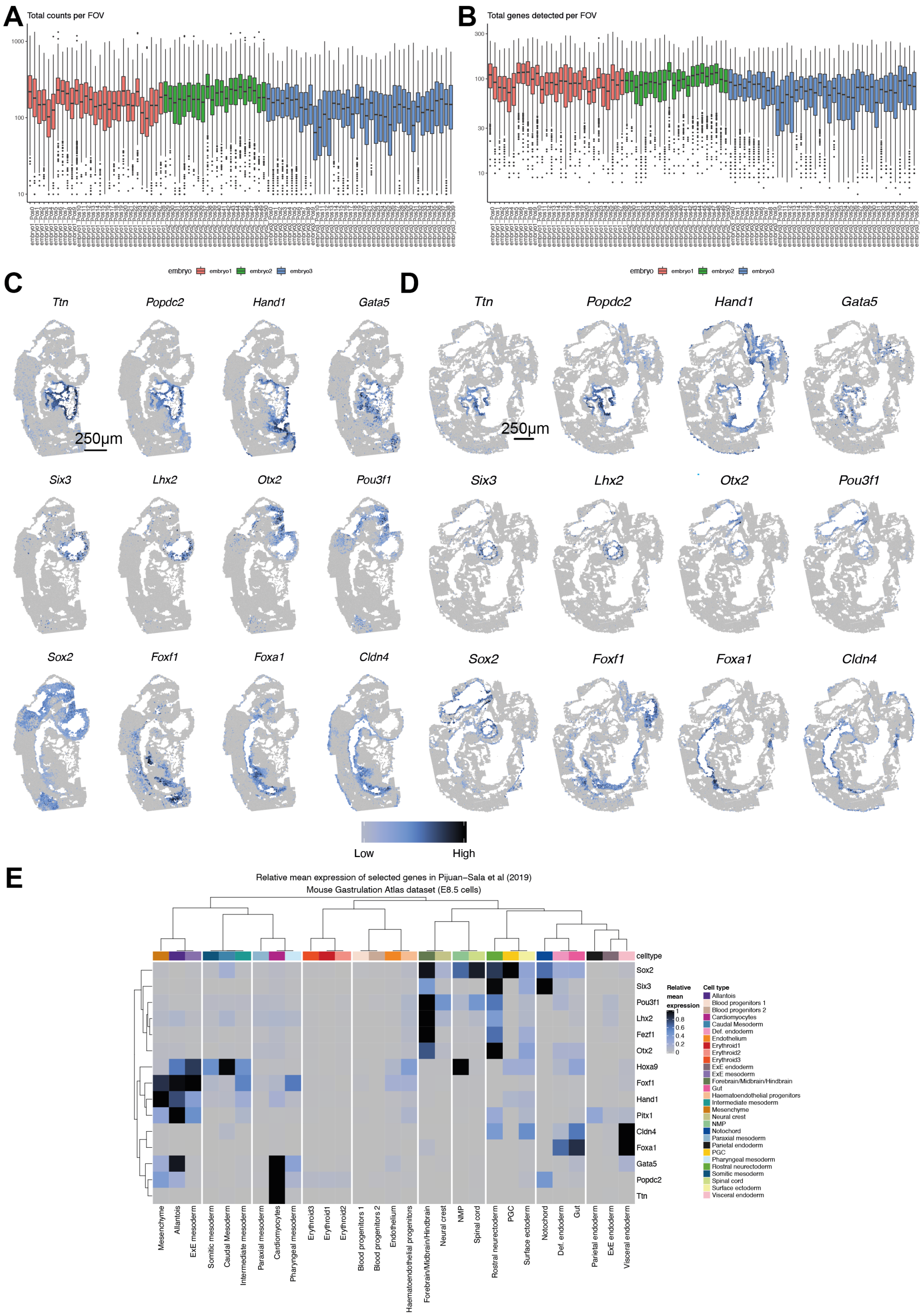

Supplementary Figure 2: Quality Control of seqFISH data.
(A) Boxplots of total mRNA molecules detected for each cell within each field of view (log10 scale), colored by embryo. N = 57,536 cells over 3 biologically independent embryos. Boxes display 25th, 50th, and 75th percentiles, and whiskers extend to closest observation within the outlier range, defined as not more than 1.5 times the interquartile range.
(B) Boxplots displaying total number of genes detected for each cell within each field of view (log10 scale), colored by embryo. N = 57,536 cells over 3 biologically independent embryos.
(C) Spatial expression of selected genes shown in Figure 1 for embryo 2. Scale bar 250 µm.
(D) as in C for embryo 3. Scale bar 250 µm.
(E) Heatmap of relative mean expression of cell types corresponding to E8.5 Gastrulation atlas data, cell type dendrogram corresponds to clustering using all data.

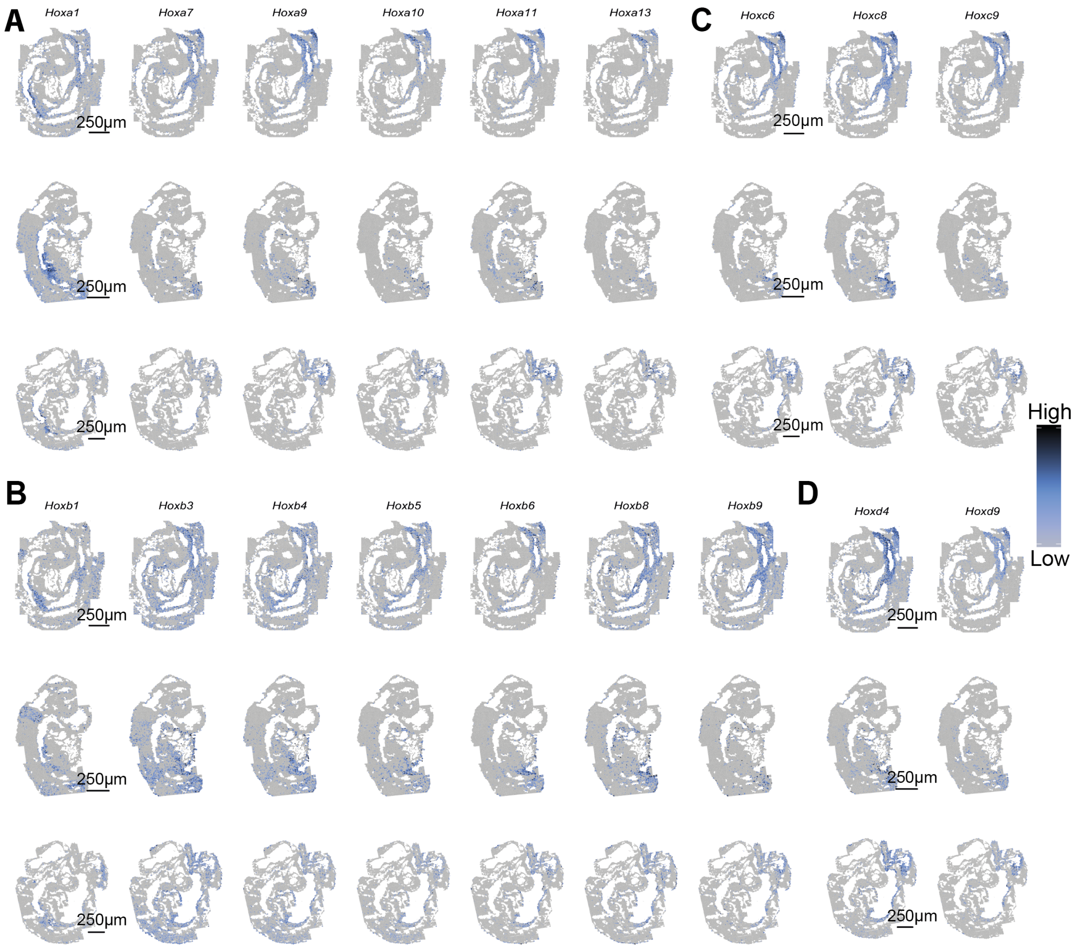

Supplementary Figure 3: Spatial Hox expression profiles to assess data quality.
(A) Spatial expression of HoxA family genes, ordered numerically, with each embryo per row. Scale bar 250 µm.
(B) as in A, with HoxB subfamily.
(C) as in A with HoxC subfamily.
(D) as in A with HoxD subfamily.

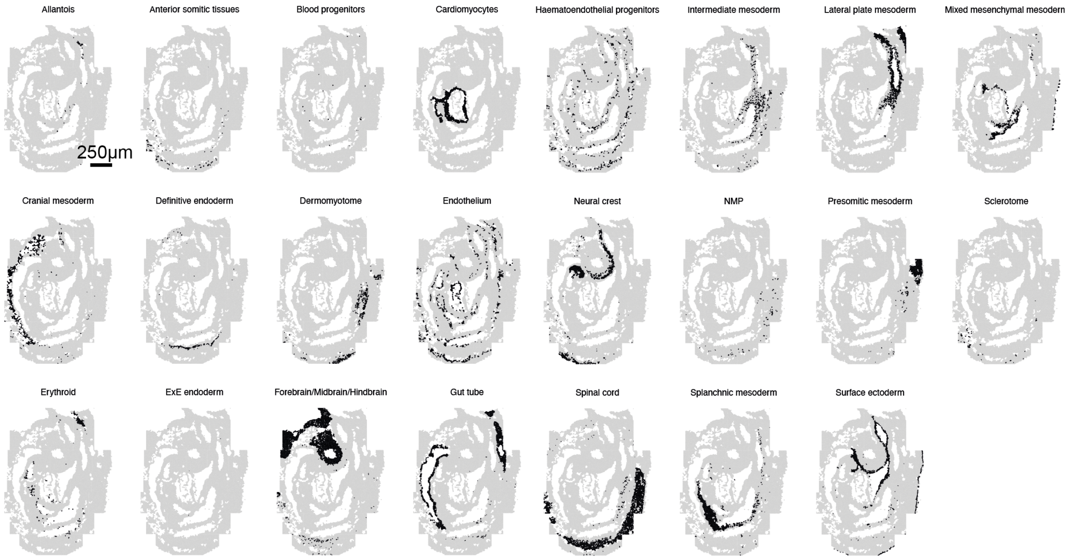

Supplementary Figure 4: Cell type annotation for Embryo 1
Spatial plots of embryo 1 where, for each panel, the selected cell type is shown in black.

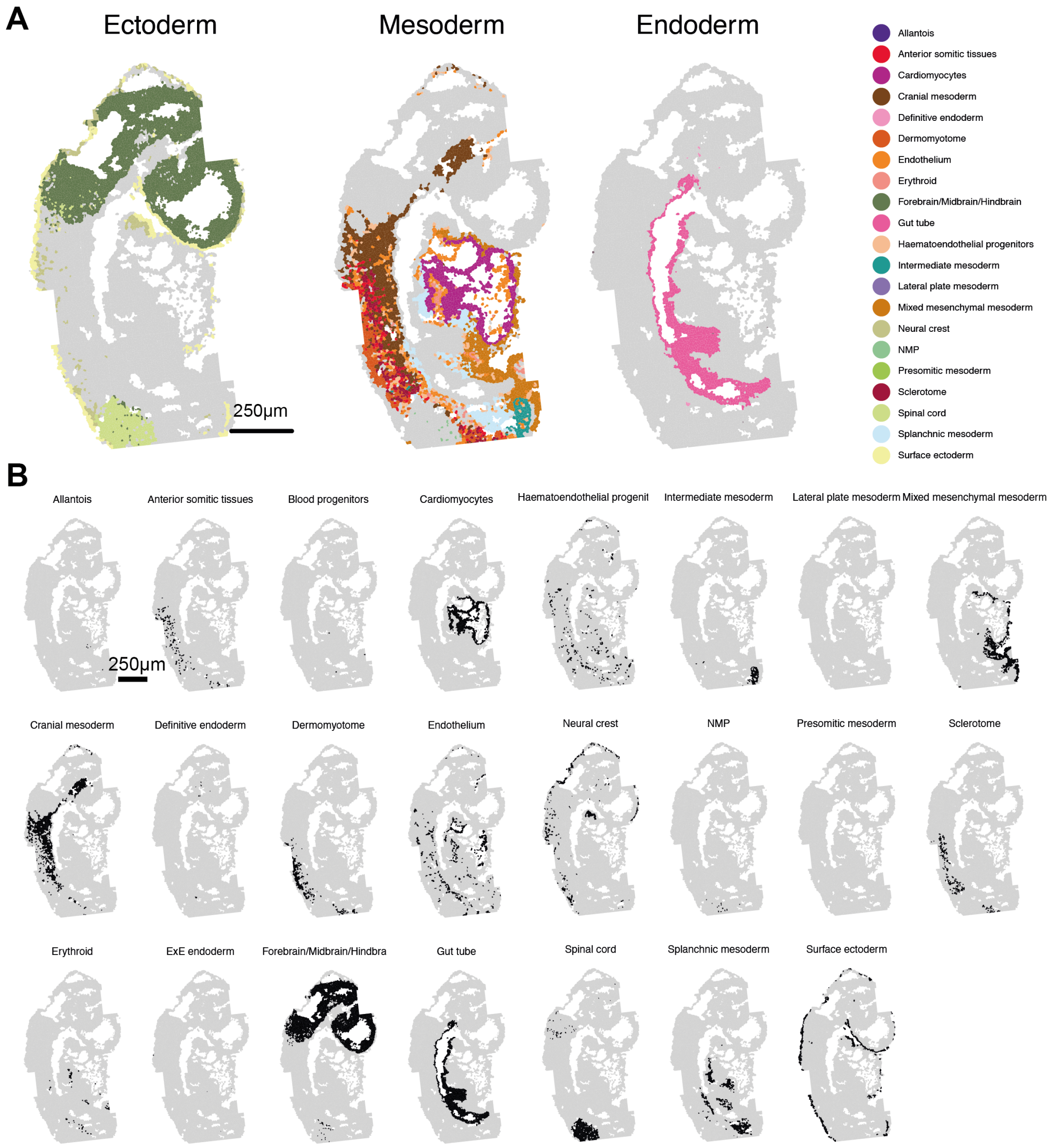

Supplementary Figure 5: Cell type annotation for Embryo 2

(A) Cell type maps separated by the three germ layers (ectoderm, mesoderm, endoderm) for embryo 2. Scale bars 250 µm.

(B) Spatial plots of embryo 2 where, for each panel, the selected cell type is shown in black.

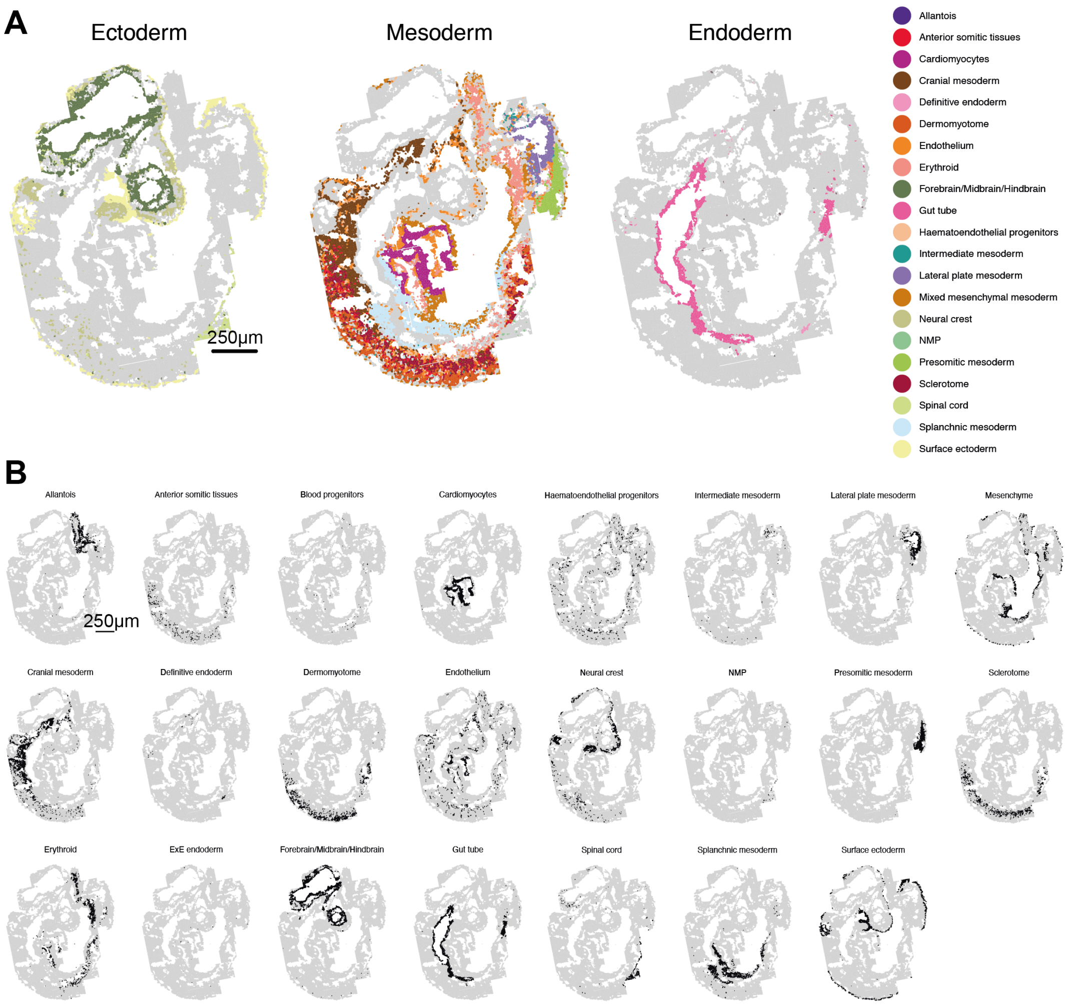

Supplementary Figure 6: Cell type annotation for Embryo 3

(A) Cell type maps separated by the three germ layers (ectoderm, mesoderm, endoderm) for embryo 3. Scale bars 250 µm.

(B) Spatial plots of embryo 3 where, for each panel, the selected cell type is shown in black.

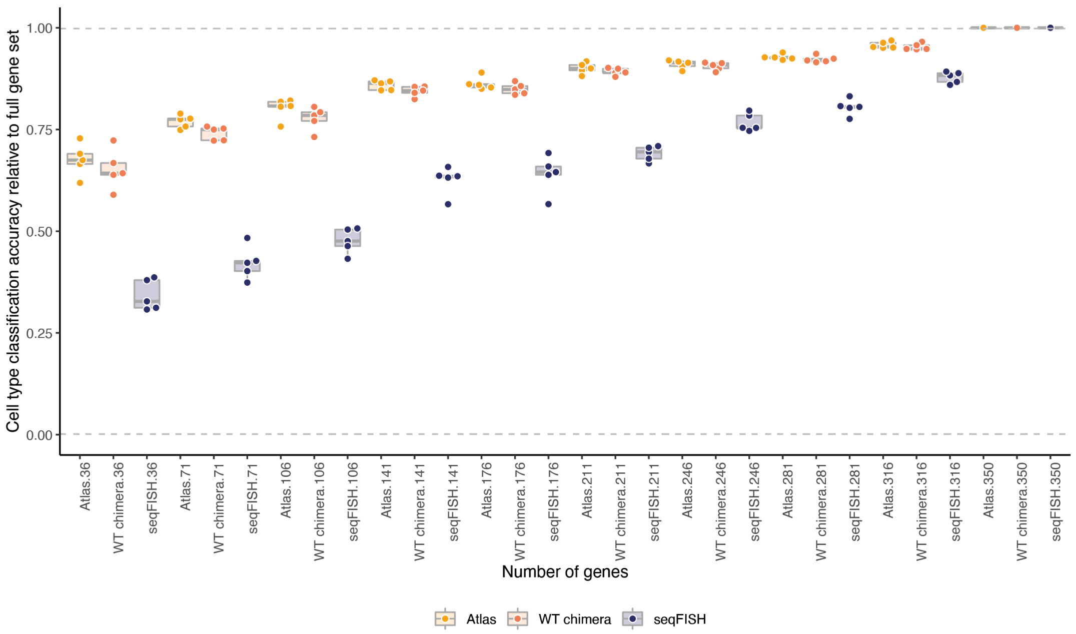

Supplementary Figure 7: Cell type classification accuracy with fewer genes Boxplots displaying the cell type classification accuracy relative to the full gene set (y-axis), according to data source (individual boxplots), i.e. Gastrulation Atlas, wild-type cells stemming from the WT/WT chimera at E8.5, and the seqFISH study data, and according to the number of genes randomly selected for joint integration and cell type identification.

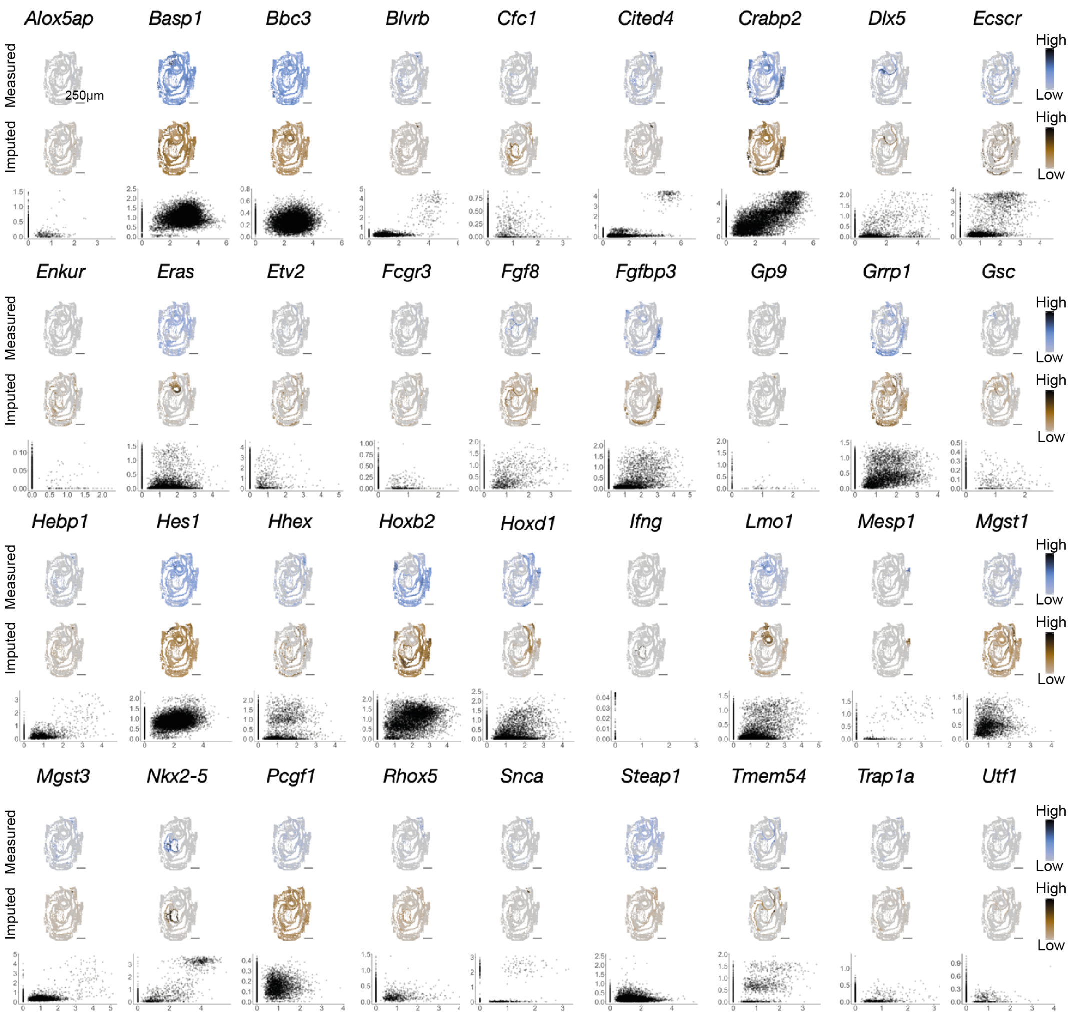

Supplementary Figure 8: Comparison between imputed expression counts and measured expression counts in embryo 1.1 for 36 genes measured with smFISH.
Each sub-panel corresponds to a single gene (denoted at the top). Upper sub-panels correspond to spatial distribution of measured logcounts (color gradient is specific to each gene). The Middle sub-panels show spatial expression maps of imputed logcounts (color gradient is specific to each gene). In the lower sub-panels, scatterplots show the measured logcounts (x-axis) and imputed logcounts (y-axis), where each point corresponds to a cell. Scale bars 250um.

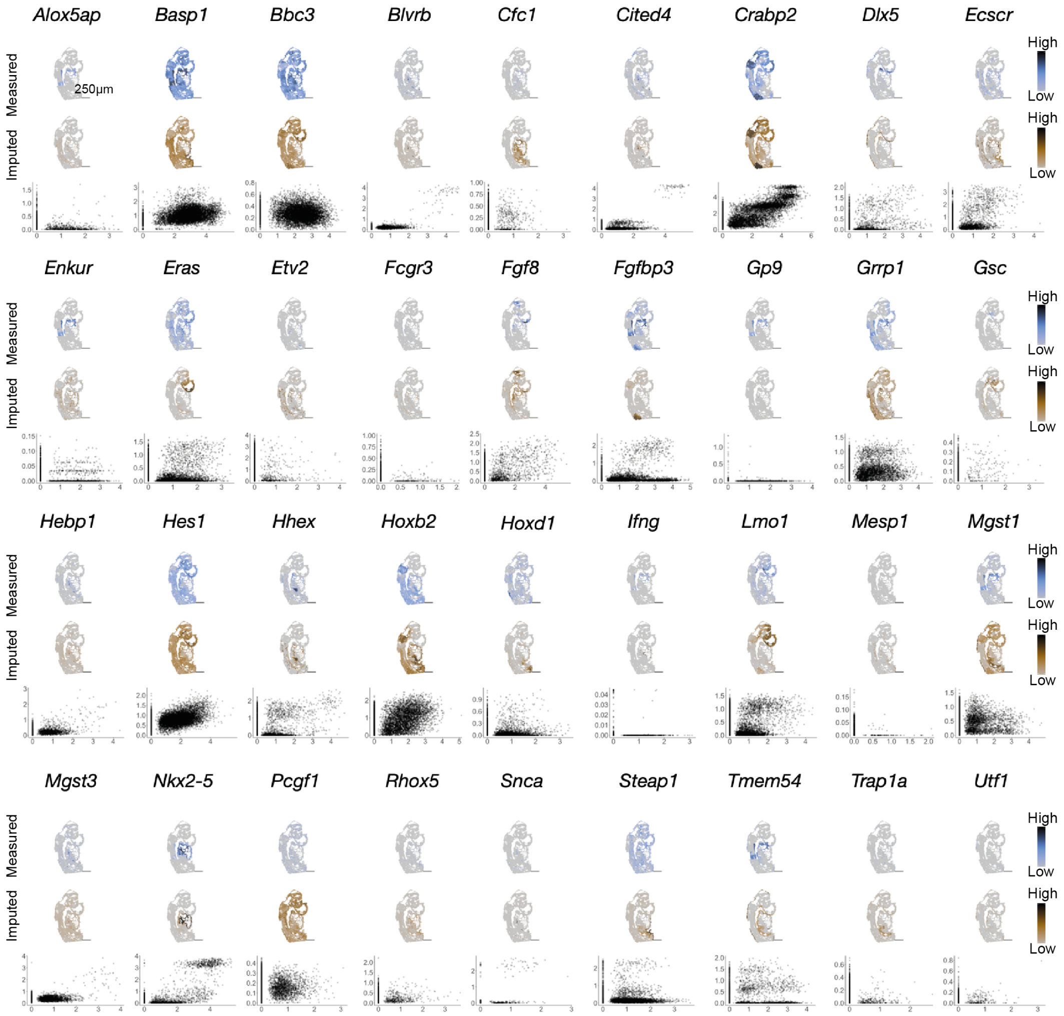

Supplementary Figure 9: Comparison between imputed expression counts and measured expression counts in embryo 2.1 for 36 genes measured with smFISH.
Each sub-panel corresponds to a single gene (denoted at the top). Upper sub-panels correspond to spatial distribution of measured logcounts (color gradient is specific to each gene). The Middle sub-panels show spatial expression maps of imputed logcounts (color gradient is specific to each gene). In the lower sub-panels, scatterplots show the measured logcounts (x-axis) and imputed logcounts (y-axis), where each point corresponds to a cell. Scale bars 250um.

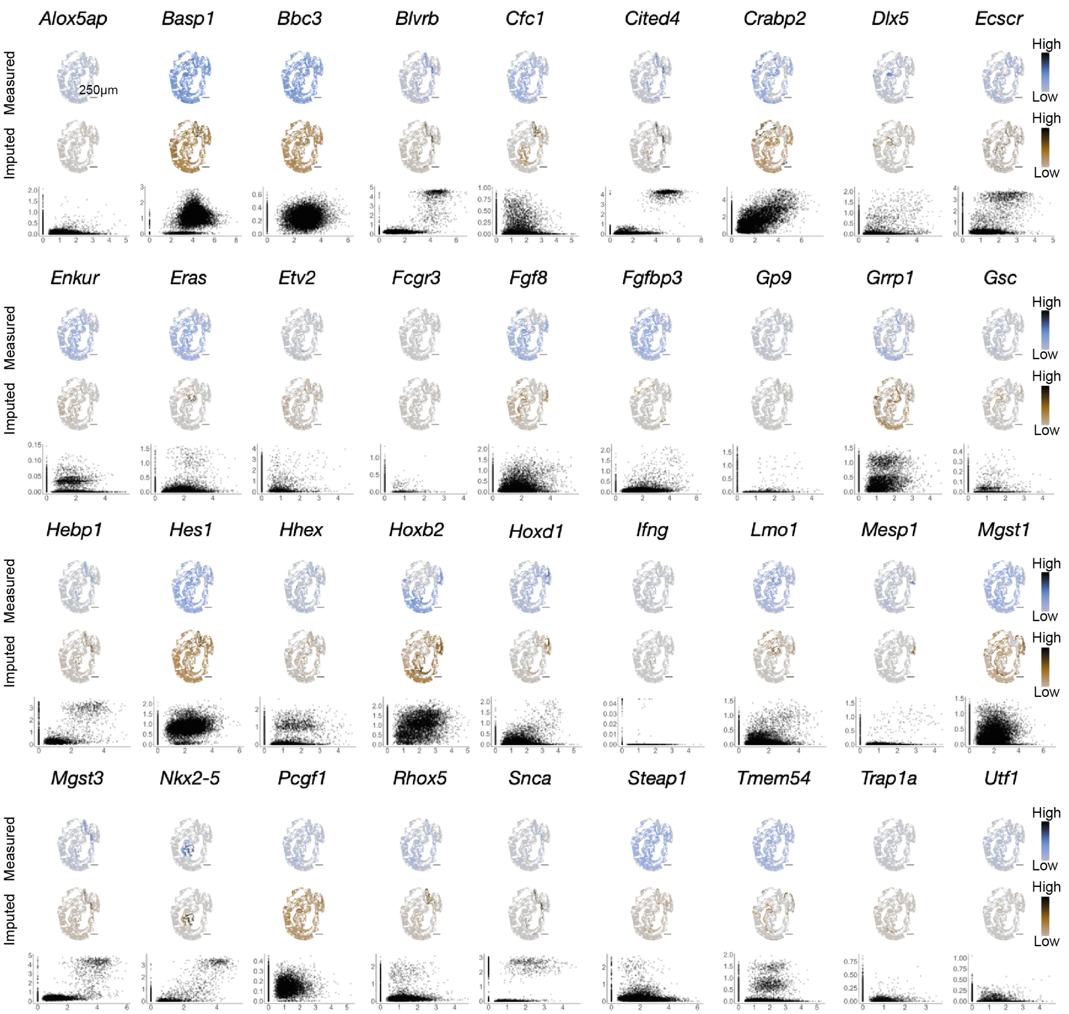

Supplementary Figure 10: Comparison between imputed expression counts and measured expression counts in embryo 3.1 for 36 genes measured with non-barcoded smFISH. Each sub-panel corresponds to a single gene (denoted at the top). Upper sub-panels correspond to spatial distribution of measured logcounts (color gradient is specific to each gene). The Middle sub-panels show spatial expression maps of imputed logcounts (color gradient is specific to each gene). In the lower sub-panels, scatterplots show the measured logcounts (x-axis) and imputed logcounts (y-axis), where each point corresponds to a cell. Scale bars 250um.

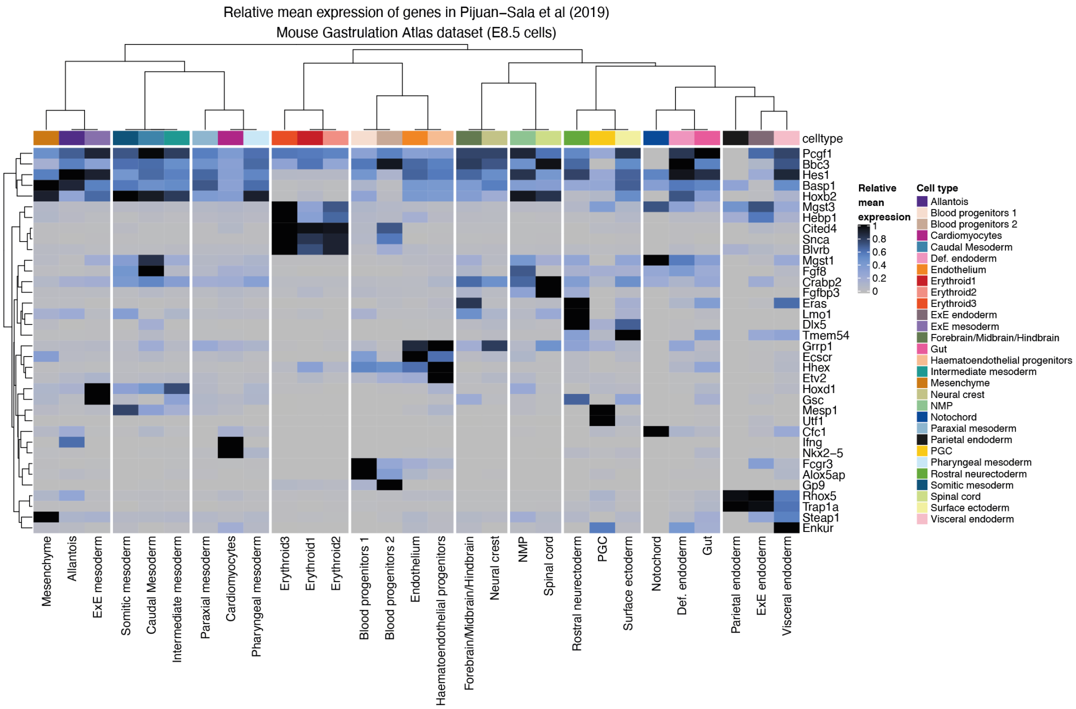

Supplementary Figure 11: Expression of smFISH genes in the Gastrulation atlas
Heatmap of relative mean expression of cell types for genes measured with non-barcoded sequential smFISH using the E8.5 Gastrulation atlas data. The cell type dendrogram was generated by clustering all data.

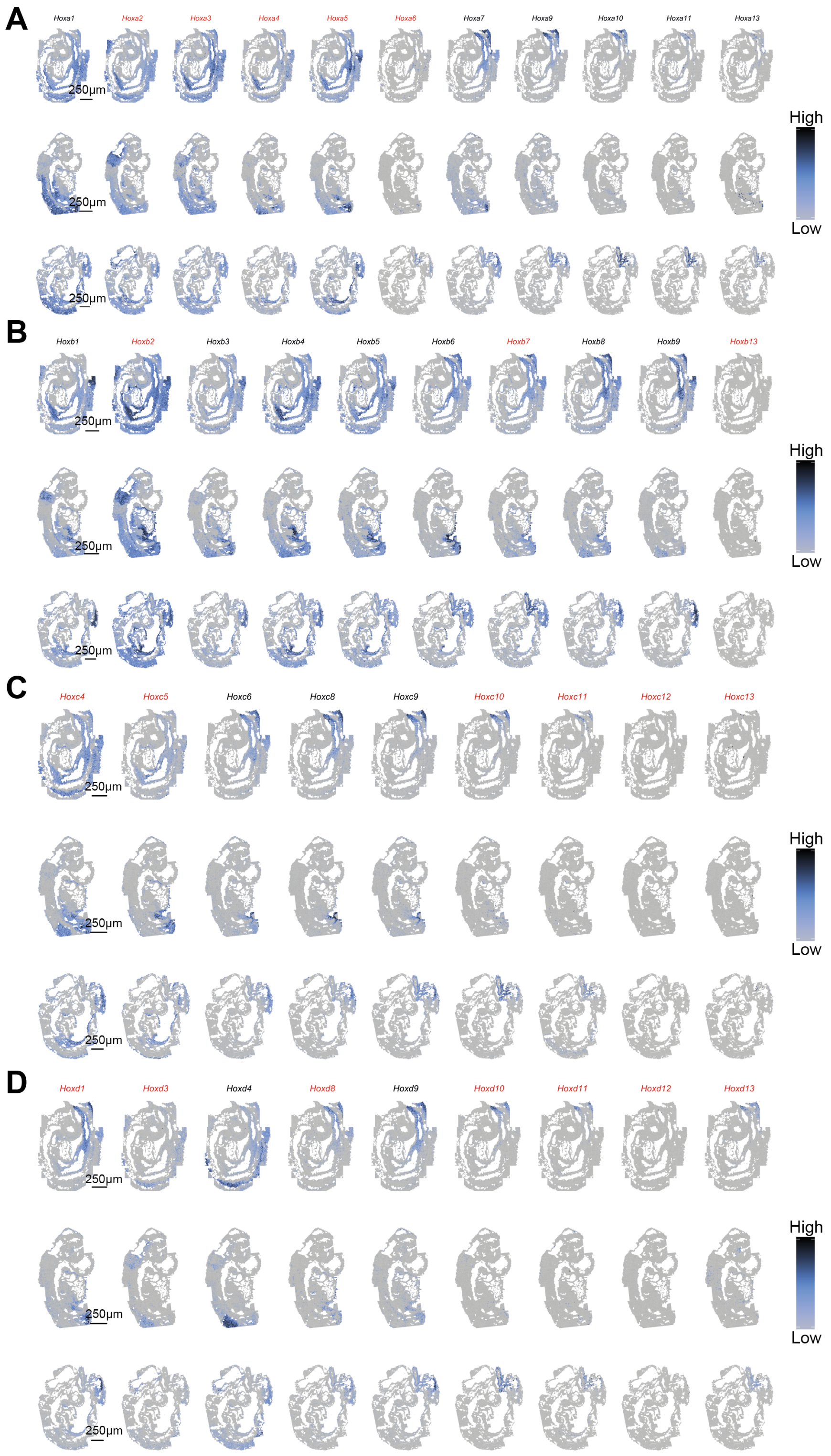

Supplementary Figure 12: Spatial expression of imputed Hox gene family.
(A) Spatial expression of all imputed HoxA family genes, ordered numerically, with each embryo per row. Gene name in red indicates whether the gene is absent from the seqFISH gene library. Scale bar 250 µm.
(B) as in A, with HoxB subfamily.
(C) as in A with HoxC subfamily.
(D) as in A with HoxD subfamily.

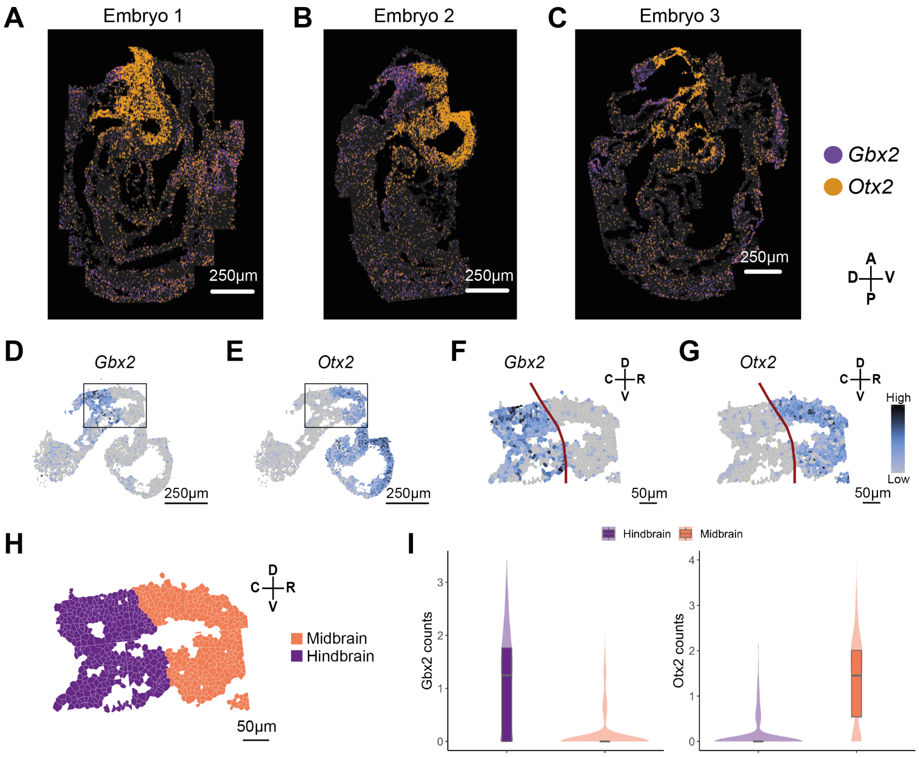

Supplementary Figure 13: Virtual dissection of Midbrain-Hindbrain Boundary.
(A) 'Digital in situ' showing detected mRNA molecules for Gbx2 (purple) and Otx2 (orange) across embryo 1. Scale bar 250 µm.
(B) as in A for embryo 2.
(C) as in A for embryo 3.
(D) Spatial expression of Gbx2 in the brain. Black rectangle corresponds to the virtually dissected region in which we predict the Midbrain-Hindbrain boundary (MHB) forms. Scale bar 250 µm.
(E) as in D for the gene Otx2. (F) Spatial expression of Gbx2 in the boxed region with corresponding virtual dissection (red line). Scale bar 250 µm.
(G) as in F for gene Otx2.
(H) Spatial distribution in the boxed region where cells are colored based on whether they are assigned a Midbrain (orange) or Hindbrain (purple) identity.
(I) Quantitative distribution of Otx2 and Gbx2 expression counts in the selected Midbrain and Hindbrain regions.

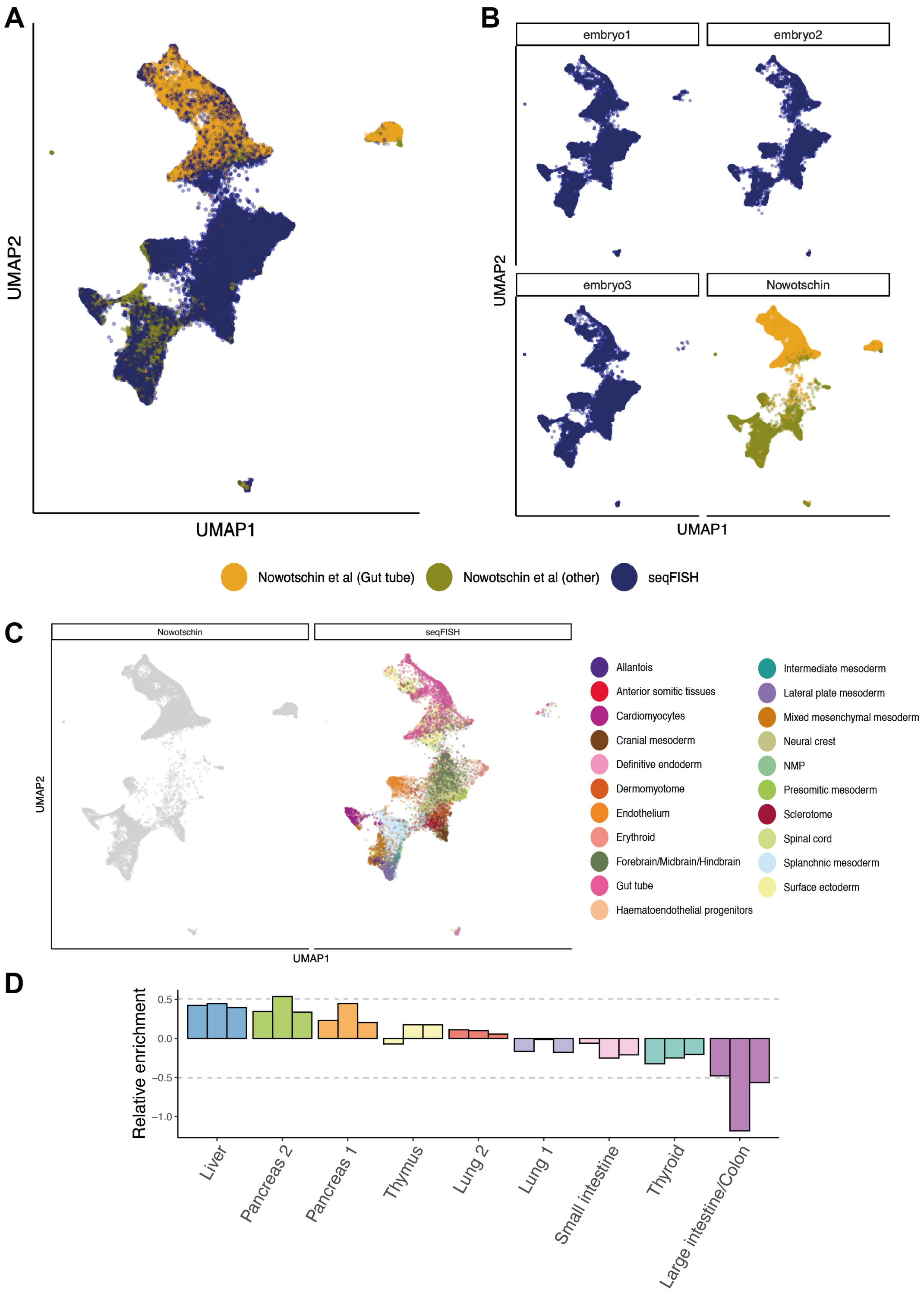

Supplementary Figure 14: Integration with Nowotschin et al. data.
(A) Joint UMAP of Nowotschin et al. and seqFISH expression data, with cells colored by dataset, and for the Nowotschin et al. dataset, whether the cell has an associated developing gut tube cell annotation.
(B) Joint UMAP of Nowotschin et al. and seqFISH expression data, with panels corresponding to each embryo and the Nowotschin et al. dataset. Colors as in A.
(C) Joint UMAP of Nowotschin et al. and seqFISH expression data, where seqFISH cells are colored by their refined cell type annotations based on integration with the Gastrulation atlas dataset.
(D) Barplot of relative enrichment in abundance of seqFISH cells compared to Nowotschin et al. cells, each bar corresponds to embryo 1, 2, and 3, from left to right.

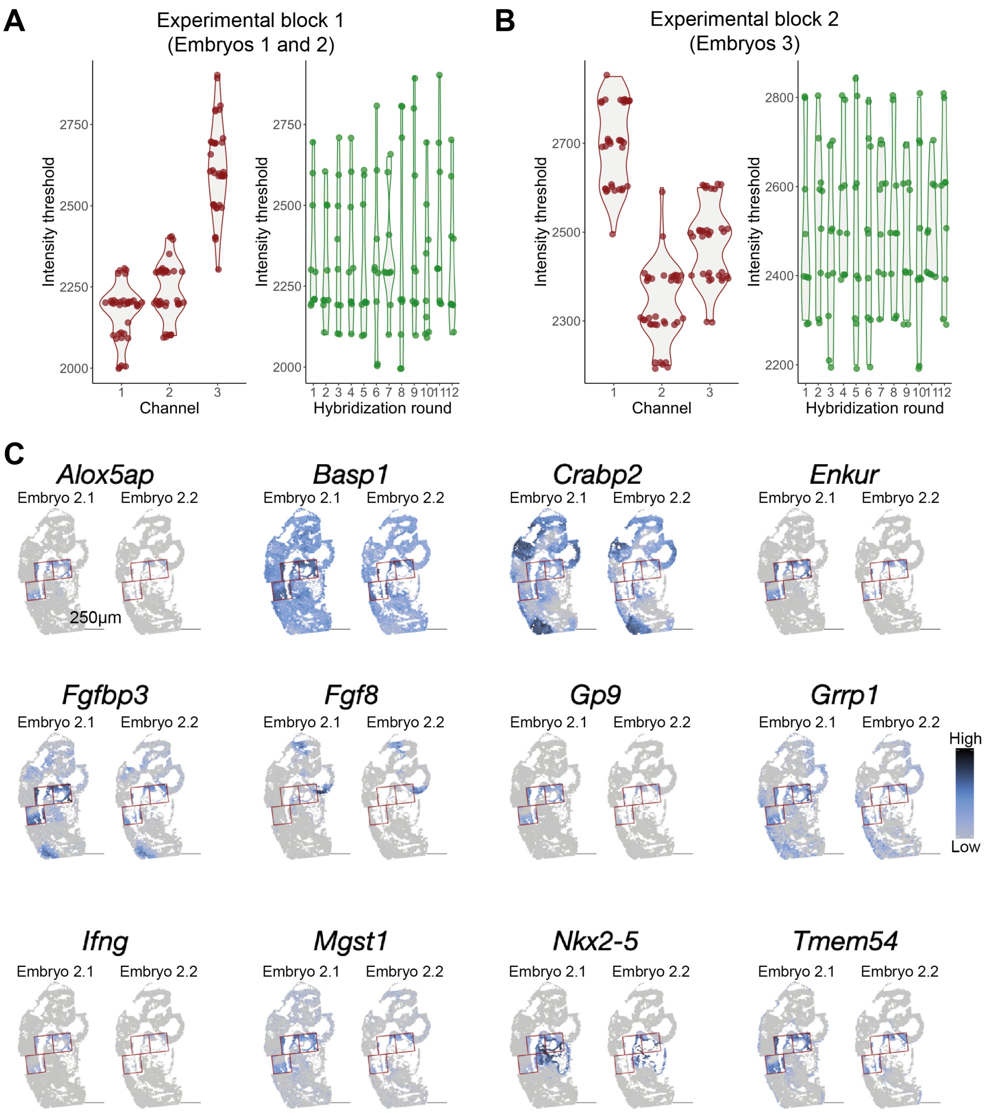

Supplementary Figure 15: Channel effect on the distribution of background noise for non-barcoded smFISH data.

(A) The intensity threshold that separates background spots is highly dependent on the channel (fluorescence) a gene was probed with, but not on the hybridization round. Violin plots on the left show intensity thresholds for experimental block 1 for each gene and field of view combination grouped by color channel, while violin plots on the right show intensity thresholds for each gene and field of view combination grouped by hybridization round.

(B) as in A for experimental block 2 (embryo 3).

(C) Spatial maps of non-barcoded smFISH genes that were probed with Alexa Fluor 647 (AF647) for embryo 2, with red squares around fields of view 39, 40, and 44, which display a strong field of view effect, regardless of the choice of intensity threshold.
