# Supplementary material for: Integration of spatial and single-cell transcriptomic data elucidates mouse organogenesis
Source: Nat Biotechnol. 2021 Sep 6;40(1):74–85. doi: 10.1038/s41587-021-01006-2 (PMC8763645; doi:10.1038/s41587-021-01006-2)
Supplement: Supplementary file 2 — Reporting Summary [file 41587_2021_1006_MOESM2_ESM.pdf]

nature research

Corresponding author(s): John Marioni

Last updated by author(s): 29/6/21

Reporting Summary

Nature Research wishes to improve the reproducibility of the work that we publish. This form provides structure for consistency and transparency in reporting. For further information on Nature Research policies, see our Editorial Policies and the Editorial Policy Checklist.

Statistics

For all statistical analyses, confirm that the following items are present in the figure legend, table legend, main text, or Methods section.

n/a	Confirmed	
☐	☒	The exact sample size (n) for each experimental group/condition, given as a discrete number and unit of measurement
☐	☒	A statement on whether measurements were taken from distinct samples or whether the same sample was measured repeatedly
☐	☒	The statistical test(s) used AND whether they are one- or two-sided<br>*Only common tests should be described solely by name; describe more complex techniques in the Methods section.*
☐	☒	A description of all covariates tested
☐	☒	A description of any assumptions or corrections, such as tests of normality and adjustment for multiple comparisons
☐	☒	A full description of the statistical parameters including central tendency (e.g. means) or other basic estimates (e.g. regression coefficient) AND variation (e.g. standard deviation) or associated estimates of uncertainty (e.g. confidence intervals)
☐	☒	For null hypothesis testing, the test statistic (e.g. F, t, r) with confidence intervals, effect sizes, degrees of freedom and P value noted<br>*Give P values as exact values whenever suitable.*
☒	☐	For Bayesian analysis, information on the choice of priors and Markov chain Monte Carlo settings
☒	☐	For hierarchical and complex designs, identification of the appropriate level for tests and full reporting of outcomes
☐	☒	Estimates of effect sizes (e.g. Cohen's d, Pearson's r), indicating how they were calculated

Our web collection on statistics for biologists contains articles on many of the points above.

Software and code

Policy information about availability of computer code

Data collection	For data collection μManager version 1.4.21 and Zen Black 2.1 SP3 was used.
Data analysis	Scripts for data analysis are available at https://github.com/MarioniLab/SpatialMouseAtlas2020.<br><br>Analysis was performed using R version 3.6.1, and packages scran (version 1.14.6), MouseGastrulationData (version 1.0.0), BiocNeighbors (version 1.4.1), destiny (version 3.0.1), dynamicTreeCut (version 1.63-1), princurve (version 2.1.4), and scHOT (version 1.4.0). Image were processed using ImageJ version: 2.1.0/1.53c and Ilastik version: 1.3.0.

For manuscripts utilizing custom algorithms or software that are central to the research but not yet described in published literature, software must be made available to editors and reviewers. We strongly encourage code deposition in a community repository (e.g. GitHub). See the Nature Research guidelines for submitting code & software for further information.

Data

Policy information about availability of data

All manuscripts must include a data availability statement. This statement should provide the following information, where applicable:

- Accession codes, unique identifiers, or web links for publicly available datasets
- A list of figures that have associated raw data
- A description of any restrictions on data availability

The spatial transcriptomic map can be explored interactively at:
https://marionilab.cruk.cam.ac.uk/SpatialMouseAtlas/ and raw image data is available on
request. Processed gene expression data with segmentation information and associated metadata
is also available to download and explore online at

https://marionilab.cruk.cam.ac.uk/SpatialMouseAtlas/.

Processed gene expression data is also available within the R/Bioconductor data package MouseGastrulationData (version 3.13, https://bioconductor.org/packages/release/data/experiment/html/MouseGastrulationData.html).

Field-specific reporting

Please select the one below that is the best fit for your research. If you are not sure, read the appropriate sections before making your selection.

☒ Life sciences ☐ Behavioural & social sciences ☐ Ecological, evolutionary & environmental sciences

For a reference copy of the document with all sections, see nature.com/documents/nr-reporting-summary-flat.pdf

Life sciences study design

All studies must disclose on these points even when the disclosure is negative.

Sample size	No statistical methods were used to pre-determine sample size. Post hoc analysis showing high reproducibility and the agreement of cell type clusters with literature indicate that the sample size is sufficient.
Data exclusions	No raw data was excluded from the analyses. For downstream analysis, some segmented regions were excluded due to pre-established criteria for detection of single cells. Details are included in the Methods section.
Replication	We observed high concordance among biological replicates in terms of gene expression distribution and proportions of cell types. We used three biological replicate samples across two independent imaging experiments.
Randomization	The samples were not randomized in this study because only high quality samples/mice were used for the same experimental condition. There were no experimental conditions in our observational study.
Blinding	There is no experimental group in this study and hence no blinding is needed.

Reporting for specific materials, systems and methods

We require information from authors about some types of materials, experimental systems and methods used in many studies. Here, indicate whether each material, system or method listed is relevant to your study. If you are not sure if a list item applies to your research, read the appropriate section before selecting a response.

Materials & experimental systems

n/a	Involved in the study	
☐	☒	Antibodies
☒	☐	Eukaryotic cell lines
☒	☐	Palaeontology and archaeology
☐	☒	Animals and other organisms
☒	☐	Human research participants
☒	☐	Clinical data
☒	☐	Dual use research of concern

Methods

n/a	Involved in the study	
☒	☐	ChIP-seq
☒	☐	Flow cytometry
☒	☐	MRI-based neuroimaging

Antibodies

Antibodies used	Anti-pan Cadherin (Abcam, ab22744), anti-N-Cadherin (Cell Signaling Technology, [13A9], 14215), anti-β-Catenin antibody (15B8) (Abcam, ab6301), and anti-E-Cadherin antibody (BD Biosciences, clone 36, 610181)
Validation	All primary antibodies were purchased and validated by manufacturers as follows:<br><br>Goat anti-Mouse IgG (H+L) Superclonal Secondary Antibody: Invitrogen # A28174<br><br>Anti-N-Cadherin (Cell Signaling Technology, [13A9], 14215):<br>https://www.cellsignal.co.uk/products/primary-antibodies/n-cadherin-13a9-mouse-mab/14215<br><br>Validated for: Western Blotting, Immunoprecipitation, Immunofluorescence<br>Species Reactivity: Human, Mouse, Rat, Monkey<br><br>Anti-pan Cadherin (Abcam, ab22744):<br>https://www.abcam.com/pan-cadherin-antibody-mabcam22744-ab22744.html

Validated for: Western Blotting, Flow Cytometry, Immunofluorescence
Specifies Reactivity: Mouse, Rat, Human, African green monkey

Anti-β-Catenin antibody (15B8) (Abcam, ab6301):
https://www.abcam.com/beta-catenin-antibody-15b8-ab6301.html

Validated for: Western Blotting and knockout validated
Species Reactivity: Human, Mouse, Rat

Anti-E-Cadherin antibody (BD Biosciences, clone 36, 610181):
https://www.bdbiosciences.com/us/applications/research/stem-cell-research/cancer-research/human/purified-mouse-anti-e-cadherin-36e-cadherin/p/610181

Validated for: Western Blotting, Immunofluorescence, Immunohistochemistry
Species Reactivity: Human, Mouse, Rat, Dog

Animals and other organisms

Policy information about studies involving animals; ARRIVE guidelines recommended for reporting animal research

Laboratory animals	8-12 week wild-type C57BL/6J mice (Charles Rivers) were used, with exception of the HCR experiment (see below). For the HCR experiment, 4–6-week-old virgin wild-type CD-1 female mice and CD-1 male mice (Charles Rivers) were used.<br><br>We used C57BL/6J and CD-1 embryos at 8.5 days post fertilization. Sex was unknown at the time of collection due to early embryonic stage.
Wild animals	No wild animals were used in this study.
Field-collected samples	No field-collected samples were used in this study.
Ethics oversight	Experiments, with exception of the HCR experiment (see below), were performed in accordance with EU guidelines for the care and use of laboratory animals, and under authority of appropriate UK governmental legislation.<br><br>For HCR experiments the mice were maintained in accordance with guidelines from Memorial Sloan Kettering Cancer Center (MSKCC) Institutional Animal Care and Use Committee (IACUC) under protocol no. 03-12-017 (principal investigator A.-K.H.).

Note that full information on the approval of the study protocol must also be provided in the manuscript.
